# Aptamer-Based Multiplexed Proteomic Technology for Biomarker Discovery

**DOI:** 10.1371/journal.pone.0015004

**Published:** 2010-12-07

**Authors:** Larry Gold, Deborah Ayers, Jennifer Bertino, Christopher Bock, Ashley Bock, Edward N. Brody, Jeff Carter, Andrew B. Dalby, Bruce E. Eaton, Tim Fitzwater, Dylan Flather, Ashley Forbes, Trudi Foreman, Cate Fowler, Bharat Gawande, Meredith Goss, Magda Gunn, Shashi Gupta, Dennis Halladay, Jim Heil, Joe Heilig, Brian Hicke, Gregory Husar, Nebojsa Janjic, Thale Jarvis, Susan Jennings, Evaldas Katilius, Tracy R. Keeney, Nancy Kim, Tad H. Koch, Stephan Kraemer, Luke Kroiss, Ngan Le, Daniel Levine, Wes Lindsey, Bridget Lollo, Wes Mayfield, Mike Mehan, Robert Mehler, Sally K. Nelson, Michele Nelson, Dan Nieuwlandt, Malti Nikrad, Urs Ochsner, Rachel M. Ostroff, Matt Otis, Thomas Parker, Steve Pietrasiewicz, Daniel I. Resnicow, John Rohloff, Glenn Sanders, Sarah Sattin, Daniel Schneider, Britta Singer, Martin Stanton, Alana Sterkel, Alex Stewart, Suzanne Stratford, Jonathan D. Vaught, Mike Vrkljan, Jeffrey J. Walker, Mike Watrobka, Sheela Waugh, Allison Weiss, Sheri K. Wilcox, Alexey Wolfson, Steven K. Wolk, Chi Zhang, Dom Zichi

**Affiliations:** 1 SomaLogic, Boulder, Colorado, United States of America; 2 Department of Molecular, Cellular, and Developmental Biology, University of Colorado, Boulder, Colorado, United States of America; 3 The Rogosin Institute and the Weill Medical College of Cornell University, New York, New York, United States of America; 4 Department of Chemistry and Biochemistry, University of Colorado, Boulder, Colorado, United States of America; University of Milan-Bicocca, Italy

## Abstract

**Background:**

The interrogation of proteomes (“proteomics”) in a highly multiplexed and efficient manner remains a coveted and challenging goal in biology and medicine.

**Methodology/Principal Findings:**

We present a new aptamer-based proteomic technology for biomarker discovery capable of simultaneously measuring thousands of proteins from small sample volumes (15 µL of serum or plasma). Our current assay measures 813 proteins with low limits of detection (1 pM median), 7 logs of overall dynamic range (∼100 fM–1 µM), and 5% median coefficient of variation. This technology is enabled by a new generation of aptamers that contain chemically modified nucleotides, which greatly expand the physicochemical diversity of the large randomized nucleic acid libraries from which the aptamers are selected. Proteins in complex matrices such as plasma are measured with a process that transforms a signature of protein concentrations into a corresponding signature of DNA aptamer concentrations, which is quantified on a DNA microarray. Our assay takes advantage of the dual nature of aptamers as both folded protein-binding entities with defined shapes and unique nucleotide sequences recognizable by specific hybridization probes. To demonstrate the utility of our proteomics biomarker discovery technology, we applied it to a clinical study of chronic kidney disease (CKD). We identified two well known CKD biomarkers as well as an additional 58 potential CKD biomarkers. These results demonstrate the potential utility of our technology to rapidly discover unique protein signatures characteristic of various disease states.

**Conclusions/Significance:**

We describe a versatile and powerful tool that allows large-scale comparison of proteome profiles among discrete populations. This unbiased and highly multiplexed search engine will enable the discovery of novel biomarkers in a manner that is unencumbered by our incomplete knowledge of biology, thereby helping to advance the next generation of evidence-based medicine.

## Introduction

Proteins present in blood are an immediate measure of an individual's phenotype and state of wellness. Secreted proteins, released from diseased cells and surrounding tissues, contain important biological information with the potential to transform early diagnostic, prognostic, therapeutic, and even preventative decisions in medicine.

We will realize the full power of proteomics only when we can measure and compare the proteomes of many individuals to identify biomarkers of human health and disease and track the blood-based proteome of an individual over time. Because the human proteome contains an estimated 20,000 proteins – plus splicing and post-translational variants – that span a concentration range of ∼12 logs, identifying and quantifying valid biomarkers is a great technical challenge. Proteomic measurements demand extreme sensitivity, specificity, dynamic range, and accurate quantification.

The desire to profile the changes in protein expression at large scale is not new. Attempts at high-content proteomics began with 2-D gels and now mostly employ mass spectrometry (MS) and antibody-based technologies [1], [2]. MS can deliver specific analytical capabilities and the technology has advanced remarkably in the past decade. Despite great promise for MS in clinical proteomics, many challenges remain including issues of sensitivity (typically nM in current approaches), specificity, reproducibility, throughput, and cost [2]–[9].

Antibody-based methods are more sensitive than 2-D gels or MS and can detect analytes in the sub-nM range due to the high affinity of antibodies for their targets (typically nM to pM). However, non-specific binding of antibodies to non-cognate proteins, other macromolecules, and surfaces requires sandwich-type assays where the second antibody contributes to enhanced specificity through an independent binding event. In other words, technologies such as Enzyme-Linked Immuno-Sorbent Assays (ELISAs) attain high sensitivity by combining the specificity of two different antibodies to the same protein, requiring that both bind to elicit a signal [1]. Although broadly used in single-analyte tests, it has recently become clear that such assays cannot be multiplexed above a few tens of simultaneous measurements [10], [11] in large part because cross-reactivity of secondary antibodies to surface-immobilized proteins (including primary antibodies) dramatically erodes specificity [1]. This inherent characteristic compromises the performance of antibody-based arrays including printed antibodies, sandwich formats, and bead-based arrays [1], [12]. A recently reported proximity ligation assay that relies on antibody sandwich formation in solution followed by ligation of antibody-tethered nucleic acids and PCR amplification has been multiplexed with six analytes [10].

To address these challenges, we set out to develop a proteomics array technology analogous to the highly successful nucleic acid hybridization microarray. To create this technology, we developed a new class of DNA-based aptamers enabled by a versatile chemistry technology that endows nucleotides with protein-like functional groups. These modifications greatly expand the repertoire of targets accessible to aptamers. The resulting technology provides efficient, large-scale selection of exquisite protein-binding reagents selected specifically for use in highly-multiplexed proteomics arrays. Aptamers are a class of nucleic acid-based molecules discovered twenty years ago [13], [14] and have since been employed in diverse applications including therapeutics [15], catalysis [16], and now proteomics. Aptamers are short single-stranded oligonucleotides, which fold into diverse and intricate molecular structures that bind with high affinity and specificity to proteins, peptides, and small molecules [17]–[19]. Aptamers are selected *in vitro* from enormously large libraries of randomized sequences by the process of Systematic Evolution of Ligands by EXponential enrichment (SELEX) [13], [14]. A SELEX library with 40 random sequence positions has 4^40^ (∼10^24^) possible combinations and a typical selection screens 10^14^–10^15^ unique molecules. This is on the order of 10^5^ times larger than standard peptide or protein combinatorial molecular libraries [20].

Based on the collective knowledge of the aptamer field that has developed since its inception [13], [14], we hypothesized that aptamers could make exceptional reagents for high-content proteomics. There were many examples of high affinity RNA and DNA aptamers selected against human proteins [19]. However, there were also examples of difficult protein targets for which standard RNA and DNA SELEX did not yield high affinity aptamers. With two key innovations, we created a new class of aptamer, the Slow Off-rate Modified Aptamer (SOMAmer), which enabled efficient selection of high-affinity aptamers for almost any protein target and the development of a novel highly-multiplexed assay for high-performance proteomics. Here we present the development of these unique reagents in the context of our high-content, high-performance, low-cost proteomics array, and demonstrate the potential of the platform to identify biomarkers from clinically-relevant samples.

## Results

### SELEX with chemically-modified nucleotides

**Figure 1:**
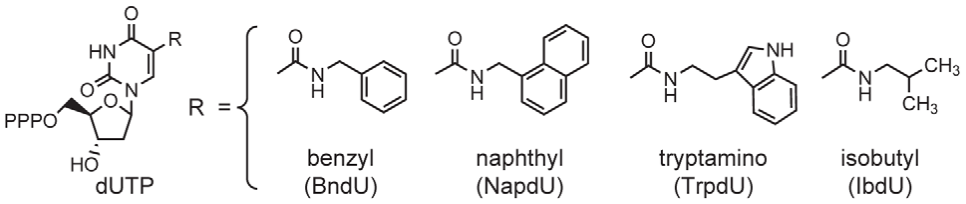
Modified Nucleotides. Nucleotide triphosphate analogs modified at the 5-position (R) of uridine (dUTP): 5-benzylaminocarbonyl-dU (BndU); 5-naphthylmethylaminocarbonyl-dU (NapdU): 5-tryptaminocarbonyl-dU (TrpdU); and 5-isobutylaminocarbonyl-dU (iBudU).

The first innovation in developing the SOMAmer was motivated by the idea that aptamers can be endowed with protein-like properties by adding functional groups that mimic amino acid side-chains to expand their chemical diversity [21]. Eaton and colleagues developed the technology to efficiently synthesize nucleotides modified with diverse functional groups and to utilize them in SELEX [21], [22]. This innovation was used to select catalysts, including the first RNA-catalyzed carbon-carbon bond formation [16], [23]. Building on this work, we developed modified deoxyribonucleotides and SELEX methods [24] to select modified DNA aptamers from libraries that incorporate one of four dUTPs modified at the 5-position (Figure 1). This included the synthesis of random libraries with modified nucleotides and the enzymatic amplification of SELEX pools that contain modified nucleotides (see Materials and Methods).

**Figure 2:**
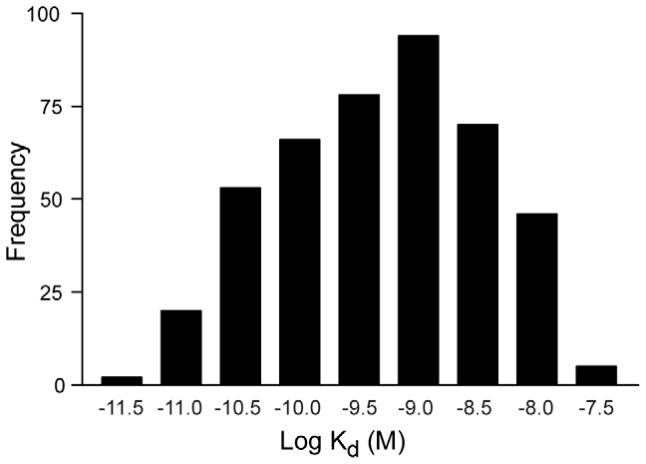
Dissociation constants. Distribution of dissociation constant (K_d_) values for 434 SOMAmers.

To test whether modified nucleotides improve SELEX, we compared selections with modified and unmodified nucleotides targeting thirteen “difficult” human proteins that had repeatedly failed SELEX with unmodified DNA. As a control, we included GA733-1 protein, which had yielded high-affinity aptamers with unmodified DNA SELEX. The results (Table 1) show that only SELEX with modified nucleotides yielded high-affinity aptamers to these difficult proteins. Different modifications worked better with different proteins. This shows that applying multiple modifications to the same target ensures a higher probability of success. Based on these results, we adopted modified nucleotide SELEX exclusively in our standard selections. To date, we have selected high-affinity aptamers (with most K_d_ values lower than nM, see Figure 2) to over 1000 human proteins, nearly all the proteins we have targeted. There are no obvious commonalities among those proteins that were initially unsuccessful in SELEX with unmodified DNA.

**Table 1:** SELEX library affinities (K_d_, (M)) with unmodified and modified nucleotides.

Target Protein	dT	benzyl-dU	isobutyl-dU	tryptamino-dU
4-1BB§	failed	6×10^−9^	Failed	4×10^−9^
B7§	failed	1×10^−8^	Failed	7×10^−9^
B7-2§	failed	Failed	Failed	6×10^−9^
CTLA-4§	failed	Failed	Failed	1×10^−9^
sE-Selectin§	failed	Failed	Failed	2×10^−9^
Fractalkine/CXC3L-1	failed	Failed	Failed	5×10^−11^
GA733-1 protein§	9×10^−9^	3×10^−9^	5×10^−9^	5×10^−10^
gp130, soluble§	failed	6×10^−9^	2×10^−8^	1×10^−9^
HMG-1	failed	Failed	2×10^−8^	5×10^−9^
IR	failed	2×10^−9^	1×10^−8^	2×10^−10^
Osteoprotegrin§	failed	5×10^−9^	9×10^−9^	2×10^−10^
PAI-1	failed	4×10^−10^	9×10^−10^	2×10^−10^
P-Cadherin§	failed	4×10^−9^	5×10^−9^	3×10^−9^
sLeptin R§	failed	2×10^−9^	Failed	5×10^−10^

§ The protein used was expressed as a fusion to the Fc of human IgG1. No detectable binding of the active library to an alternate Fc fusion proetin was observed.

We first implemented 5-benzylaminocarbonyl-dU (BndU) in our high-throughput SELEX pipeline, and our success rate for selections to human proteins rose from <30% to >50% to a diversity of human proteins. This supported our hypothesis that we could develop one SELEX protocol that would work repeatedly for very different proteins. Since then, we have incorporated four modified nucleotides, BndU, 5-naphthylmethylaminocarbonyl-dU (NapdU), 5-tryptaminocarbonyl-dU (TrpdU), and 5-isobutylaminocarbonyl-dU (iBudU). Since the incorporation of these modified nucleotides into SELEX experiments, our overall success rate (pool K_d_ <∼30 nM) is ∼84% (1204/1428) for high quality SOMAmers to a wide range of human proteins. The 813 human proteins measured by the current array are shown in Table S1. Overall, these results provide the first comprehensive evidence that modified nucleotides can expand the range of possible aptamer targets and improve their binding properties.

### SELEX for slow off-rates

The second innovation was a solution to the principal challenge of identifying a second element of specificity beyond binding of a second ligand for use in high-content arrays. Inspired by classic kinetic theory of specific binding in complex mixtures [25], [26], we employed kinetic manipulations to help overcome the problem of non-specific SOMAmer-protein binding. To achieve this second element of specificity, we selected for aptamers with slow dissociation rates (t_1/2_ >30 min) that allow selective disruption of non-specific (or non-cognate) binding interactions by using a large excess of a polyanionic competitor.

**Figure 3:**
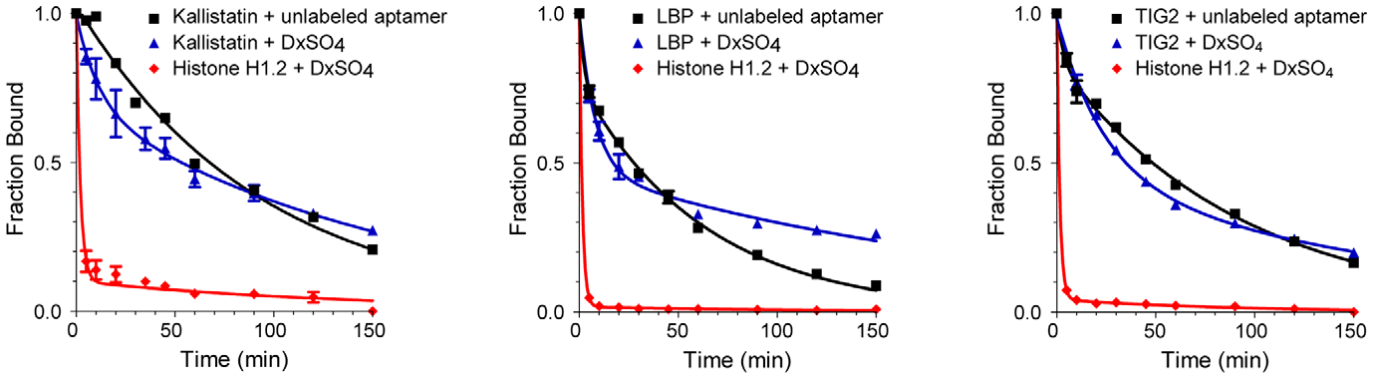
Kinetic discrimination between cognate and non-cognate interactions. Dissociation rate measurements for specific and non-specific protein interactions with representative Kallistatin, LBP, and TIG2 SOMAmers. Histone H1.2 binds to random DNA sequences and was used to demonstrate non-specific binding. The fraction of radiolabeled SOMAmer (10 pM) bound to its cognate target is shown after addition of 50 nM unlabeled SOMAmer (squares) or 0.3 mM dextran sulfate (triangles) as a function of time. Rapid dissociation of non-specific complexes in the presence of 0.3 mM dextran sulfate is also shown (diamonds).

**Figure 4:**
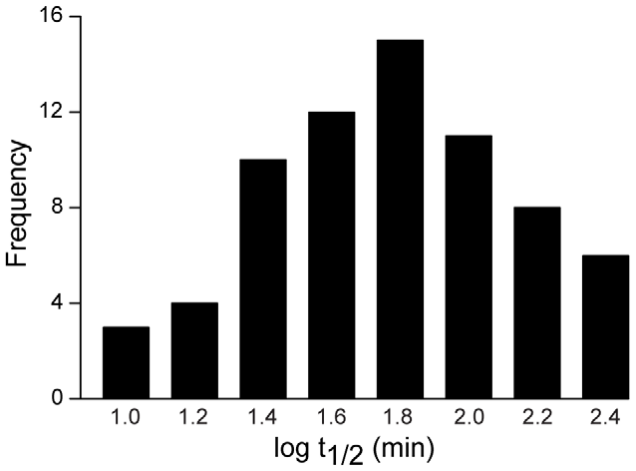
Dissociation rates. Distribution of dissociation rate (t_1/2_) values for 72 SOMAmers representative of those in proteomic arrays.

For example, Figure 3 shows the half-life of dissociation of kallistatin, LBP, and TIG2 SOMAmers from their cognate targets (determined by using unlabeled SOMAmers) are 65, 44, and 65 minutes, compared to <1 minute for dissociation of the same SOMAmers from histone H1.2, a known DNA binding protein. Specific interactions are disrupted to a far lesser degree by dextran sulfate for all three SOMAmers. Figure 4 shows a representative distribution of dissociation half-lives for SOMAmers selected for our multiplexed proteomics assay.

### SOMAmer specificity

**Figure 5:**
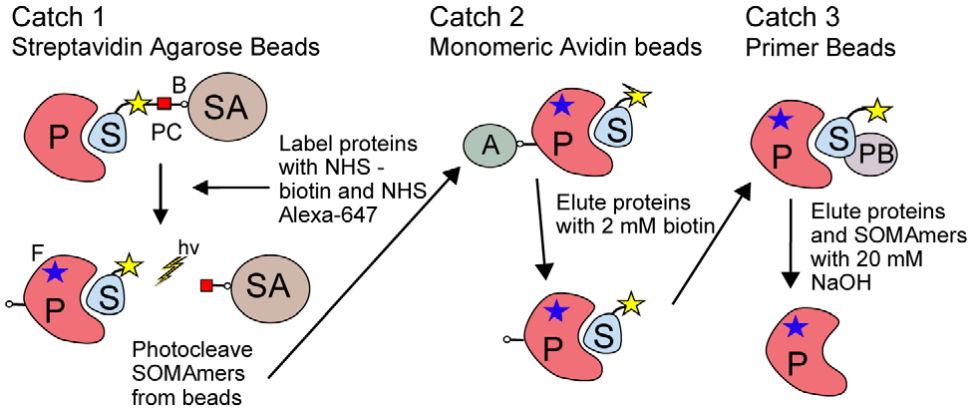
Affinity capture assay. SOMAmers are mixed with the target sample (purified protein or plasma) and incubated to bind to equilibrium. In **Catch-1** bound SOMAmer(S)-protein(P) complexes are captured onto streptavidin beads (SA) and the proteins are tagged with biotin (B) (NHS- biotin) and fluorescent label (F) (NHS Alexa 647). Unbound proteins are washed away. Bound complexes are released from the beads by cleaving the photo-cleavable linker (PC) with ultraviolet light. In **Catch-2** SOMAmer-protein complexes are captured onto monomeric avidin beads (A), washed, and eluted from the beads with 2 mM biotin. At this stage, SOMAmer-protein complexes are subjected to a kinetic challenge analogous to that used in the proteomics assay. Specific complexes survive the challenge and non-specific complexes dissociate. In the final step, **Catch-3**, bound complexes are captured onto primer beads (PB) by DNA primer that is complementary to a portion of the SOMAmer and any remaining unbound protein resulting from the kinetic challenge is washed away. Finally, the captured complexes are dissociated with 20 mM NaOH and the target protein is eluted for analysis by PAGE.

**Figure 6:**
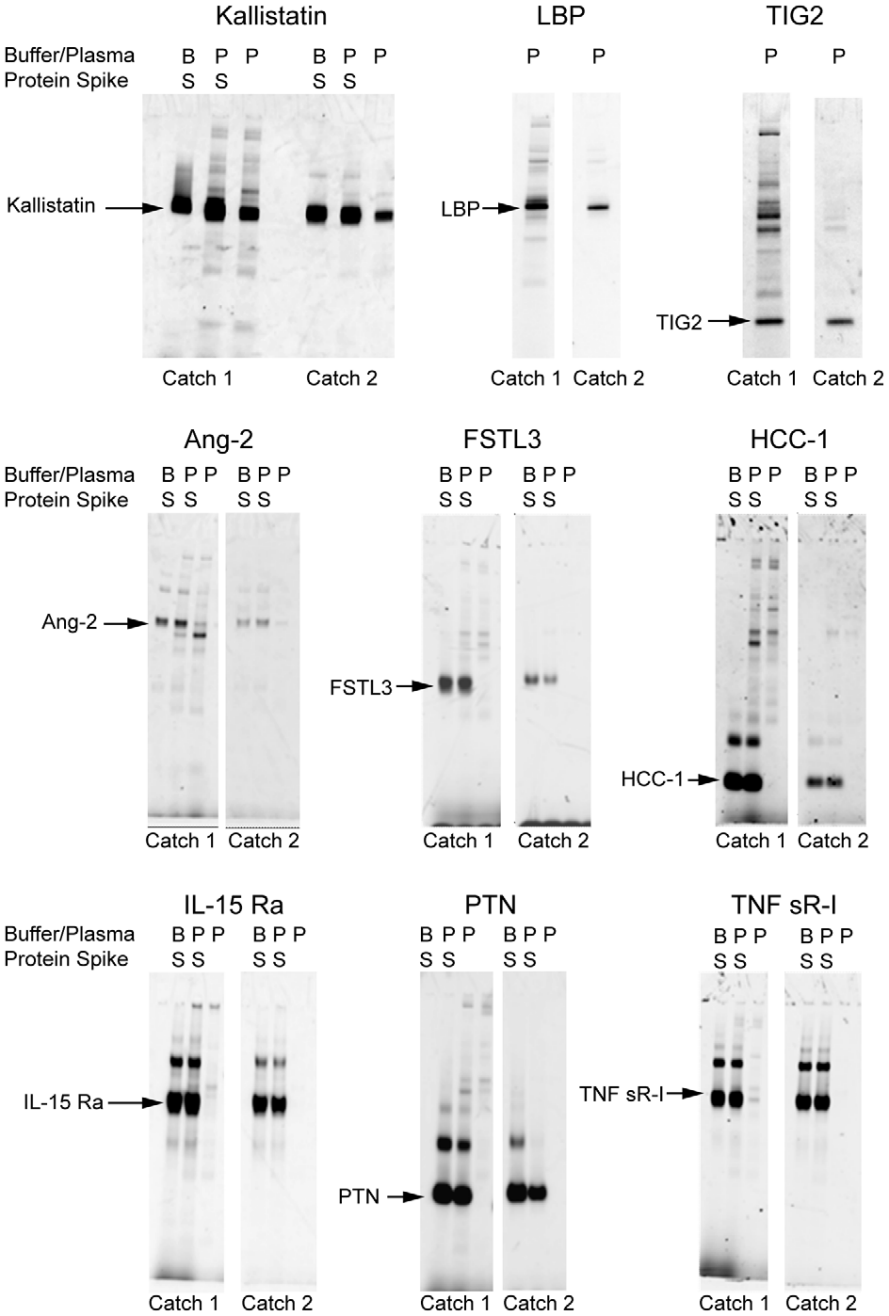
Affiinity capture of representative SOMAmer protein targets. SDS-PAGE visualization of representative SOMAmer protein targets p Kallistatin, LBP and TIG2. The Kallistatin gel shows proteins bound to the Kallistatin SOMAmer for target added to buffer (lane 1), target added to 10% plasma (lane 2), and 10% plasma alone (lane 3). The first set of three lanes demonstrates all of the proteins eluted from Catch-1 beads. The second set of lanes shows the SOMAmer-bound proteins eluted from Catch-2 beads. The LBP and TIG2 gels demonstrate proteins recovered from 10% plasma using the LBP and TIG2 SOMAmers, respectively (without adding proteins to plasma for these three gels). The endogenous plasma proteins captured by the Kallistatin, LBP, and TIG2 SOMAmers were identified by LC-MS/MS as the intended target proteins (Table 2). The remaining gels show affinity capture assays for CKD-related proteins. For each example the gel shows the results for purified target protein spiked into buffer (lane 1), purified target protein spiked into 10% plasma (lane 2), and 10% plasma (lane 3). The first set of three lanes demonstrates all of the proteins eluted from Catch-1 beads. The second set of lanes shows the aptamer-bound proteins eluted from Catch-2 beads.

The assay uses one SOMAmer per analyte rather than a sandwich of binding reagents and thus depends on equilibrium binding and kinetics for specificity. The difference in dissociation rates between cognate and non-cognate interactions contributes significantly to specificity in the assay (Figure 3). The use of sequential capture of protein-SOMAmer complexes on two sets of streptavidin beads, first through biotin-labeled SOMAmers (Catch-1) and then through biotin-labeled proteins (Catch-2), substantially reduces non-specific interactions. We assessed the specificity of select SOMAmers for the targets they were selected against in an affinity binding assay that mimics our multiplexed proteomics assay. The experimental method is outlined in Figure 5 and detailed in Materials and Methods. This experiment mimics Catch-1 and Catch-2 in the proteomics assay and then uses a third step to capture the bound SOMAmer-protein complex with an oligo that is complementary to a portion of the SOMAmer and acts as an affinity tag. This “Catch-3” step is analogous to the DNA microarray hybridization step in the proteomics assay. The captured complexes are then disrupted and the proteins are eluted and analyzed by denaturing polyacrylamide gel electrophoresis (PAGE), as shown in Figure 6.

As shown in Figure 6, eluate from Catch-1 beads generally contains the target protein as well as several other proteins that bind SOMAmers non-specifically. Eluate from Catch-2 beads contains only the target protein in substantially pure form, along with its cognate SOMAmer (for these experiments, reversible protein attachment to monomeric avidin Catch-2 beads was used). This is likely due, in part, to a reduction in the amount of total protein following Catch-1 bead washing (only SOMAmer-bound or surface-bound proteins remain) as well as to release and recapture of complexes on separate beads in a reversed orientation (attachment through biotin on proteins).

To further assess the specificity of selected (>20) SOMAmers for their target proteins, we excised the resulting PAGE gel samples (entire lanes) from the plasma affinity binding experiment described above and analyzed them by mass spectrometry (MS). In all cases, the results confirmed that the gel band contained the target protein (Table 2). This was evidenced by the identification of peptides that map to the target protein using their specific fragmentation patterns. Peptides that mapped to other proteins were also identified, although the number of spectra for these proteins was typically much lower than for the specific target. Such contaminants are a relatively small fraction because the PAGE gels show sharp uniform bands with very little background, which suggests that the majority of the material is the target protein.

**Table 2:** Identification by LC-MS/MS of affinity captured proteins.

Protein	Accession	Protein ID Probability	Unique Peptides	Unique Spectra	Total Spectra	%Sequence Coverage
Kallistatin	IPI00328609	1	22	31	39	53.6
LBP	IPI00032311	1	13	16	22	27.0
TIG2	IPI00019176	0.95	2	2	2	13.5

### Multiplex proteomic assay

**Figure 7:**
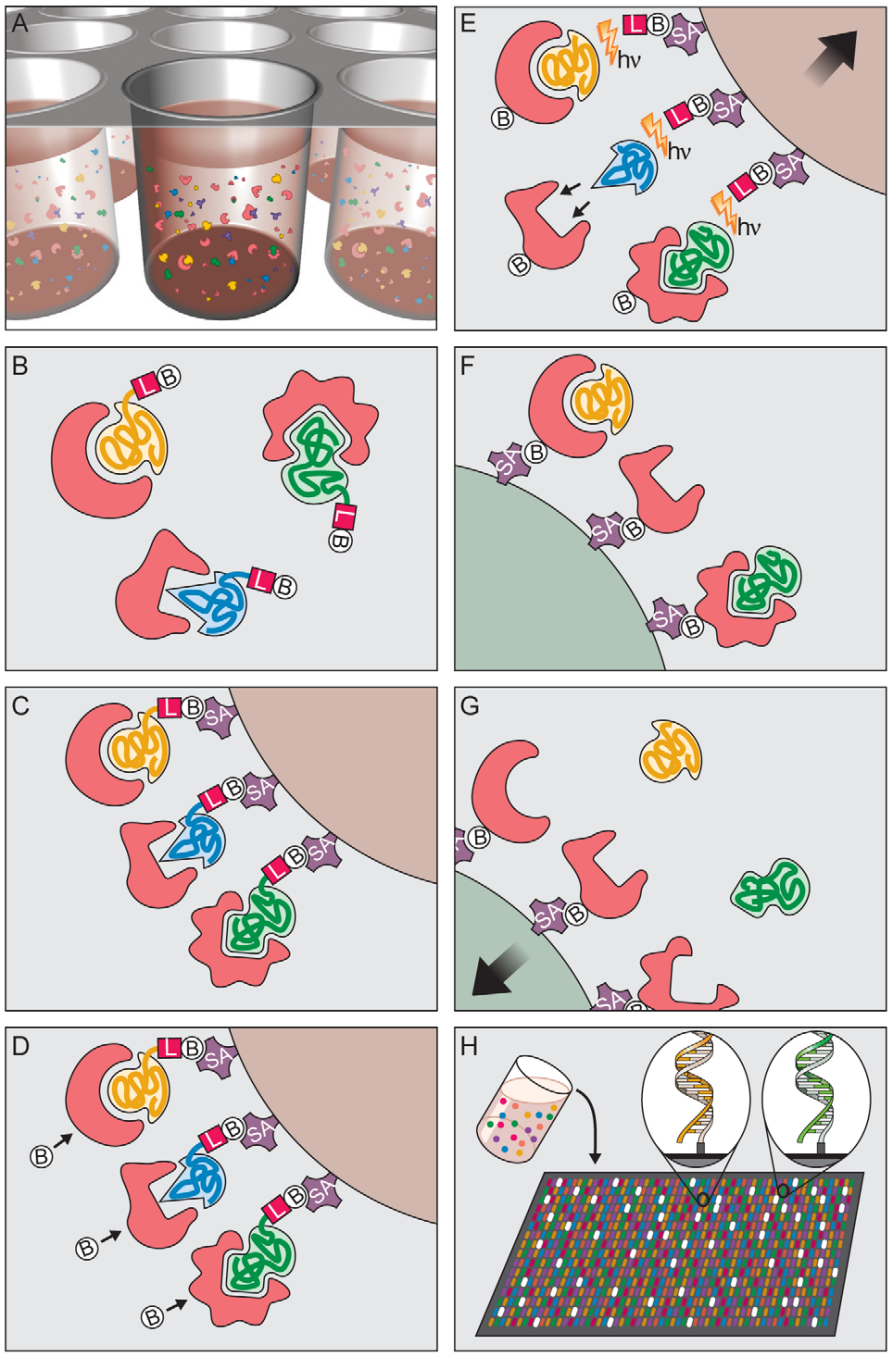
Principle of multiplex SOMAmer affinity assay. (A) Binding. SOMAmers and samples are mixed in 96-well microwell plates and allowed to bind. Cognate and non-cognate SOMAmer-target protein complexes form. Free SOMAmer and protein are also present. (B–H) Schematic sequence of assay steps leading to quantitative readout of target proteins. (B) SOMAmer-protein binding: DNA-based SOMAmer molecules (gold, blue, and green) have unique shapes selected to bind to a specific protein. SOMAmers contain biotin (B), a photo-cleavable linker (L) and a fluorescent tag at the 5′ end. Most SOMAmers (gold and green) bind to cognate proteins (red), but some (blue) form non-cognate complexes. (C) Catch-1. SOMAmers are captured onto a bead coated with streptavidin (SA) which binds biotin. Un-complexed proteins are washed away. (D) Proteins are tagged with NHS-biotin. (E) Photocleavage and kinetic challenge. UV light (hν) cleaves the linker and SOMAmers are released from beads, leaving biotin on bead. Samples are challenged with anionic competitor (dextran sulfate). Non-cognate complexes (blue SOMAmer) preferentially dissociate. (F) Catch-2 SOMAmer-protein complexes are captured onto new avidin coated beads by protein biotin tag. Free SOMAmers are washed away. (G) SOMAmers are released from complexes into solution at high pH. (H) Remaining SOMAmers are quantified by hybridization to microarray containing single-stranded DNA probes complementary to SOMAmer DNA sequence, which form a double-stranded helix. Hybridized SOMAmers are detected by fluorescent tags when the array is scanned.

To create a high-content proteomics platform for biomarker discovery, we developed a novel assay (Figure 7) which transforms a complex proteomic sample (*e.g.*, plasma, serum, conditioned media, cell lysates, *etc.*) into a quantified protein signature. The assay leverages equilibrium binding and kinetic challenge [1]. Both are carried out in solution, not on a surface, to take advantage of more favorable kinetics of binding and dissociation [1]. The kinetic challenge works well for two reasons. First, dissociation rates of non-cognate SOMAmer-protein interactions are generally much faster (half-lives of a few minutes or less). Second, since all aptamers are polyanions, another polyanion at high concentration, such as dextran sulfate, can serve as a common competitor that dramatically minimizes rebinding events in a multiplex assay. In contrast, a common non-denaturing competitor of all antibody-antigen interactions or, more generally, protein-protein interactions, is not known.

An overview of the assay is shown in Figure 7. Briefly, the sample is incubated with a mixture of SOMAmers each containing a biotin, a photocleavable group, and a fluorescent tag followed by capture of all SOMAmer-protein complexes on streptavidin beads (Catch-1) (Figure 7A–C). After stringent washing of the beads to remove unbound proteins and labeling of bead-associated proteins with biotin under controlled conditions (Figure 7D), the complexes are released from the beads back into solution by UV light irradiation and diluted into a high concentration of dextran sulfate, an anionic competitor. The biotin that was originally part of the SOMAmer remains on beads. The anionic competitor coupled with dilution selectively disrupts non-cognate complexes (see Figure 7E) and because only the proteins now contain biotin, the complexes are re-captured on a second set of beads (Catch-2) from which unbound SOMAmers are removed by a second stringent washing (Figure 7F). The SOMAmers that remain attached to beads are eluted under high pH-denaturing conditions and hybridized to sequence-specific complementary probes printed on a standard DNA microarray (Figure 7G,H).

**Figure 8:**
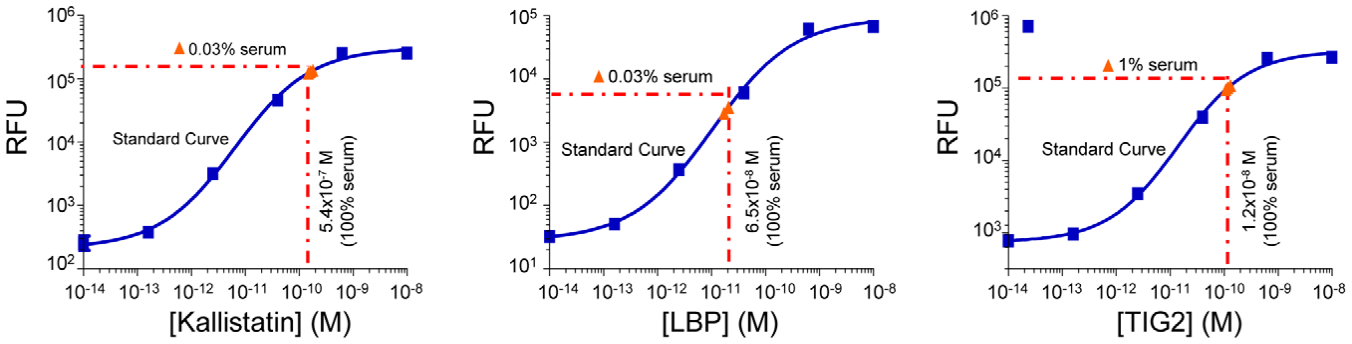
Proteomic assay standard curves. Each plot shows the standard curve for eight replicates of target spiked into buffer (blue squares). Triplicate measurements from diluted normal serum (red triangles, measured dilution indicated) are plotted onto the standard curve, and the calculated normal concentrations in 100% serum are shown.

The result is a mixture of SOMAmers that quantitatively reflects protein concentrations in the original sample. The modified nucleotides in SOMAmers are designed to maintain canonical base-pairing [24], [27] (in a DNA duplex, adducts at the 5-position of pyrimidines are directed toward the major groove of DNA) and hybridize effectively to unmodified DNA oligonucleotides on the array (this is also required for replication during SELEX). The capture of SOMAmers on a hybridization array permits quantitative determination of the protein present in the original sample by converting the assay signal (relative fluorescence units, RFUs) to analyte concentration (Figure 8). Thus, our assay takes advantage of the dual nature of aptamers as molecules capable of both folding into complex three-dimensional structures, which is the basis of their unique binding properties, and hybridization to specific capture probes.

### Target protein menu

**Figure 9:**
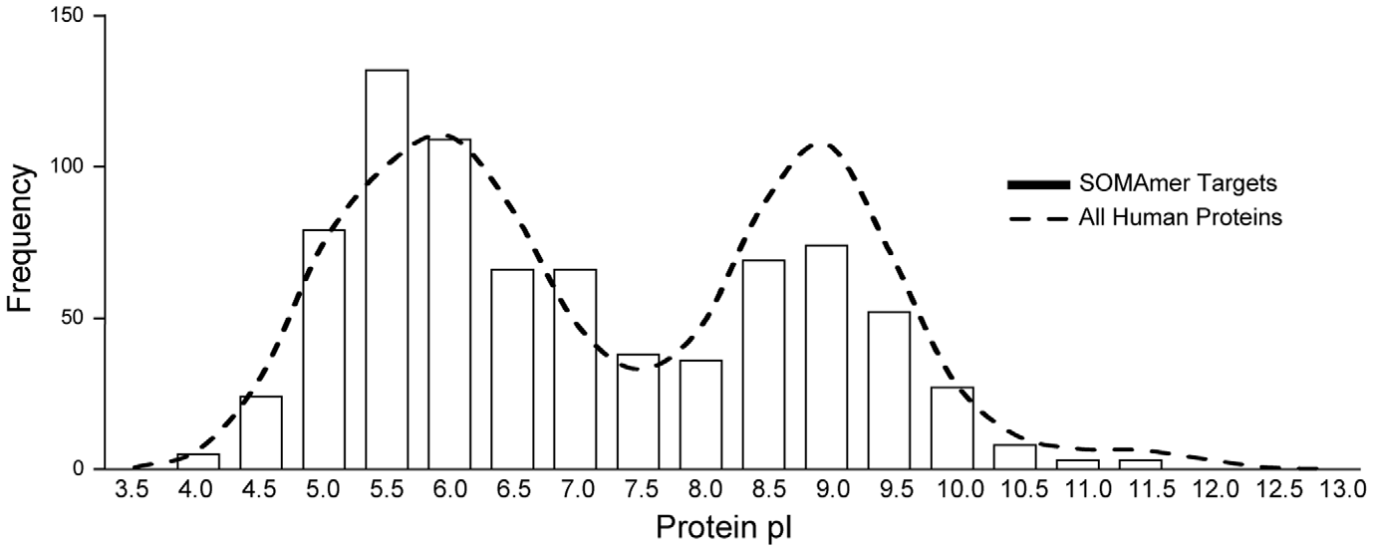
Target isoelectric points. Distribution of isoelectric points (pI) of proteins for which SOMAmers have been selected (bars) and of all human protein chains in UniProt (dashed line).

**Figure 10:**
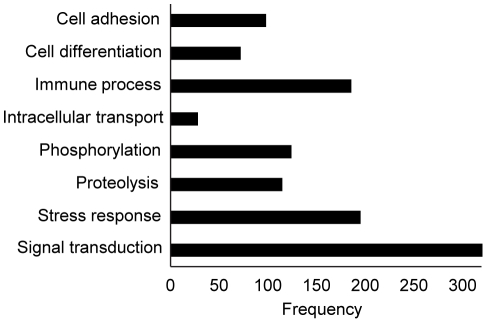
Protein target menu gene ontology. Distribution of most common gene ontology terms associated with the proteins measured by the current array.

The current version of our assay measures 813 human proteins (Table S1). These proteins represent a wide range of sizes, physicochemical properties, such as a pI range of 4-11 (Figure 9), and biological functions from a variety of molecular pathways and gene families (Figure 10). Thus, SOMAmer technology enables an efficient and scalable pipeline to generate unbiased content for proteomics arrays.

### Assay performance

#### Reproducibility

To assess the reproducibility of the assay, we collected serum and plasma samples from 18 healthy volunteers and assayed five replicates of each sample in a single run, and repeated this in triplicate. The results show an overall low median CV of ∼5% for intra-run and inter-run CV. The CV for each SOMAmer was computed for each sample by averaging over the replicates and then averaging these CVs over all the samples. Both intra- and inter-plate CVs were computed for each dilution mix and are shown in Figure S1.

#### Limits of quantification

To assess the quantitative performance of the assay, we measured the upper and lower limit of quantification (ULOQ and LLOQ) and dynamic range of quantification (ROQ) values for a representative subset (356) target proteins. The LOQ experiments measured six-point standard curves spanning six logs in concentration, from 10 nM to 10 fM, in buffer. Overall, the median LLOQ was ∼1 pM, with some as low as 100 fM, the median ULOQ was ∼1.5 nM, and the median ROQ was ∼3 logs (results summarized in Figure S2 and complete results reported in Table S2). We conducted these experiments in buffer because many target proteins have endogenous blood concentrations that prevent determining precision profiles and LOQs. For some proteins with low endogenous concentrations, we found consistent performance when titrated into 10% plasma (see below).

For each analyte, we generated a precision profile, which shows the variation in %CV for calculated concentration as a function of analyte concentration. Precision profiles provide an analytic measurement of assay performance and establish ULOQ, LLOQ, and ROQ. In some cases the measurements did not plateau in the upper measured range (10 nM) and the reported values represent a minimum estimate of ULOQ and resulting ROQ. A typical dose-response curve and precision profile from the data set is shown in Figure S3. We computed a full precision profile for each analyte using two methods. One method modeled the standard deviation for calculated concentrations σ*_x_* directly, and the other method modeled σ*_logRFU_* from which σ*_x_* was computed. For the example shown in Figure S3, both methods gave similar results. This particular analyte shows a remarkable five-log quantification range at a 20% CV cutoff with an LLOQ of 0.4–0.6 pM and a ULOQ of 40–50 nM. In general there is good agreement between the two different methods for computing precision profiles, and the assay response *σ_logRFU_* method was used to calculate the values shown in Table S2.

#### Buffer versus plasma LOQs

To compare assay performance for measuring analytes in buffer to measuring proteins in plasma, we measured LLOQs, ULOQs, and ROQs in buffer and plasma for twenty-eight analytes with low endogenous plasma concentrations. The results are summarized in Figure S4 and detailed in Table S3. Comparing these measurements, the average log ratio of plasma to buffer LLOQs and ULOQs was 0.26 and 0.32, which shows that the average LLOQ and ULOQ for these proteins was 2–3 fold higher in plasma than buffer. Of the 28 proteins compared, about two thirds (20/28) had somewhat higher LLOQs in plasma and about one third (8/28) had lower LLOQs in plasma compared to buffer. Overall, these results suggest that buffer precision profiles provide a reasonable assessment of the quantitative behavior of the multiplexed assay in plasma.

### Example: Chronic kidney disease

To demonstrate the utility of the platform to discover disease-related biomarkers, we analyzed plasma from subjects with chronic kidney disease (CKD), the slow loss of kidney function over time. CKD is a recently recognized global public health problem that is “common, harmful, and treatable” with an estimated prevalence of nearly 10% worldwide [28]. Early intervention in CKD can substantially improve prognosis, which is otherwise poor [28]–[31]. To achieve early diagnosis, predictive, non-invasive CKD biomarkers are needed. CKD biomarkers could also be useful for monitoring disease progression and guiding treatment [28]–[31].

We chose CKD as a test case because kidney physiology provides filtration of serum molecules based on size (molecular mass) and charge [32] – thus CKD might lead to an increase in the concentration of small proteins (MW <45 kDa). Disease progression is expected to be accompanied by an overall increase in plasma concentration of small proteins.

**Figure 11:**
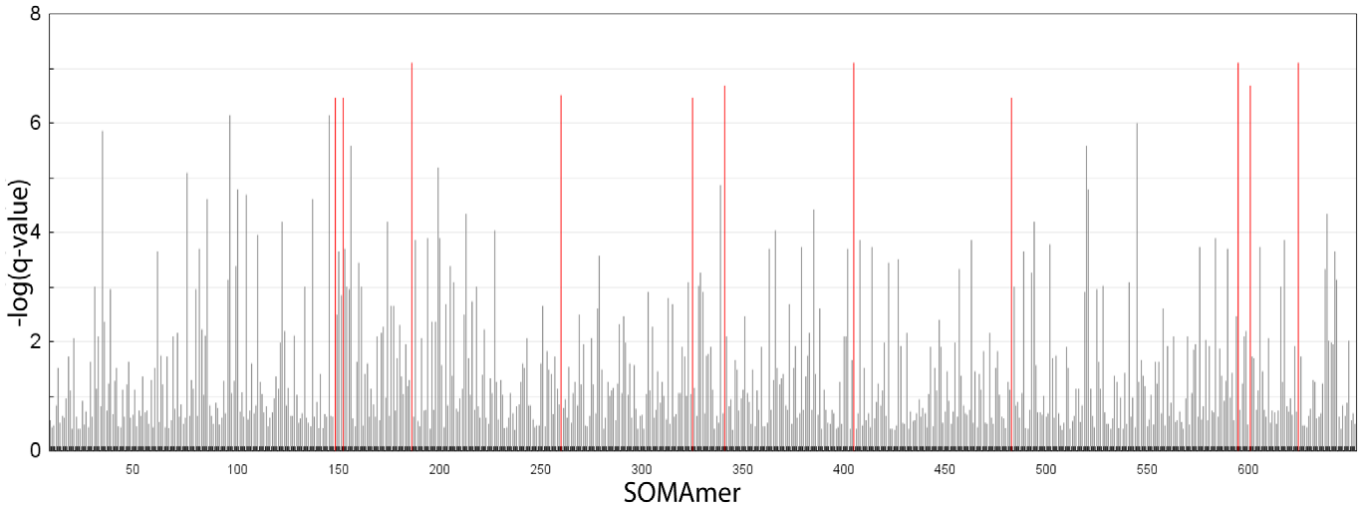
Biomarker discovery in CKD. Distribution of the false discovery rate (q-value) for the Mann-Whitney test statistic comparing late-stage *vs.* early-stage CKD for each protein measured (indicated as a bar on the x-axis) ordered arbitrarily.

We obtained and analyzed plasma samples from 42 subjects with CKD. Eleven subjects had early-stage CKD based on estimated GFR (Table 3) (eGFR, defined as stages 1–2, median creatinine clearance 70 ml/min/m^2^, range 62–97 ml/min/m^2^) and 31 had late-stage CKD (stages 3–5, median creatinine clearance 25 ml/min/m^2^, range 7–49 ml/min/m^2^) [33]. We measured 614 human proteins (array size at the time analyses were conducted) simultaneously for each sample and compared the results of early- to late-stage CKD (Figure 11).

**Table 3:** Population demographics for chronic kidney disease study.

	Early stage CKD	Late stage CKD
N (total = 42)	11	31
Gender %F (F/M)	33% (4/11)	45% (14/31)
Age (avg. yrs)	62 [51–68]	67 [57–77]
Wt. (avg. kg)	89 [73–98]	88 [75–104]
BMI (avg.)	30.5 [26.6–36.5]	31.8 [27.1–36.6]
eGFR (median) §	70 [62–97]	25 [7–49]

§ Estimated glomerular filtration from creatinine clearance (MDRD formula) ml/min/m^2^.

**Figure 12:**
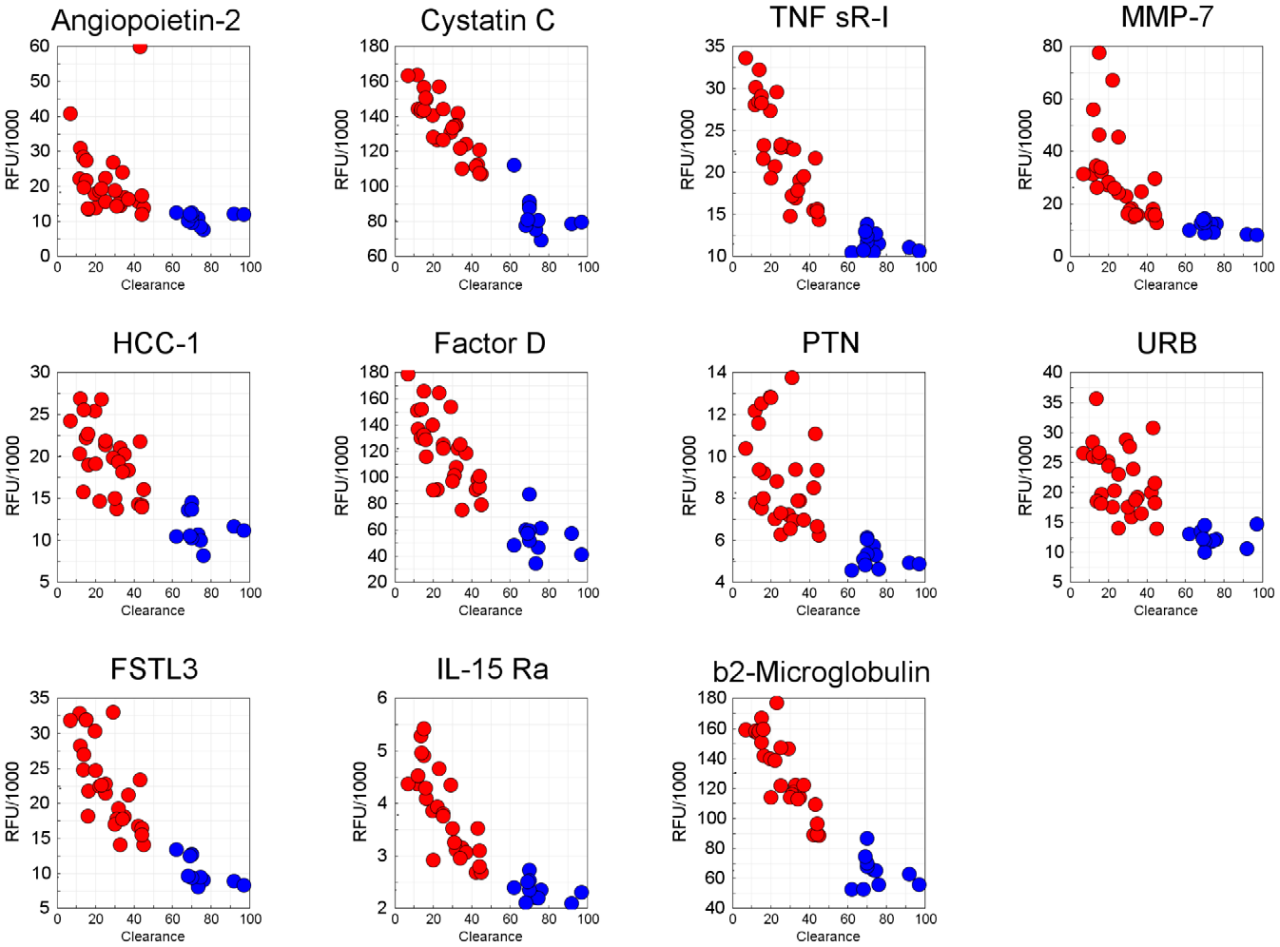
Potential CKD biomarkers. Eleven analytes with the smallest q-values (<3.5×10^−7^). Protein concentrations (expressed as RFU values) as a function of renal clearance for the eleven best biomarkers of late-stage (red circles) *vs.* early-stage CKD (blue circles).

We identified 60 proteins that varied significantly between the two groups, using the Mann-Whitney test, with a q-value (false discovery rate-corrected p-value) of 4.2×10^−4^ (Table S4). Eleven proteins with the most highly significant variation (q-values <3.5×10^−7^) are highlighted in Figure 12 and shown in Table 4. Nine out of eleven are relatively small proteins (<25 kDa). For all eleven proteins, there is an inverse correlation between eGFR (a marker of CKD progression) and protein concentration, suggesting they could be potential biomarkers of CKD progression (Figure 12). Two of the eleven proteins, cystatin C and β_2_-microglobulin, are important known biomarkers of CKD [29]–[31] and two additional proteins, complement factor D and TNF sR-I, have been reported to have elevated concentrations in CKD [34], [35].

**Table 4:** Top 11 Potential CKD Biomarkers§.

Target Protein	p-value	q-value	Mol. Mass (kDa)
β_2_-Microglobulin	1.2×10^−9^	8.0×10^−8^	11.7
FSTL3	1.2×10^−9^	8.0×10^−8^	25.0
Pleotrophin	1.2×10^−9^	8.0×10^−8^	15.3
TNF sR-I ‡, *	1.2×10^−9^	8.0×10^−8^	21.2
Factor D	4.8×10^−9^	2.1×10^−7^	24.4
IL-15 Rα ‡, †	4.8×10^−9^	2.1×10^−7^	25.0
MMP-7	8.4×10^−9^	3.2×10^−7^	19.1
Angiopoietin-2	1.4×10^−8^	3.5×10^−7^	54.9
Cystatin C	1.4×10^−8^	3.5×10^−7^	13.3
HCC-1‡	1.4×10^−8^	3.5×10^−7^	8.7
URB ‡	1.4×10^−8^	3.5×10^−7^	105.7

§ Based on q-value ranking.

‡ Smaller isoforms also exist. For example, URB has a 10.3 kDa isoform.

*Extracellular domain comprising amino acids 22–211.

† Extracellular domain is 18.4 kDa.

**Figure 13:**
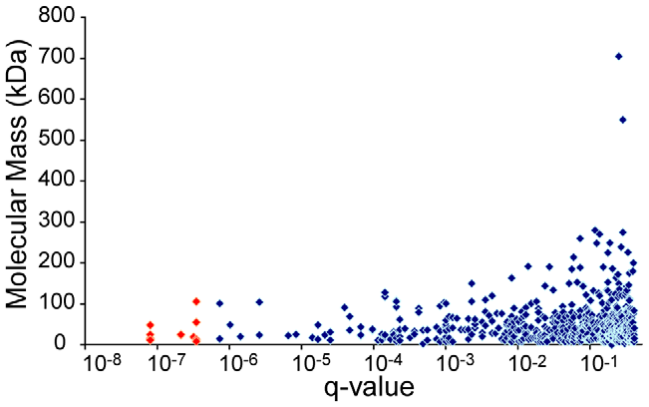
Comparison of a protein's molecular mass and the probability that it is a CKD biomarker (q-value (p-value corrected for false discovery rate)).

Accumulation in plasma of some small proteins appears to be a major change in the proteome. However, the concentration of many low molecular weight proteins did not change appreciably with disease progression (Figure 13); pI also was uncorrelated with an increase in plasma concentration as a function eGFR (data not shown). The surprising fact that the biomarkers are not simply ranked according to their molecular masses shows that reduced kidney function is complex. The accumulation of some (but not all) low molecular weight proteins, sometimes called “middle molecules”, in plasma of patients with impaired renal filtration has long been implicated in the pathology of kidney disease[36]. High-content proteomic analysis provides a means of unbiased discovery of such proteins and their relationship to disease progression.

## Discussion

We developed a new aptamer-based proteomic technology capable of measuring thousands of proteins in small volumes of biological samples with low limits of detection, a broad dynamic range, and high reproducibility. The current assay measures 813 proteins with 1 pM median LLOQ, 7-log overall dynamic range (∼100 fM–1 µM) with three sample dilutions that span ∼2.5 logs, and 5% median CV. The content of the discovery assay is flexible and highly scalable, permitting the addition of content as the target menu grows.

To achieve this performance, we developed a new class of DNA-based aptamer, the Slow Off-rate Modified Aptamer (SOMAmer), based on two key innovations: novel chemically-modified nucleotides that mimic amino acid side chains and new SELEX strategies to select aptamers with very slow off-rates. With this technology, our success rate for selecting high-quality aptamers to target proteins rose from <30% to >90% today. To date, we have selected high-quality SOMAmers to >1,000 human proteins.

We demonstrated the utility of our proteomics platform in biomarker discovery with a study of CKD. We identified 60 proteins that varied significantly between early and late stage CKD, which could provide a foundation for developing CKD diagnostics. In a study of more than 500 additional patients at risk for cardiovascular disease (whose eGFRs were also determined), we confirmed and extended the biomarkers associated with reduced filtration in this first CKD study (data not shown). These results provide further evidence for the validity of these potential CKD biomarkers, and further validation studies are in progress.

Overall, these results show that our multiplexed proteomics assay has the requisite reproducibility, sensitivity, and range for high-content proteomics studies and unbiased biomarker discovery.

We recognize that there are some limitations to the work presented here, which only demonstrates the specificity of SOMAmers for the proteins they were selected against. In order to further validate and standardize SOMAmer-based measurements, we plan studies with reference standards and other analytical methods, such as the affinity capture-MS method presented here. We are also expanding these studies to understand the specificity of SOMAmers for close homologues and alternate forms, such as the products of alternative splicing, post-translational modifications, and proteolytic cleavage. We believe that SOMAmers will be highly specific, given our previous experience with highly-specific aptamers including, for example, the drug Pegaptanib (Macugen) for the treatment age-related macular degeneration, which binds specifically to VEGF121 but not VEGF165 [37], [38], and an aptamer that distinguishes theophylline from caffeine, molecules that differ by just one methyl group [39].

In addition to the work described here, we have conducted clinical studies with our technology and discovered potential biomarkers in many areas with unmet medical need including cancer, cardiovascular conditions, neurological disorders, and infectious diseases. Frequently, the distributions of biomarker concentrations among two populations overlap to some degree, which creates the impetus to combine multiple biomarkers to achieve the most accurate diagnosis. In a related paper, we report the first large-scale application of this technology to discover and verify novel biomarkers for lung cancer in one of the most comprehensive proteomic biomarker studies published to date [40].

## Materials and Methods

### Ethics Statement

All studies of human subjects were conducted with written informed consent. Both the original study of CKD and the biomarker study reported here were approved by the Institutional Review Board at Weil Medical College of Cornell University.

### SOMAmer development and SELEX

Selection methods have been developed for use with poly-His-tagged, biotinylated, and non-tagged proteins. Many variations on these protocol have been used to select the >800 SOMAmers for the proteomic platform, such as alternating selection conditions to increase stringency for slow off-rate SOMAmers or performing the equilibrium steps in solution rather than with targets pre-immobilized. The following protocol is representative and was used for the selection described for the results shown in Table 1. Selection methods are further detailed in our patents and published patent applications [41], [42].

#### Preparation Modified Nucleotides

Modified nucleotides were manufactured by SomaLogic, Inc. with methods described by Vaught et al. [24].

#### Preparation of Candidate Mixtures

Candidate mixtures were prepared with dATP, dGTP, 5-methyl-dCTP (MedCTP) and either dTTP or one of three dUTP analogs: 5-benzylaminocarbonyl-dU (BndU), 5-tryptaminocarbonyl-dU  =  TrpdU, and 5-isobutylaminocarbonyl-dU (iBudU) (Figure 1). Candidate mixtures were prepared by polymerase extension of a primer annealed to a biotinylated template. Several enzymes were screened for the ability to incorporate these modified nucleotides, as well as to amplify a modified template. We used *Thermococcus kodakaraensis* (KOD) DNA polymerase for PCR with a slightly modified buffer, although at low efficiency. Additionally, conditions have been determined to amplify selected DNA using a two-step process to avoid potential amplification biases. For each candidate mixture composition, 4.8 nmol forward PCR primer and 4 nmol template were combined in 100 µL 1X KOD XL DNA Polymerase Buffer (EMD Chemicals), heated to 95°C for 8 minutes, and cooled on ice. Each 100 µL primer:template mixture was added to a 400 µL extension reaction containing 1X KOD DNA Polymerase Buffer, 0.125 U/µL KOD DNA Polymerase, and 0.5 mM each dATP, MedCTP, dGTP, and dTTP or dUTP analog, and incubated at 70°C for 30 minutes. Double-stranded product was captured via the template strand biotins by adding 1 mL streptavidin-coated magnetic beads (MagnaBind Streptavidin, Pierce, 5 mg/mL in 1 M NaCl +0.05% TWEEN-20) and incubating at 25°C for 10 minutes with mixing. Beads were washed three times with 0.75 mL SB1T Buffer (40 mM HEPES, pH 7.5, 125 mM NaCl, 5 mM KCl, 1 mM MgCl_2_, 1 mM CaCl_2_, 0.05% TWEEN-20). The SOMAmer strand was eluted from the beads with 1.2 mL 20 mM NaOH, neutralized with 0.3 mL 80 mM HCl, and buffered with 15 µL 1 M HEPES, pH 7.5. Candidate mixtures were concentrated with a Centricon-30 to approximately 0.2 mL, and quantified by UV absorbance spectroscopy.

#### Immobilization of Target Proteins

Target proteins were purchased with (His)_6_ tags from the following vendors: AnaSpec, APE-Bridgepath ARP, Athens Research and Technology, B-Bridge International, Inc, Biogenesis, Calzyme, EMD Biosciences, Enzyme Research Laboratories, Invitrogen, Millipore, Nexomics, Pepro Tech, Peptide Institute, Inc., ProSci, ProSpec, ProteinX Lab, Proteome Resources, LLC, Quality Biological, Quidel, R&D Systems, Research Diagnostics, RZPD GmbH, Sigma-Aldrich, United States Biological, Upstate Biotechnology, and VWR. Proteins were immobilized on Co^+2^-NTA paramagnetic beads (MyOne TALON beads, Invitrogen). Target proteins were diluted to 0.2 mg/mL in 0.5 mL B/W Buffer (50 mM Na-phosphate, pH 8.0, 300 mM NaCl, 0.01% TWEEN-20), and added to 0.5 mL MyOne TALON beads (pre-washed three times with B/W Buffer and resuspended to 10 mg/mL in B/W Buffer). The mixture was rotated for 30 minutes at 25°C and stored at 4°C until use. MyOne TALON beads coated with (His)_6_ peptide were also prepared and stored as above. Prior to use, beads were washed 3 times with B/W Buffer, once with SB1T, and resuspended in SB1T.

#### SOMAmer Selection

Affinity selections were performed separately with each candidate mixture, comparing binding between target protein beads (signal) and (His)_6_ beads (background). For each sample, a 0.5 µM candidate DNA mixture was prepared in 40 µL SB1T. 1 µL of 1 mM competitor oligo was added to the DNA, along with 10 µL of a protein competitor mixture (0.1% HSA, 10 µM casein, and 10 µM prothrombin in SB1T).

Binding reactions were performed by adding 50 µL target protein-coated beads or (His)_6_-coated beads (5 mg/mL in SB1T) to the DNA mixture and incubating at 37°C for 15 minutes with mixing. The DNA solution was removed and the beads were washed 5 times at 37°C with SB1T containing 0.1 mg/mL herring sperm DNA (Sigma Aldrich). Unless indicated, all washes were performed by resuspending the beads in 100 µL wash solution, mixing for 30 seconds, separating the beads with a magnet, and removing the wash solution. Bound SOMAmers were eluted from the beads by adding 100 µL SB1T +2 M Guanidine-HCl and incubating at 37°C, 5 minutes with mixing. The SOMAmer eluate was transferred to a new tube after magnetic separation. After the first two selection rounds, the final two of five target beads washes were done for 5 minutes instead of 30 seconds.

Primer beads were prepared by immobilizing biotinylated reverse PCR primer to streptavidin-coated paramagnetic beads (MyOne Streptavidin, Invitrogen). 5 mL MyOne Streptavidin beads (10 mg/mL) were washed once with NaClT (5 M NaCl, 0.01% TWEEN-20), and resuspended in 5 mL biotinylated reverse PCR primer (5 µM in NaClT). The sample was incubated at 25°C for 15 minutes, washed twice with 5 mL NaClT, resuspended in 12.5 mL NaClT (4 mg/mL), and stored at 4°C.

Twenty-five µL of primer beads (4 mg/mL in NaClT) were added to the 100 µL SOMAmer solution in Guanidine Buffer and incubated 50°C, 15 minutes with mixing. The SOMAmer solution was removed, and the beads were washed 5 times with SB1T. SOMAmer was eluted from the beads by adding 85 µL 20 mM NaOH and incubating at 37°C for 1 minute with mixing. 80 µL SOMAmer eluate was transferred to a new tube after magnetic separation, neutralized with 20 µL 80 mM HCl, and buffered with 1 µL 0.5 M Tris-HCl, pH 7.5.

#### SOMAmer Amplification and Purification

Selected SOMAmer DNA was amplified and quantified by QPCR. 48 µL DNA was added to 12 µL QPCR Mix (5X KOD DNA Polymerase Buffer, 25 mM MgCl_2_, 10 µM forward PCR primer, 10 µM biotinylated reverse PCR primer, 5X SYBR Green I, 0.125 U/µL KOD DNA Polymerase, and 1 mM each dATP, dCTP, dGTP, and dTTP) and thermal cycled in an ABI 5700 QPCR instrument (Applied Biosystems) with the following protocol: 1 cycle of 99.9°C, 15 seconds, 55°C, 10 seconds, 70°C, 30 minutes; 30 cycles of 99.9°C, 15 seconds, 72°C, 1 minute. Quantification was done with the instrument software and the number of copies of DNA selected with target beads and (His)_6_ beads were compared to determine signal/background ratios.

Following amplification, the PCR product was captured on MyOne Streptavidin beads via the biotinylated antisense strand. 1.25 mL MyOne Streptavidin beads (10 mg/mL) were washed twice with 0.5 mL 20 mM NaOH, once with 0.5 mL SB1T, resuspended in 2.5 mL 3 M NaCl, and stored at 4°C. 25 µL MyOne Streptavidin beads (4 mg/mL in 3 M NaCl) were added to 50 µL double-stranded QPCR product and incubated at 25°C for 5 minutes with mixing. The beads were washed once with SB1T, and the “sense” strand was eluted from the beads by adding 200 µL 20 mM NaOH and incubating at 37°C for 1 minute with mixing. The eluted strand was discarded and the beads were washed 3 times with SB1T and once with 16 mM NaCl.

SOMAmer sense strand was prepared with the appropriate nucleotide composition by primer extension from the immobilized antisense strand. The beads were resuspended in 20 µL primer extension reaction mix (1X KOD DNA Polymerase Buffer, 1.5 mM MgCl_2_, 5 µM forward PCR primer, 0.125 U/µL KOD DNA Polymerase, 0.5 mM each dATP, MedCTP, dGTP, and either dTTP or dUTP analog) and incubated at 68°C for 30 minutes with mixing. The beads were washed 3 times with SB1T, and the SOMAmer strand was eluted from the beads by adding 85 µL 20 mM NaOH and incubating at 37°C for 1 minute with mixing. 80 µL SOMAmer eluate was transferred to a new tube after magnetic separation, neutralized with 20 µL 80 mM HCl, and buffered with 5 µL 0.1 M HEPES, pH 7.5.

#### Selection Strategy and Feedback

The relative target protein concentration of the selection step was lowered each round in response to the S/B ratio as follows (Eq. 1):
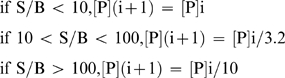
(1)


where [P]  =  protein concentration and i  =  current round number. Target protein concentration was lowered by adjusting the mass of target protein beads (and (His)_ 6_ beads) added to the selection step. After each selection round, the convergence state of the enriched DNA mixture was determined. 5 µL double-stranded QPCR product was diluted to 200 µL with 4 mM MgCl_2_ containing 1X SYBR Green I. Samples were overlaid with 75 µL silicone oil and analyzed for convergence as follows.

#### Nucleic Acid Reassociation Kinetics (C0t) Assay

The sample was thermal cycled with the following protocol: 3 cycles of 98°C, 1 minute, 85°C, 1 minute; 1 cycle of 93°C, 1 minute, 85°C, 15 minutes. During the 15 minutes at 85°C, fluorescent images were measured at 5-second intervals. The fluorescence intensity was plotted as a function of log (time) to evaluate the diversity of the sequences.

#### Measurement of Equilibrium Binding Constants

The equilibrium binding constants of the enriched libraries were measured using MyOne TALON bead partitioning. Radiolabled DNA was renatured by heating to 95°C for 3 minutes in SB1T and slowly cooling to 37°C. Complexes were formed by mixing a low concentration of DNA (∼1×10^−11^ M) with a range of concentrations of target protein (1×10^−7^ M to 1×10–12 M final) in SB1T and incubating at 37°C. One-twelfth of each reaction was transferred to a nylon membrane and dried to determine total counts in each reaction. 25 µg of MyOne TALON beads was added to the remainder of each reaction and mixed at 37°C for one minute. Two-thirds of the reaction was then passed through a MultiScreen HV Plate (Millipore) under vacuum to separate protein-bound complexes from unbound DNA and washed with 100 µL SB1T. The nylon membrane and MultiScreen HV Plates were phosphorimaged and the amount of radioactivity in each sample quantified using a FUJI FLA-3000 (Fujifilm Medical Systems). The fraction of captured DNA was plotted as a function of protein concentration and a non-linear curve-fitting algorithm was used to extract equilibrium binding constants (K_d_ values) from the data.

#### Measurement of Dissociation Rate Constants

The rate constant for SOMAmer:protein complex dissociation was determined for each aptamer by measuring the fraction of pre-formed aptamer:protein complexes that remain bound after addition of a competitor as a function of time. Radiolabled SOMAmer was renatured as described above. Approximately 5×10–11 M SOMAmer was equilibrated in SB18T (40 mM HEPES, 100 mM NaCl, 5 mM KCl, 5 mM MgCl_2_, 0.05% Tween-20 at pH 7.5) at 37°C with protein at a concentration 10X greater than the measured K_d_ value. Samples were then diluted 2X with 40 nM non-labeled SOMAmer or 0.3 mM dextran sulfate in SB18T at various time points. Complexes were partitioned to separate free aptamer from protein:aptamer complexes. The type of partitioning was dependent upon the protein used since not all proteins bind to the same type of partitioning resin. For LBP and Histone H1.2, Zorbax PSM-300A (Agilent Technologies) resin was used for partitioning; for Kallistatin, MyOne TALON beads were used; for biotinylated-TIG2, MyOne Streptavidin beads were used. Complexes were captured on the appropriate resin, and the sample was passed through a MultiScreen HV Plate under vacuum. The samples were washed with SB18T. The MultiScreen HV Plates were phosphorimaged and the amount of radioactivity in each sample quantified using a FUJI FLA-3000. The fraction of complex remaining was plotted as a function of time, and the dissociation rate constant was determined by fitting the data to an analytic expression for bimolecular dissociation kinetics using non-linear regression.

#### Affinity Capture Assay

Not all complexes are able to be captured in the “Catch-3” step of the affinity capture assay (Figure 5), and therefore analyzed by PAGE, because the 3′ end of the SOMAmer is sometimes involved in its structure or interaction with the target. Additional affinity capture examples for the subset of the CKD-related targets whose complexes can be captured on “Catch-3” beads are shown in Figure 5.

50% plasma samples were prepared by diluting ethylene diamine tetraacetic acid (EDTA)-plasma 2X in SB18T with 2 µM Z-Block_2 (the modified nucleotide sequence (AC-BnBn)7-AC). The plasma spike samples were prepared by diluting 500 ng protein with the 50% plasma in SB17T (SB18T with 1 mM EDTA) with 4-(2-Aminoethyl) benzenesulfonyl fluoride hydrochloride (AEBSF) and ethylene glycol tetraacetic acid (EGTA). The plasma samples were prepared by diluting the 50% plasma in SB17T with AEBSF and EGTA. The buffer spike samples were prepared by diluting 500 ng protein in SB17T with AEBSF and EGTA. These samples were combined with 10 pmoles of SOMAmer to give final concentrations of 10% plasma, 2 mM AEBSF, 0.5 mM EGTA, and 100 nM SOMAmer. Complexes were formed by incubating at 37°C for 45 minutes. 50 µL of a 20% slurry of Streptavidin agarose beads (ThermoFisher Scientific) was added to each sample and shaken for 10 minutes at room temperature. The samples were added to a MultiScreen HV Plate to perform washes under vacuum filtration. Each sample was washed one time quickly with 200 µL of SB17T, one time for one minute with 200 µL of 100 µM biotin in SB17T with shaking, one time with 200 µL of SB17T for one minute with shaking, and one time with 200 µL of SB17T for nine minutes with shaking. Proteins in the sample were labeled with both biotin and a fluorophore by incubating each sample in 100 µL of 1 mM EZ Link NHS-PEO4-biotin (Pierce), 0.25 mM NHS-Alexa-647 (Invitrogen) in SB17T for five minutes with shaking. Each sample was washed one time with 200 µL of 20 mM glycine in SB17T and five times with 200 µL of SB17T, shaking each wash for one minute. The final wash was removed using centrifugation at 1000 relative centrifugal force (RCF) for 30 seconds. The beads were resuspended with 100 µL of SB17T. SOMAmers (complexed and free) were released from the beads by exposure under a BlackRay light source (UVP XX-Series Bench Lamps, 365 nm) for ten minutes with shaking. The samples were spun out of the plate by centrifugation at 1000 RCF for 30 seconds. 10 µL of each sample was removed and reserved as “Catch-1 eluate” for SDS-PAGE analysis. The remainder of the samples was captured through the biotinylated proteins by adding 20 µL of a 20% slurry of monomeric Avidin beads and shaking for ten minutes. The beads were transferred to a MultiScreen HV Plate and washed four times with 100 µL of SB17T for one minute with shaking. The final wash was removed using centrifugation at 1000 RCF for 30 seconds. Proteins were eluted from the beads by incubating each sample with 100 µL of 2 mM biotin in SB17T for five minutes with shaking. Each eluate was transferred to 0.4 mg MyOne Streptavidin beads with a bound biotinylated-primer complementary to the 3′ fixed region of the SOMAmer. The samples were incubated for five minutes with shaking to anneal the bead-bound fixed region to the SOMAmer complexes. Each sample was washed two times with 100 µL of 1XSB17T for one minute each with shaking and one time with 100 µL of 1XSB19T (5 mM HEPES, 100 mM NaCl, 5 mM KCl, 5 mM MgCl_2_, 1 mM EDTA, 0.05% Tween-20, pH 7.5) for one minute with shaking, all by magnetic separation. The complexes were eluted by incubating with 45 µL of 20 mM NaOH for two minutes with shaking. 40 µL of each eluate was added to 10 µL of 80 mM HCl with 0.05% Tween-20 in a new plate. 10 µL of each sample was removed and reserved as “Catch-2 aptamer-bound eluate” for SDS-PAGE analysis. Gel samples were run on NuPAGE 4–12% Bis Tris Glycine gels (Invitrogen) under reducing and denaturing conditions according to the manufacturer's directions. Gels were imaged on an Alpha Innotech FluorChem Q scanner in the Cy5 channel to image the proteins.

### LC-MS/MS Protein Identification

Tandem mass spectra were collected at NextGen Sciences. Scaffold (Proteome Software) was used to probabilistically validate protein identifications derived from MS/MS sequencing results using the X!Tandem [43] ProteinProphet computer algorithms [44].

#### Database searching

All tandem MS/MS samples were analyzed using Mascot (Matrix Science; version Mascot). Mascot was set up to search the ipi.HUMAN.v3.53_plusREV database (147698 entries) assuming the digestion enzyme trypsin. Mascot was searched with a fragment ion mass tolerance of 0.50 Da and a parent ion tolerance of 5.0 PPM. Iodoacetamide derivative of cysteine was specified in Mascot as a fixed modification. S-carbamoylmethylcysteine cyclization (N-terminus) of the n-terminus, deamidation of asparagine, oxidation of methionine and acetylation of the n-terminus were specified in Mascot as variable modifications.

#### Criteria for protein identification

Scaffold (version Scaffold_2_04_01, Proteome Software Inc) was used to validate MS/MS based peptide and protein identifications. Peptide identifications were accepted if they could be established at greater than 50.0% probability as specified by the Peptide Prophet algorithm [45]. Protein identifications were accepted if they could be established at greater than 90.0% probability and contained at least 2 identified peptides. Protein probabilities were assigned by the Protein Prophet algorithm [44]. Proteins that contained similar peptides and could not be differentiated based on MS/MS analysis alone were grouped to satisfy the principles of parsimony.

### Proteomic Affinity Assay Method

All steps of the proteomic affinity assay were performed at room temperature unless otherwise indicated.

#### Sample thawing and plating

Aliquots of 100% serum or EDTA- plasma, stored at −80°C, were thawed by incubating in a 25°C water bath for ten minutes. After thawing the samples were stored on ice during mixing and prior to sample dilution. Samples were mixed by gentle vortexing (setting # 4 on Vortex Genie, Scientific Industries) for 8 seconds. A 20% sample solution was prepared by transferring 16 µL of thawed sample into 96-well plates (Hybaid Omnitube 0.3 mL, ThermoFisher Scientific) containing 64 µL per well of the appropriate sample diluent at 4°C. Sample diluent for serum was 0.8x SB17 with 0.6 mM MgCl_2_, 2 mM EGTA, 2 µM Z-Block_2, 0.05% Tween and for EDTA-plasma was 0.8x SB18 with 0.8 mM MgCl_2_, 2 mM EGTA, 2 µM Z-Block_2, 0.05% Tween. This plate was stored on ice until the next sample dilution steps were initiated.

#### Preparation of 10%, 1% and 0.03% SOMAmer Solutions

SOMAmers were grouped into three unique mixes. The placing of a SOMAmer within a mix was empirically determined by assaying a dilution series of serum or plasma with each SOMAmer and identifying the sample dilution that gave the largest linear range of signal. The segregation of SOMAmers and mixing with different dilutions of sample (10%, 1% or 0.03%) allow the assay to span a 10^7^-fold range of protein concentration. The composition of the custom SOMAmer mixes was slightly different between plasma and serum as expected due to variation in protein composition of these two media. The custom stock SOMAmer solutions for 10%, 1% and 0.03% serum and plasma were prepared and stored at 8x concentration in SB17T.

For each assay run, the three 8x SOMAmer solutions were diluted separately 1∶4 into SB17T to achieve 2x concentration. Each diluted SOMAmer master mix was heated to 95°C for five minutes and then to 37°C for 15 minutes. 55 µL of each 2x SOMAmer mix was manually pipetted into a 96-well plate resulting in three plates with 10%, 1% or 0.03% SOMAmer mixes. After mixing with sample, the final individual SOMAmer concentration ranged from 0.25–4 nM for serum, 0.5 nM for plasma.

#### Equilibration

A 2% sample plate was prepared by diluting the 20% sample 1∶10 into SB17T using the Beckman Coulter Biomek Fx^P^ (Beckman Coulter). A 0.06% sample plate was prepared by diluting the 2% sample plate 1∶31 into SB17T. The three sample dilutions were then transferred to their respective SOMAmer solutions by adding 55 µL of the sample to 55 µL of the appropriate 2x SOMAmer mix. The plates were sealed with a foil seal (Microseal ‘F’ Foil, Bio-Rad) and incubated at 37°C for 3.5 hours.

#### Preparation of Catch-1 Bead Plates

133.3 µL of a 7.5% Streptavidin-agarose bead slurry in SB17T was added to each well of three pre-washed 0.45 um filter plates. Each well of beads was washed once with 200 µL SB17T using vacuum filtration to remove the wash and then resuspended in 200 µL SB17T.

#### Catch-1 Bead Capture

All subsequent steps were performed by the Beckman Coulter Biomek Fx^P^ robot unless otherwise noted. After the 3.5 hour equilibration, 100 µL of the 10%, 1% and 0.03% equilibration binding reactions was transferred to their respective Catch-1 Streptavidin agarose filter plates and incubated with shaking for ten minutes. Unbound solution was removed via vacuum filtration. Each set of Catch-1 beads was washed with 190 µL of 100 µM biotin in SB17T and then 190 µL of SB17T using vacuum filtration to remove the wash. 190 µL SB17T was added to each well in the Catch-1 plates and incubated with shaking for ten minutes at 25°C. The wash was removed via vacuum filtration and the bottom of the filter plates blotted to remove droplets using the on-deck blot station.

#### Biotinylation of Proteins

An aliquot of 100 mM NHS-PEO4-biotin in DMSO was thawed at 37°C for six minutes and diluted to 1 mM with SB17T at pH 7.25. 100 µL of the NHS-PEO4-biotin was added to each well of each Catch-1 filter plate and incubated with shaking for five minutes. Each biotinylation reaction was quenched by adding 150 µL of 20 mM glycine in SB17T to the Catch-1 plates with the NHS-PEO4-biotin. Plates were incubated for one minute with shaking, vacuum filtrated, and 190 µL 20 mM glycine SB17T was added to each well in the plate. The plates were incubated for one minute, shaking before removal by vacuum filtration. 190 µL of SB17T was added to each well and removed by vacuum filtration. The wells of the Catch-1 plates were subsequently washed three times by adding 190 µL SB17T, incubating for one minute with shaking followed by vacuum filtration. After the last wash the plates were centrifuged at 1000 rpm for one minute over a 1 mL deep-well plate to remove extraneous volume before elution. Centrifugation was performed off deck.

#### Kinetic Challenge and Photo-Cleavage

85 µL of 10 mM dextran sulfate in SB17T was added to each well of the filter plates. The filter plates were placed onto a Thermal Shaker (Eppendorf) under a BlackRay light source and irradiated for ten minutes with shaking. The photo-cleaved solutions were sequentially eluted from each Catch-1 plate into a common deep well plate by centrifugation at 1000 rpm for one minute each.

#### Catch-2 Bead Capture

In bulk, MyOne-Streptavidin C1 beads were washed two times for 5 minutes each with equal volume of 20 mM NaOH and three times with an equal volume of SB17T. Beads were resuspended in SB17T to a concentration of 10 mg/mL. After resuspension, 50 µL of this solution was manually pipetted into each well of a 96-well plate and stored at 4°C until Catch-2. During Catch-2, the wash supernatant was removed via magnetic separation. All of the photo-cleaved eluate was pipetted onto the MyOne magnetic beads and incubated with shaking at 25°C for five minutes. The supernatant was removed from the MyOne beads via magnetic separation and 75 µL of SB17T was transferred to each well. The plate was mixed for one minute at 37°C with shaking and then 75 µL of 60% glycerol (in SB17T) at 37°C was transferred to each well. The plate was mixed for another minute at 37°C with shaking. The wash was removed via magnetic separation. These washes were repeated two more times. After removal of the third glycerol wash from the MyOne beads, 150 µL of SB17T was added to each well and the plates incubated at 37°C with shaking for one minute before removal by magnetic separation. The MyOne beads were washed a final time using 150 µL SB19T with incubation for one minute, prior to magnetic separation.

#### Catch-2 Bead Elution and Neutralization

SOMAmers were eluted from MyOne beads by incubating each well of beads with 105 µL of 100 mM CAPSO pH 10, 1 M NaCl, 0.05% Tween with shaking for five minutes. 90 µL of each eluate was transferred during magnetic separation to a new 96-well plate containing 10 µL of 500 mM HCl, 500 mM HEPES, 0.05% Tween-20, pH 7.5.

#### Hybridization

20 µL of each neutralized Catch-2 eluate was transferred to a new 96-well plate and 5 µL of 10x Agilent Block (Oligo aCGH/ChIP-on-chip Hybridization Kit, Large Volume, Agilent Technologies 5188–5380), containing a 10x spike of hybridization controls (10 Cy3 SOMAmers) was added to each well. After removing the plate from the robot, 25 µL of 2x Agilent Hybridization buffer (Oligo aCGH/ChIP-on-chip Hybridization Kit, Agilent Technologies) was manually pipetted to the each well of the plate containing the neutralized samples and blocking buffer. 40 µL of this solution was manually pipetted into each “well” of the hybridization gasket slide (Hybridization Gasket Slide - 8 microarrays per slide format, Agilent Technologies). Custom Agilent microarray slides containing 10 probes per array complementary to 40 nucleotide selected region of each SOMAmer with a 20x dT linker were placed onto the gasket slides according to the manufacturer's protocol. Each assembly (Hybridization Chamber Kit - SureHyb enabled, Agilent Technologies) was tightly clamped and loaded into a hybridization oven for 19 hours at 60°C rotating at 20 rpm.

#### Post-Hybridization Washing

Approximately 400 mL Wash Buffer 1 (Oligo aCGH/ChIP-on-chip Wash Buffer 1, Agilent Technologies) was placed into each of two separate glass staining dishes. Six of the twelve slide/gasket assemblies were sequentially disassembled into the first staining dish containing Wash Buffer 1. Once disassembled, the slide was quickly transferred into a slide rack in a second staining dish containing Wash Buffer 1. The slides were incubated for five minutes in Wash Buffer 1 with mixing via magnetic stir bar. The slide rack was then transferred to the 37°C Wash Buffer 2 (Oligo aCGH/ChIP-on-chip Wash Buffer 2, Agilent Technologies) and allowed to incubate for five minutes with stirring. The slide rack was transferred to a fourth staining dish containing acetonitrile and incubated for five minutes with stirring.

#### Microarray Imaging

The microarray slides were imaged with a microarray scanner (Agilent G2565CA Microarray Scanner System, Agilent Technologies) in the Cy3-channel at 5 µm resolution at 100% PMT setting and the XRD option enabled at 0.05. The resulting tiff images were processed using Agilent feature extraction software version 10.5.1.1 with the GE1_105_Dec08 protocol.

### Serum and Plasma Reproducibility Studies

For each plate, five aliquots of plasma or serum from 18 individuals were thawed and plated as described below. Six wells containing only buffer were run on every plate. Serum and plasma samples were run on separate plates because they require slightly different buffers as indicated above. Three plates of each sample type were run over the course of several days and included using different lots of buffers and other reagents that might be expected to change within a large study.

### Limits of Quantification (LOQ) Experiment

For the LOQ experiments, four different sets of protein mixes were prepared for each of the three SOMAmer mixes, 10%, 1% or 0.03%, for a total of 12 mixes and 356 proteins. The proteins for each mix were chosen to avoid combining known protein binding partners and known protease-substrate pairs. The proteins were diluted into SB17T containing 2 µM Z-Block_2 so that each protein was at a final concentration of 20 nM. The protein solutions were serially diluted 15.8-fold into SB17T for a total of six points (lowest concentration: 20.3 fM). All of the protein preparation was maintained on ice. Eight replicate protein titrations per set were pipetted into 96-well plates.

#### Precision profiles

The coefficient of variation (CV), the standard deviation (σ) of the calculated concentration divided by the concentration, is typically determined for computing LOQs. As analyte concentration approaches zero, the assay CV diverges. Similarly, for large analyte concentrations near the assay plateau, small changes in assay signal can give rise to large changes in calculated concentration, leading again to a divergence in CVs. In between these two divergences in CVs lies a concentration range for which the assay measurements have CVs of a desired limit or less.

We set this limit at 20% CV and determined the upper and lower LOQs as those high and low concentrations equal to 20% CV. Standard curves were computed by averaging the relative fluorescent units (RFUs) for eight replicate measurements at each concentration. A standard four parameter Hill model (Eq. 2) in log transformed RFU was used to fit the dose-response curves, where x denotes an analyte concentration. 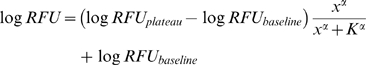
(2)


Two distinct approaches were used to compute precision profiles from these data. The first approach modeled the standard deviation for calculated concentrations *σ_x_*, obtained by averaging the eight replicates at each concentration, with a quadratic function from which the precision profile was directly obtained (Figure S3). The second approach is to model the standard deviation of the assay response *σ_logRFU_* with a quadratic function and then use the dose-response function to compute the variance in concentration from the response variance. This is not easily accomplished for the dose-response function used here but linearizing the function at a concentration *x* leads to the following simplification (Eq. 3 and 4).
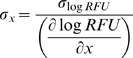
(3)

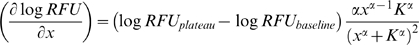
(4)


Typically, the assay CV in response units (*σ_logRFU_*/*logRFU*) is fairly constant so using a quadratic function to model *σ_RFU_* as a function of concentration should suffice.

We produced the full precision profile for each SOMAmer tested using both numerical approaches outlined above (Figure S3). Both methods give essentially the same result in this case for LLOQ and ULOQ. This particular analyte shows a remarkable five-log quantification range at a 20% CV cutoff with an LLOQ of 0.4–0.6 pM and a ULOQ of 40–50 nM. In general there is good agreement between the two different methods for computing precision profiles, and the assay response *σ_logRFU_* method was used to calculate the values reported in Table S2.

### Chronic Kidney Disease

CKD serum samples were collected by the Rogosin Institute for the clinical study entitled “Quantification of inflammatory and immune mediators of CKD in patient serum, whole blood and urine: Correlation with CKD disease stage progression”. Both the original study and the biomarker study reported here were approved by the Institutional Review Board at Weil Medical College of Cornell University. The clinical study design specified that samples be collected from 25 healthy controls with no renal disease and 25 subjects at each stage of CKD (1–5) for a total of 150 subjects. Our biomarker study included serum samples from 42 subjects that were available at the time this study was conducted. Table 3 summarizes the population demographics. The groups are well matched by gender, ethnicity, age, weight, and body mass index. Renal function, measured by the estimated glomerular filtration rate (eGFR, calculated with the MDRD formula for creatinine clearance [46]), is substantially different in the two groups (Table 3).

### Clinical Data Processing

#### Assay Normalization

Assay normalization was performed to reduce signal variation potentially introduced during the assay. Each sample in a study was normalized using a set of SOMAmers that have the lowest overall relative signal variation across all samples within a study. For each normalization SOMAmer, its median value was calculated from all samples in the study, and together these median values were used to calculate a scaling factor for each individual sample. The scaling factor was the mean of a series of values, one for each normalization SOMAmer, calculated as the sample signal divided by the median signal for the study. When applied to a sample, this procedure brings the signals corresponding to SOMAmers in the normalization set closer to the median values across the assay, and reduces the observed variation between replicate samples for all SOMAmers. Because the assay splits each clinical sample into three dilutions, assay normalization was performed separately on the three SOMAmer groups corresponding to the 10%, 1%, and 0.1% dilutions. Dilution normalization applies the same constant factor to every signal in that dilution from any given sample. This factor varied between samples in the range from 0.8 to 1.2, and was typically within 10% of unity.

#### Between-run Calibration

To compare samples between assay runs with slightly different conditions, we have applied calibration to the individual SOMAmer*s* signals. For this we apply a multiplicative correction factor specific to each SOMAmer, but invariant with respect to the sample (in contrast to normalization in which the factor was specific to the sample and did not vary from SOMAmer to SOMAmer within a sample). To calculate the calibration constant for each SOMAmer, we measure a set of eight calibrator samples derived from blood from the same individual in each sample set. From these calibrator sample measurements, we can standardize the signals from a sample within one run by applying the calibration coefficient for each SOMAmer that scales the median calibrator signal of that aptamer to a reference standard for that aptamer.

## Supporting Information

Figure S1 **Reproducibility of measurements in plasma and serum.** The cumulative distribution function (cdf) of intra‐run coefficients of variation (CVs) and inter‐run CVs for plasma and serum are shown for the three dilutions mixes: 10% (red), 1% (green), and 0.03% (blue).
(TIF)

Figure S2 **Precision profile for a2‐Antiplasmin.** **A.** Representative dose‐response curve calculated with a four‐parameter fit to average concentration (blue circles) of eight replicate protein measurements (red circles). **B.** Standard deviation σ_x_ of calculated concentration (blue circles) with quadratic fit (solid line) and 95% confidence (dashed lines). **C.** Standard deviation of assay response shown as σ_logRFU_ (red circles) with quadratic fit (solid line) and the 95% confidence (dashed lines). **D.** Precision profiles for assay response computed by modeling σ_x_ (blue) and σ_logRFU_ (red).
(TIF)

Figure S3 **Cumulative probability functions (cdfs) for limits of quantification computed from precision for 356 analytes measured in buffer.** A. Distribution of LLOQs; median 0.9 pM; inter‐quartile range 0.3 pM–3.9 pM; lowest 10 fM. B. Distribution of ULOQs; median 1.5 nM; inter‐quartile range 0.7 nM–4.5 nM. C. Distribution of log ROQ; median quantification range ∼3 logs.
(TIF)

Figure S4 **Comparison of LLOQ, ULOQ, and ROQ for 28 analytes measured in buffer and plasma.** All data were computed by modeling *σ_logRFU_*.
(TIF)

Table S1 List of the 813 proteins measured in the current version of the assay and the subset of 614 proteins measured in the CKD study.
(DOC)

Table S2 List of limits of quantification for 356 representative proteins measured in buffer.
(DOC)

Table S3 List of measured limits of quantification for proteins spiked into buffer and plasma.
(DOC)

Table S4 List of 60 proteins identified that varied between early and late stage CKD with a q‐value of 4.2×10^−4^.
(DOC)
