# Supplementary figures and images for: Aptamer-Based Multiplexed Proteomic Technology for Biomarker Discovery
Source: PLoS One. 2010 Dec 7;5(12):e15004. doi: 10.1371/journal.pone.0015004 (PMC3000457; doi:10.1371/journal.pone.0015004)

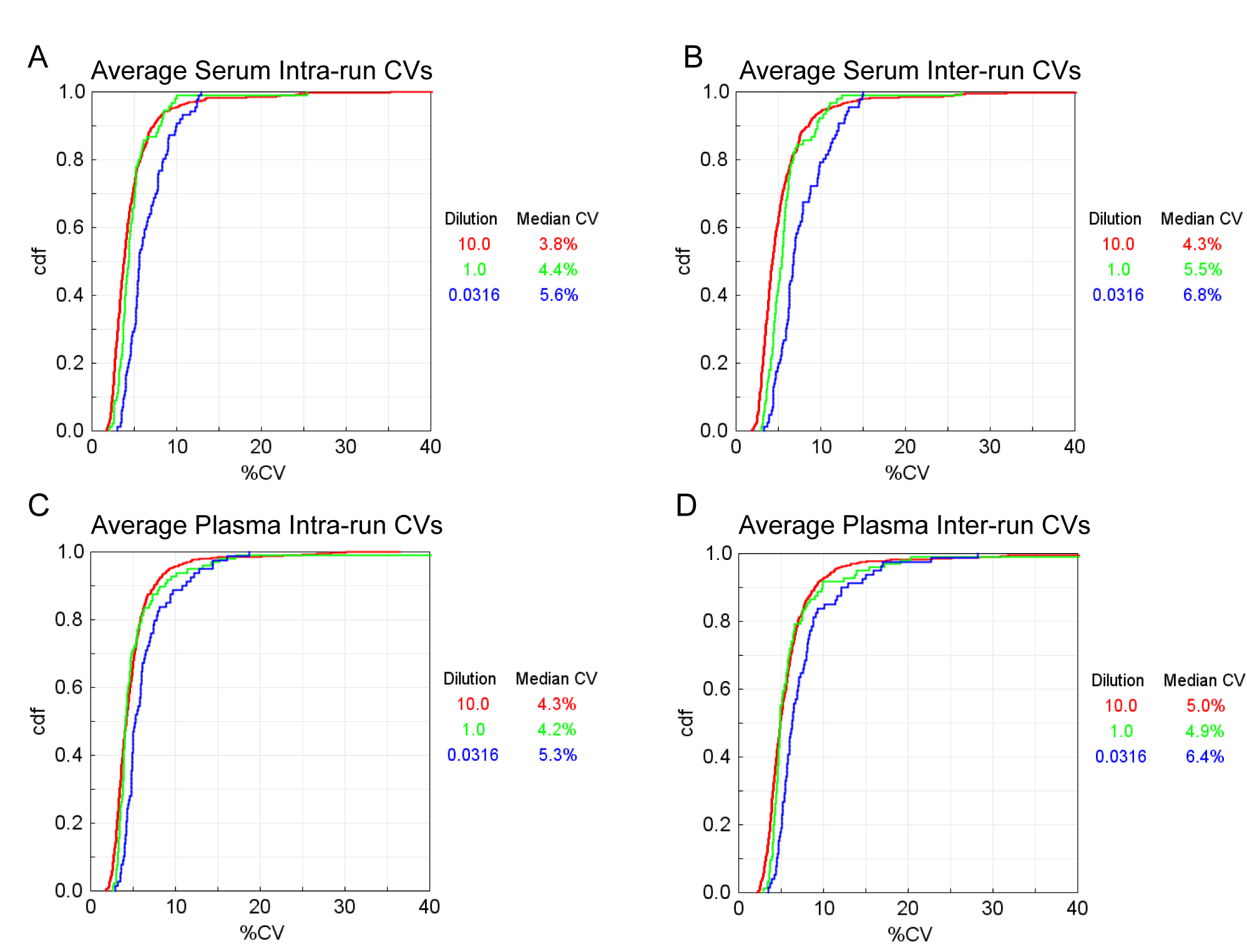

Supplement: Figure S1 — Reproducibility of measurements in plasma and serum. The cumulative distribution function (cdf) of intra‐run coefficients of variation (CVs) and inter‐run CVs for plasma and serum are shown for the three dilutions mixes: 10% (red), 1% (green), and 0.03% (blue). (TIF) [file pone.0015004.s001.tif]

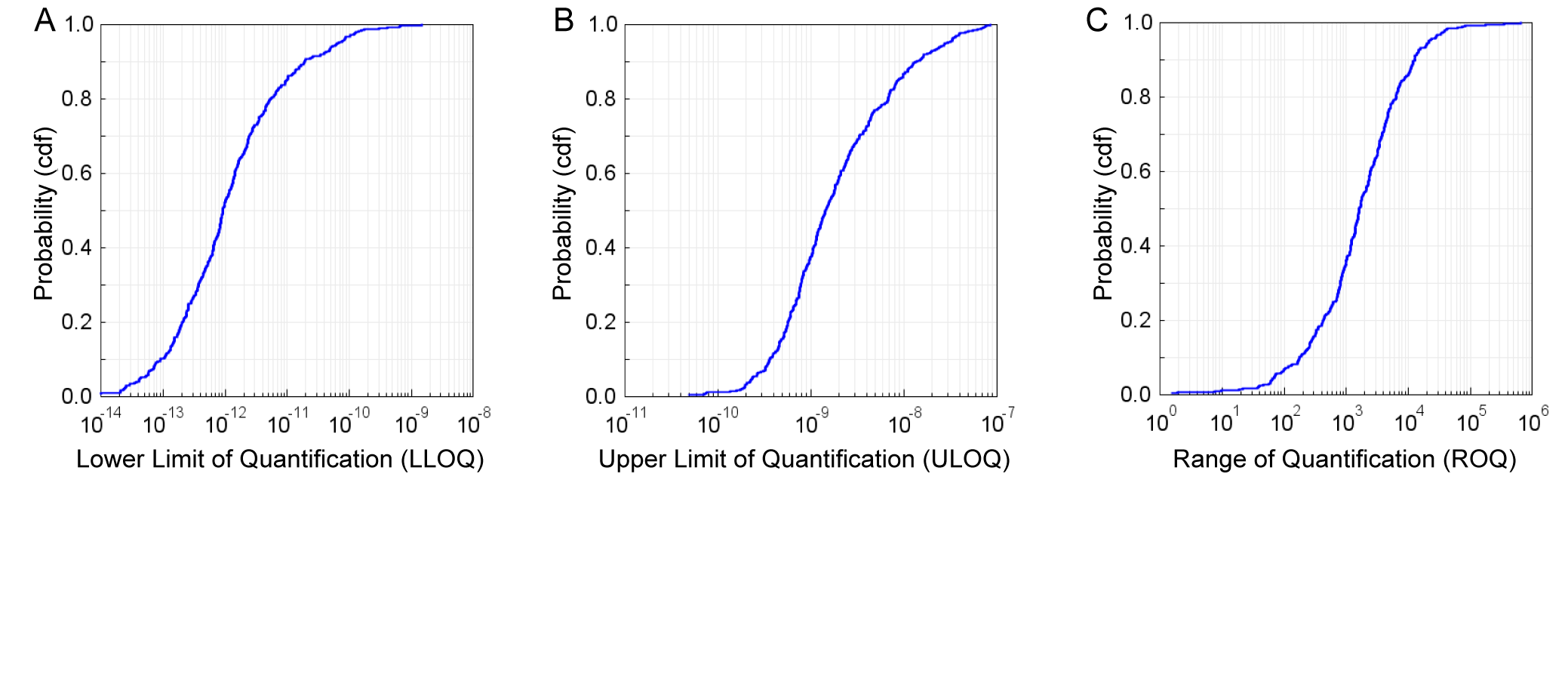

Supplement: Figure S2 — Precision profile for a2‐Antiplasmin. A. Representative dose‐response curve calculated with a four‐parameter fit to average concentration (blue circles) of eight replicate protein measurements (red circles). B. Standard deviation σx of calculated concentration (blue circles) with quadratic fit (solid line) and 95% confidence (dashed lines). C. Standard deviation of assay response shown as σlogRFU (red circles) with quadratic fit (solid line) and the 95% confidence (dashed lines). D. Precision profiles for assay response computed by modeling σx (blue) and σlogRFU (red). (TIF) [file pone.0015004.s002.tif]

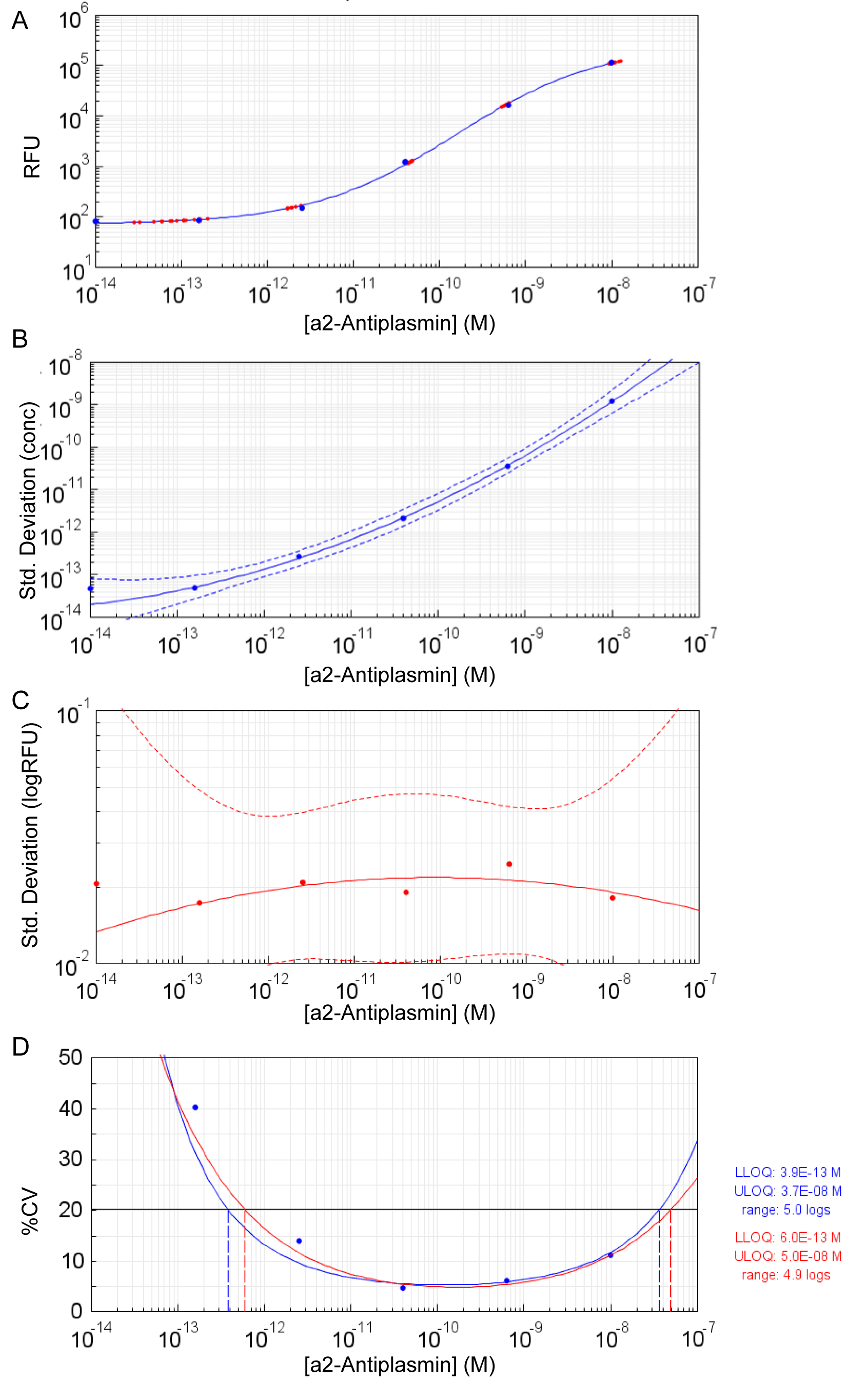

Supplement: Figure S3 — Cumulative probability functions (cdfs) for limits of quantification computed from precision for 356 analytes measured in buffer. A. Distribution of LLOQs; median 0.9 pM; inter‐quartile range 0.3 pM–3.9 pM; lowest 10 fM. B. Distribution of ULOQs; median 1.5 nM; inter‐quartile range 0.7 nM–4.5 nM. C. Distribution of log ROQ; median quantification range ∼3 logs. (TIF) [file pone.0015004.s003.tif]

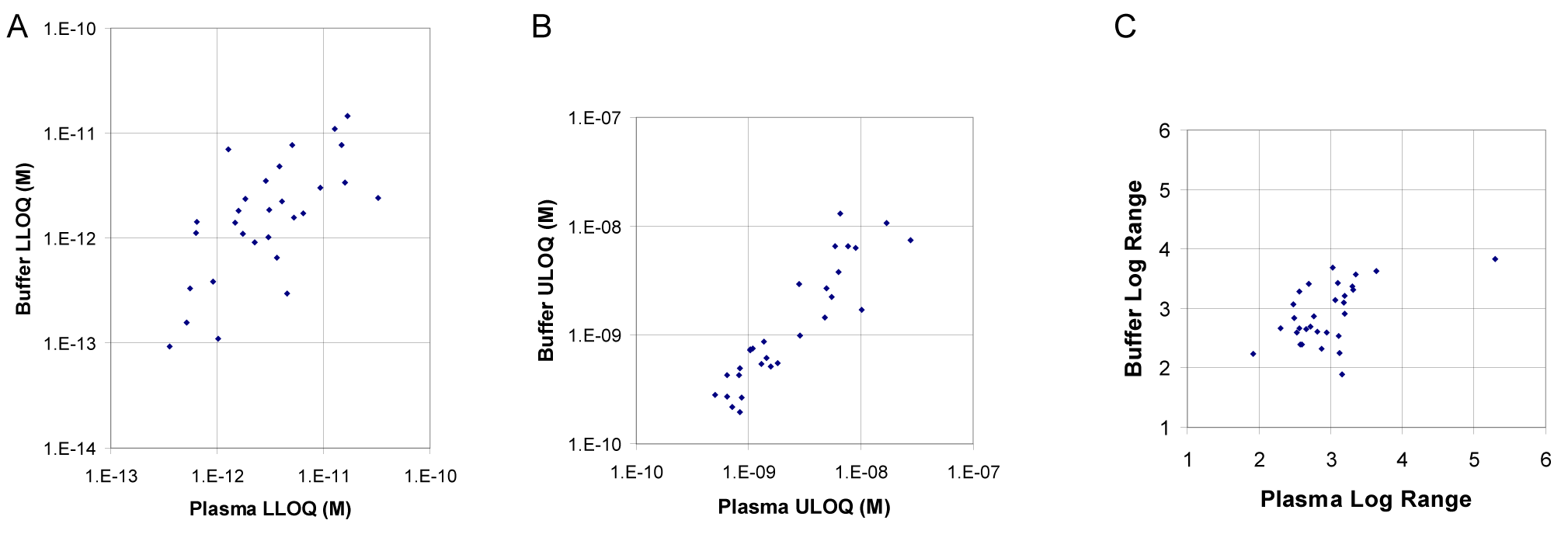

Supplement: Figure S4 — Comparison of LLOQ, ULOQ, and ROQ for 28 analytes measured in buffer and plasma. All data were computed by modeling σlogRFU. (TIF) [file pone.0015004.s004.tif]
