# Supplementary material for: Aptamer-Based Multiplexed Proteomic Technology for Biomarker Discovery
Source: PLoS One. 2010 Dec 7;5(12):e15004. doi: 10.1371/journal.pone.0015004 (PMC3000457; doi:10.1371/journal.pone.0015004)
Supplement: Table S1 — List of the 813 proteins measured in the current version of the assay and the subset of 614 proteins measured in the CKD study. (DOC) [file pone.0015004.s005.doc]

**Table S1. Current** **protein target menu.**

| Target Protein | Uni-Prot Protein Name | Uni-Prot Acc # | CKD^§^ |
| --- | --- | --- | --- |
| 14-3-3 protein zeta/delta | 14-3-3 protein zeta/delta | P63104 |  |
| 4-1BB | Tumor necrosis factor receptor superfamily member 9 | Q07011 | Y |
| 4-1BB ligand | Tumor necrosis factor ligand superfamily member 9 | P41273 |  |
| 6Ckine | Small-inducible cytokine A21 | O00585 | Y |
| α1-Antichymotrypsin | Alpha-1-antichymotrypsin | P01011 | Y |
| α1-Antitrypsin | Alpha-1-antitrypsin | P01009 |  |
| α2-Antiplasmin | Alpha-2-antiplasmin | P08697 | Y |
| α2-HS-Glycoprotein | Alpha-2-HS-glycoprotein | P02765 |  |
| α2-Macroglobulin | Alpha-2-macroglobulin | P01023 |  |
| ABL1 | Proto-oncogene tyrosine-protein kinase ABL1 | P00519 | Y |
| ABL2 | Tyrosine-protein kinase ABL2 | P42684 | Y |
| ACE-2 | Angiotensin-converting enzyme 2 | Q9BYF1 | Y |
| Activated Protein C | Vitamin K-dependent protein C (activated form) | P04070 | Y |
| Activin A | Inhibin beta A chain | P08476 | Y |
| Activin RIB | Activin receptor type-1B | P36896 | Y |
| ADAM 9 | Disintegrin and metalloproteinase domain-containing protein 9 | Q13443 |  |
| ADAMTS-1 | ADAMTS-1 | Q9UHI8 |  |
| ADAMTS-4 | ADAMTS-4 | O75173 | Y |
| ADAMTS-5 | ADAMTS-5 | Q9UNA0 | Y |
| ADAMTS-13 | ADAMTS-13 | Q76LX8 | Y |
| Adenosylhomocysteinase | Adenosylhomocysteinase | P23526 |  |
| Adiponectin | Adiponectin | Q15848 |  |
| aFGF | Heparin-binding growth factor 1 | P05230 | Y |
| AGGF1 | Angiogenic factor with G patch and FHA domains 1 | Q8N302 | Y |
| Aggrecan | Aggrecan core protein | P16112 | Y |
| AgRP | Agouti-related protein | O00253 | Y |
| AIF-1 | Allograft inflammatory factor 1 | P55008 | Y |
| AIP | AH receptor-interacting protein | O00170 |  |
| Albumin | Serum albumin | P02768 |  |
| ALCAM | Activated leukocyte cell adhesion molecule | Q13740 | Y |
| aldolase A | Fructose-bisphosphate aldolase A | P04075 |  |
| ALK-1 | Serine/threonine-protein kinase receptor R3 | P37023 | Y |
| Alkaline phosphatase, bone | Alkaline phosphatase, tissue-nonspecific isozyme | P05186 | Y |
| ALT | Alanine aminotransferase 1 | P24298 |  |
| Aminoacylase-1 | Aminoacylase-1 | Q03154 | Y |
| amphiregulin | Amphiregulin | P15514 | Y |
| amyloid precursor protein | Amyloid beta A4 protein | P05067 |  |
| Angiogenin | Angiogenin | P03950 | Y |
| Angiopoietin-1 | Angiopoietin-1 | Q15389 | Y |
| Angiopoietin-2 | Angiopoietin-2 | O15123 | Y |
| Angiopoietin-4 | Angiopoietin-4 | Q9Y264 | Y |
| Angiostatin | Angiostatin | P00747 |  |
| Angiotensinogen | Angiotensinogen | P01019 | Y |
| Angplt4 | Angiopoietin-related protein 4 | Q9BY76 |  |
| Angptl3 | Angiopoietin-related protein 3 | Q9Y5C1 | Y |
| Antithrombin III | Antithrombin-III | P01008 | Y |
| Apo A-I | Apolipoprotein A-I | P02647 | Y |
| Apo B | Apolipoprotein B-100 and Apolipoprotein B-48 | P04114 | Y |
| Apo E | Apolipoprotein E | P02649 | Y |
| Apo E2 | Apolipoprotein E (isoform E2) | P02649 | Y |
| Apo E3 | Apolipoprotein E (isoform E3) | P02649 | Y |
| Apo E4 | Apolipoprotein E (isoform E4) | P02649 | Y |
| ApoE R2 | Low-density lipoprotein receptor-related protein 8 | Q14114 | Y |
| APRIL | Tumor necrosis factor ligand superfamily member 13 | O75888 | Y |
| Arginase-1 | Arginase-1 | P05089 | Y |
| ARID3A | AT-rich interactive domain-containing protein 3A | Q99856 |  |
| ARSB | Arylsulfatase B | P15848 | Y |
| Artemin | Artemin | Q5T4W7 | Y |
| Arylsulfatase A | Arylsulfatase A | P15289 |  |
| ASAH2 | Neutral ceramidase | Q9NR71 | Y |
| ASAHL | N-acylethanolamine-hydrolyzing acid amidase | Q02083 |  |
| ASGPR1 | Asialoglycoprotein receptor 1 | P07306 |  |
| Aurora B | Serine/threonine-protein kinase 12 | Q96GD4 | Y |
| Aurora kinase A | Serine/threonine-protein kinase 6 | O14965 | Y |
| Azurocidin | Azurocidin | P20160 | Y |
| β2-Microglobulin | Beta-2-microglobulin | P61769 | Y |
| B7 | T-lymphocyte activation antigen CD80 | P33681 | Y |
| β-Endorphin | Beta-endorphin | P01189 | Y |
| β-NGF | Beta-nerve growth factor | P01138 | Y |
| BAFF | Tumor necrosis factor ligand superfamily member 13B | Q9Y275 | Y |
| BAFF Receptor | Tumor necrosis factor receptor superfamily member 13C | Q96RJ3 |  |
| BARK1 | Beta-adrenergic receptor kinase 1 | P25098 | Y |
| BCA-1 | Small-inducible cytokine B13 | O43927 |  |
| BCAM | Lutheran blood group glycoprotein | P50895 | Y |
| Bcl-2 | Apoptosis regulator Bcl-2 | P10415 | Y |
| BCL2A1 | Bcl-2-related protein A1 | Q16548 | Y |
| BCMA | Tumor necrosis factor receptor superfamily member 17 | Q02223 | Y |
| BDNF | Brain-derived neurotrophic factor | P23560 | Y |
| bFGF | Heparin-binding growth factor 2 | P09038 | Y |
| bFGF-R | Basic fibroblast growth factor receptor 1 | P11362 | Y |
| βIGH3 | Transforming growth factor-beta-induced protein ig-h3 | Q15582 | Y |
| Biglycan | Biglycan | P21810 | Y |
| BMP RII | Bone morphogenetic protein receptor type-2 | Q13873 | Y |
| BMP-1 | Bone morphogenetic protein 1 | P13497 | Y |
| BMP10 | Bone morphogenetic protein 10 | O95393 |  |
| BMP-14 | Growth/differentiation factor 5 | P43026 | Y |
| BMP-6 | Bone morphogenetic protein 6 | P22004 |  |
| BMP-7 | Bone morphogenetic protein 7 | P18075 | Y |
| BMPER | BMP-binding endothelial regulator protein | Q8N8U9 | Y |
| BMPR1A | Bone morphogenetic protein receptor type IA | P36894 |  |
| BMX | Cytoplasmic tyrosine-protein kinase BMX | P51813 | Y |
| BNP-32 | Brain natriuretic peptide 32 | P16860 |  |
| Bone proteoglycan II | Decorin | P07585 | Y |
| BPI | Bactericidal permeability-increasing protein | P17213 | Y |
| Brevican | Brevican core protein | Q96GW7 |  |
| BSP | Bone sialoprotein 2 | P21815 | Y |
| BTK | Tyrosine-protein kinase BTK | Q06187 | Y |
| C1q | Complement C1q subcomponent subunits A, B, and C | P02747,P02746,P02745 | Y |
| C1r | Complement C1r subcomponent | P00736 | Y |
| C1s | Complement C1s subcomponent | P09871 |  |
| C2 | Complement C2 | P06681 | Y |
| C3 | Complement C3 | P01024 | Y |
| C3a | C3a anaphylatoxin | P01024 | Y |
| C3adesArg | C3a anaphylatoxin des Arginine | P01024 | Y |
| C3b | Complement C3b | P01024 | Y |
| C3d | Complement C3d fragment | P01024 | Y |
| C4 | Complement C4-A and Complement C4-B | P0C0L4, P0C0L5 | Y |
| C4b | C4b-A | P0C0L4 P0C0L5 | Y |
| C5 | Complement C5 | P01031 | Y |
| C5a | C5a anaphylatoxin | P01031 | Y |
| C5b,6 Complex | Complement C5b, and Complement component C6 | P01031 P13671 | Y |
| C6 | Complement component C6 | P13671 | Y |
| C7 | Complement component C7 | P10643 | Y |
| C8 | Complement component C8 alpha, beta, and gamma chains | P07357,P07358,P07360 | Y |
| C9 | Complement component C9 | P02748 | Y |
| Cadherin-1 | Epithelial cadherin | P12830 | Y |
| Cadherin-2 | Neural cadherin | P19022 |  |
| Cadherin-5 | Vascular endothelial cadherin | P33151 | Y |
| Cadherin-6 | Kidney cadherin | P55285 |  |
| Cadherin-12 | Brain cadherin | P55289 | Y |
| Calcineurin B α | Calcineurin subunit B type 1 | P63098 |  |
| Calpain I | Calpain-1 catalytic subunit and Calpain small subunit 1 | P07384, P04632 | Y |
| Calpastatin | Calpastatin | P20810 | Y |
| CAMK1 | Calcium/calmodulin-dependent protein kinase type 1 | Q14012 |  |
| CAMK1D | Calcium/calmodulin-dependent protein kinase type 1D | Q8IU85 | Y |
| CAMK2A | Calcium/calmodulin-dependent protein kinase type II alpha chain | Q9UQM7 | Y |
| CAMK2B | Calcium/calmodulin-dependent protein kinase type II beta chain | Q13554 | Y |
| CAMK2D | Calcium/calmodulin-dependent protein kinase type II delta chain | Q13557 | Y |
| CaMKK α | Calcium/calmodulin-dependent protein kinase kinase 1 | Q8N5S9 |  |
| Carbonic anhydrase III | Carbonic anhydrase 3 | P07451 |  |
| Carbonic anhydrase IV | Carbonic anhydrase 4 | P22748 | Y |
| Carbonic anhydrase VI | Carbonic anhydrase 6 | P23280 | Y |
| Carbonic anhydrase VII | Carbonic anhydrase 7 | P43166 | Y |
| Carbonic anhydrase IX | Carbonic anhydrase 9 | Q16790 |  |
| Carbonic anhydrase XIII | Carbonic anhydrase 13 | Q8N1Q1 | Y |
| Carbonic anhydrase-related protein X | Carbonic anhydrase-related protein 10 | Q9NS85 | Y |
| Cardiotrophin-1 | Cardiotrophin-1 | Q16619 | Y |
| Carnosine dipeptidase 1 | Beta-Ala-His dipeptidase | Q96KN2 |  |
| Caspase-3 | Caspase-3 (pro form) | P42574 |  |
| Catalase | Catalase | P04040 | Y |
| Cathepsin A | Lysosomal protective protein | P10619 | Y |
| Cathepsin B | Cathepsin B | P07858 | Y |
| Cathepsin C | Dipeptidyl-peptidase 1 | P53634 |  |
| Cathepsin D | Cathepsin D | P07339 |  |
| Cathepsin E | Cathepsin E | P14091 |  |
| Cathepsin G | Cathepsin G | P08311 | Y |
| Cathepsin H | Cathepsin H | P09668 |  |
| Cathepsin S | Cathepsin S | P25774 | Y |
| Cathepsin V | Cathepsin L2 | O60911 | Y |
| CCL1 | Small-inducible cytokine A1 | P22362 | Y |
| CCL28 | Small-inducible cytokine A28 | Q9NRJ3 | Y |
| CD22 | B-cell receptor CD22 | P20273 | Y |
| CD23 | Low affinity immunoglobulin epsilon Fc receptor | P06734 | Y |
| CD30 | Tumor necrosis factor receptor superfamily member 8 | P28908 | Y |
| CD30 Ligand | Tumor necrosis factor ligand superfamily member 8 | P32971 | Y |
| CD36 ANTIGEN | Platelet glycoprotein 4 | P16671 | Y |
| CD39 | Ectonucleoside triphosphate diphosphohydrolase 1 | P49961 | Y |
| CD40 ligand, soluble | CD40 ligand | P29965 |  |
| CD48 | CD48 antigen | P09326 | Y |
| CD5L | CD5 antigen-like | O43866 | Y |
| CD70 | CD70 antigen | P32970 |  |
| CD97 | CD97 antigen | P48960 | Y |
| CD109 | CD109 antigen | Q6YHK3 |  |
| CDC37 | Hsp90 co-chaperone Cdc37 | Q16543 |  |
| CDK1/cyclin B | Cell division control protein 2 homolog, G2/mitotic-specific cyclin-B1 Complex | P06493, P14635 | Y |
| CDK2/cyclin A | Cell division protein kinase 2, Cyclin-A2 Complex | P24941, P20248 | Y |
| CDK5/p35 | Cell division protein kinase 5, Cyclin-dependent kinase 5 activator 1, p35 Complex | Q00535, Q15078 | Y |
| CDK8/cyclin C | Cell division protein kinase 8, Cyclin-C Complex | P49336, P24863 | Y |
| CEA | Carcinoembryonic antigen-related cell adhesion molecule 5 | P06731 |  |
| Chemerin | Retinoic acid receptor responder protein 2 | Q99969 | Y |
| Chitotriosidase-1 | Chitotriosidase-1 | Q13231 |  |
| Chk1 | Serine/threonine-protein kinase Chk1 | O14757 | Y |
| Chk2 | Serine/threonine-protein kinase Chk2 | O96017 | Y |
| CHL1 | Neural cell adhesion molecule L1-like protein | O00533 |  |
| Chordin-Like 1 | Chordin-like protein 1 | Q9BU40 | Y |
| Chymase | Chymase | P23946 | Y |
| CK-BB | Creatine kinase B-type | P12277 | Y |
| CK-MB | Creatine kinase B-type, Creatine kinase M-type | P12277, P06732 |  |
| CK-MM | Creatine kinase M-type | P06732 | Y |
| Ck-β-8-1 | Small-inducible cytokine A23 | P55773 | Y |
| CLF-1/CLC Complex | Cytokine receptor-like factor 1 and Cardiotrophin-like cytokine factor 1 | O75462, Q9UBD9 | Y |
| CN166 | UPF0568 protein C14orf166 | Q9Y224 |  |
| CNTF | Ciliary neurotrophic factor | P26441 | Y |
| CNTFR α | Ciliary neurotrophic factor receptor alpha | P26992 | Y |
| Coagulation Factor V | Coagulation factor V | P12259 | Y |
| Coagulation Factor VII | Coagulation factor VII | P08709 | Y |
| Coagulation Factor IX | Coagulation factor IX | P00740 | Y |
| Coagulation Factor IXab | Coagulation factor IX (activated form) | P00740 | Y |
| Coagulation Factor X | Coagulation factor X | P00742 | Y |
| Coagulation Factor Xa | Coagulation factor X (activated form) | P00742 | Y |
| Coagulation Factor XI | Coagulation factor XI | P03951 | Y |
| COLEC12 | Collectin-12 | Q5KU26 | Y |
| COMMD7 | COMM domain-containing protein 7 | Q86VX2 | Y |
| complement factor H-related 5 | Complement factor H-related protein 5 | Q9BXR6 |  |
| Contactin-1 | Contactin-1 | Q12860 | Y |
| Contactin-2 | Contactin-2 | Q02246 | Y |
| Contactin-4 | Contactin-4 | Q8IWV2 | Y |
| Contactin-5 | Contactin-5 | O94779 | Y |
| COX-2 | Prostaglandin G/H synthase 2 | P35354 | Y |
| Cripto | Teratocarcinoma-derived growth factor 1 | P13385 | Y |
| CRISP-3 | Cysteine-rich secretory protein 3 | P54108 | Y |
| CRP | C-reactive protein | P02741 |  |
| Cryptic | Cryptic protein | Q9GZR3 | Y |
| CSK | Tyrosine-protein kinase CSK | P41240 |  |
| CSK21 | Casein kinase II subunit alpha | P68400 | Y |
| CTACK | Small-inducible cytokine A27 | Q9Y4X3 | Y |
| CTGF | Connective tissue growth factor | P29279 | Y |
| CTLA-4 | Cytotoxic T-lymphocyte protein 4 | P16410 | Y |
| CXCL16, soluble | Small-inducible cytokine B16 | Q9H2A7 | Y |
| Cyclophilin A | Peptidyl-prolyl cis-trans isomerase A | P62937 |  |
| Cystatin C | Cystatin-C | P01034 | Y |
| Cystatin D | Cystatin-D | P28325 |  |
| Cystatin F | Cystatin-F | O76096 | Y |
| Cystatin M | Cystatin-M | Q15828 | Y |
| Cystatin S | Cystatin-S | P01036 |  |
| Cystatin SN | Cystatin-SN | P01037 | Y |
| Cytochrome c | Cytochrome c | P99999 | Y |
| Cytochrome P450 3A4 | Cytochrome P450 3A4 | P08684 | Y |
| DAN | Neuroblastoma suppressor of tumorigenicity 1 | P41271 | Y |
| DAPK2 | Death-associated protein kinase 2 | Q9UIK4 |  |
| DARPP-32 | Protein phosphatase 1 regulatory subunit 1B | Q9UD71 | Y |
| DC-SIGN | CD209 antigen | Q9NNX6 | Y |
| DC-SIGNR | C-type lectin domain family 4 member M | Q9H2X3 | Y |
| DEAD-box protein 19B | ATP-dependent RNA helicase DDX19B | Q9UMR2 | Y |
| Dectin-1 | C-type lectin domain family 7 member A | Q9BXN2 |  |
| Desmoglein-1 | Desmoglein-1 | Q02413 | Y |
| Discoidin domain receptor 1 | Epithelial discoidin domain-containing receptor 1 | Q08345 |  |
| Discoidin domain receptor 2 | Discoidin domain-containing receptor 2 | Q16832 |  |
| Dkk-1 | Dickkopf-related protein 1 | O94907 | Y |
| Dkk-3 | Dickkopf-related protein 3 | Q9UBP4 |  |
| Dkk-4 | Dickkopf-related protein 4 | Q9UBT3 | Y |
| DLC8 | Dynein light chain 1, cytoplasmic | P63167 |  |
| DLL4 | Delta-like protein 4 | Q9NR61 | Y |
| DLRB1 | Dynein light chain roadblock-type 1 | Q9NP97 |  |
| DMP1 | Dentin matrix acidic phosphoprotein 1 | Q13316 |  |
| dopa decarboxylase | Aromatic-L-amino-acid decarboxylase | P20711 | Y |
| DPP2 | Dipeptidyl-peptidase 2 | Q9UHL4 |  |
| DRG-1 | Vacuolar protein sorting-associated protein VTA1 homolog | Q9NP79 | Y |
| DRR1 | Protein FAM107A | O95990 | Y |
| Dtk | Tyrosine-protein kinase receptor TYRO3 | Q06418 | Y |
| DYRK3 | Dual specificity tyrosine-phosphorylation-regulated kinase 3 | O43781 |  |
| ECM1 | Extracellular matrix protein 1 | Q16610 | Y |
| EDA | Ectodysplasin-A (splice variant A2) | Q92838 | Y |
| EDAR | Tumor necrosis factor receptor superfamily member EDAR | Q9UNE0 | Y |
| EF-1-β | Elongation factor 1-beta | P24534 |  |
| EF-1-γ | Elongation factor 1-gamma | P26641 |  |
| EG-VEGF | Prokineticin-1 | P58294 | Y |
| eIF-4H | Eukaryotic translation initiation factor 4H | Q15056 |  |
| eIF-5 | Eukaryotic translation initiation factor 5 | P55010 | Y |
| eIF-5A-1 | Eukaryotic translation initiation factor 5A-1 | P63241 |  |
| Elastase | Leukocyte elastase | P08246 | Y |
| EMAP-2 | Endothelial monocyte-activating polypeptide 2 | Q12904 | Y |
| EMMPRIN | Basigin | P35613 |  |
| ENA-78 | C-X-C motif chemokine 5 | P42830 | Y |
| Endocan | cDNA FLJ50870, moderately similar to Endothelial cell-specific molecule 1 | Q3V4E3 |  |
| Endostatin | Endostatin | P39060 | Y |
| Endothelin-converting enzyme 1 | Endothelin-converting enzyme 1 | P42892 |  |
| Enterokinase | Enteropeptidase | P98073 | Y |
| Eotaxin | Eotaxin | P51671 | Y |
| Eotaxin-2 | Small-inducible cytokine A24 | O00175 | Y |
| EphA1 | Ephrin type-A receptor 1 | P21709 | Y |
| EPHA3 | Ephrin type-A receptor 3 | P29320 | Y |
| EphA5 | Ephrin type-A receptor 5 | P54756 |  |
| EphB4 | Ephrin type-B receptor 4 | P54760 |  |
| Ephrin-A4 | Ephrin-A4 | P52798 | Y |
| Ephrin-A5 | Ephrin-A5 | P52803 | Y |
| Ephrin-B3 | Ephrin-B3 | Q15768 | Y |
| Epithelial cell kinase | Ephrin type-A receptor 2 | P29317 | Y |
| EPO-R | Erythropoietin receptor | P19235 | Y |
| ER | Estrogen receptor | P03372 | Y |
| ERBB1 | Epidermal growth factor receptor | P00533 | Y |
| ERBB2 | Receptor tyrosine-protein kinase erbB-2 | P04626 | Y |
| ERBB3 | Receptor tyrosine-protein kinase erbB-3 | P21860 | Y |
| ERBB4 | Receptor tyrosine-protein kinase erbB-4 | Q15303 | Y |
| Erythropoietin | Erythropoietin | P01588 | Y |
| ESAM | Endothelial cell-selective adhesion molecule | Q96AP7 | Y |
| ETHE1 | Protein ETHE1, mitochondrial | O95571 |  |
| Factor B | Complement factor B | P00751 | Y |
| Factor D | Complement factor D | P00746 | Y |
| Factor H | Complement factor H | P08603 | Y |
| Factor I | Complement factor I | P05156 | Y |
| Fas ligand, soluble | Tumor necrosis factor ligand superfamily member 6 | P48023 | Y |
| FCγ2A | Low affinity immunoglobulin gamma Fc region receptor II-a | P12318 | Y |
| FCγ2B | Low affinity immunoglobulin gamma Fc region receptor II-b | P31994 | Y |
| FCγ3B | Low affinity immunoglobulin gamma Fc region receptor III-B | O75015 |  |
| FCγR1 | High affinity immunoglobulin gamma Fc receptor I | P12314 | Y |
| Ferritin | Ferritin heavy and light chains | P02794, P02792 | Y |
| Fetuin B | Fetuin-B | Q9UGM5 | Y |
| FGF-4 | Fibroblast growth factor 4 | P08620 |  |
| FGF-5 | Fibroblast growth factor 5 | P12034 | Y |
| FGF-6 | Fibroblast growth factor 6 | P10767 | Y |
| FGF-7 | Keratinocyte growth factor | P21781 | Y |
| FGF-8B | Fibroblast growth factor 8 - isoform 8B | P55075 | Y |
| FGF-9 | Glia-activating factor | P31371 | Y |
| FGF-10 | Fibroblast growth factor 10 | O15520 | Y |
| FGF-16 | Fibroblast growth factor 16 | O43320 | Y |
| FGF-17 | Fibroblast growth factor 17 | O60258 | Y |
| FGF-18 | Fibroblast growth factor 18 | O76093 | Y |
| FGF-19 | Fibroblast growth factor 19 | O95750 | Y |
| FGF-20 | Fibroblast growth factor 20 | Q9NP95 | Y |
| FGF-23 | Fibroblast growth factor 23 | Q9GZV9 | Y |
| FGFR-2 | Fibroblast growth factor receptor 2 | P21802 |  |
| FGFR-3 | Fibroblast growth factor receptor 3 | P22607 |  |
| FGR | Proto-oncogene tyrosine-protein kinase FGR | P09769 |  |
| Fibrinogen | Fibrinogen alpha, beta, and gamma chains | P02671, P02675, P02679 | Y |
| Fibronectin | Fibronectin | P02751 | Y |
| Fibronectin FN1.3 | Fibronectin-1 Fragment 3 | P02751 | Y |
| Fibronectin FN1.4 | Fibronectin-1 Fragment 4 | P02751 | Y |
| Ficolin-1 | Ficolin-1 | O00602 |  |
| Ficolin-2 | Ficolin-2 | Q15485 | Y |
| Ficolin-3 | Ficolin-3 | O75636 |  |
| Flt-3 | FL cytokine receptor | P36888 | Y |
| Flt-3 ligand | SL cytokine | P49771 | Y |
| Follistatin | Follistatin | P19883 | Y |
| Fortilin | Translationally-controlled tumor protein | P13693 |  |
| Fractalkine/CX3CL-1 | Fractalkine | P78423 | Y |
| FRP-1, soluble | Secreted frizzled-related protein 1 | Q8N474 | Y |
| FRP-3, soluble | Secreted frizzled-related protein 3 | Q92765 | Y |
| FSH | Glycoprotein hormones alpha chain and Follitropin subunit beta | P01215, P01225 | Y |
| FSTL3 | Follistatin-related protein 3 | O95633 | Y |
| FYN | Proto-oncogene tyrosine-protein kinase Fyn | P06241 | Y |
| GA733-1 protein | Tumor-associated calcium signal transducer 2 | P09758 | Y |
| Galectin-2 | Galectin-2 | P05162 | Y |
| Galectin-3 | Galectin-3 | P17931 | Y |
| Galectin-4 | Galectin-4 | P56470 | Y |
| GAPDH | Glyceraldehyde-3-phosphate dehydrogenase | P04406 |  |
| Gas1 | Growth arrest-specific protein 1 | P54826 | Y |
| GASP-1 | WAP, kazal, immunoglobulin, kunitz and NTR domain-containing protein 2 | Q8TEU8 | Y |
| GASP-2 | G-protein coupled receptor-associated sorting protein 2 | Q96D09 | Y |
| Gastrin-releasing peptide | Gastrin-releasing peptide | P07492 |  |
| GCP-2 | C-X-C motif chemokine 6 | P80162 | Y |
| G-CSF-R | Granulocyte colony-stimulating factor receptor | Q99062 | Y |
| GDF-11 | Growth/differentiation factor 11 | O95390 | Y |
| GDF-9 | Growth/differentiation factor 9 | O60383 | Y |
| GDNF | Glial cell line-derived neurotrophic factor | P39905 | Y |
| GFAP | Glial fibrillary acidic protein | P14136 | Y |
| GFR α-1 | GDNF family receptor alpha-1 | P56159 | Y |
| GFR α-2 | GDNF family receptor alpha-2 | O00451 | Y |
| GFR α-3 | GDNF family receptor alpha-3 | O60609 | Y |
| Glucocorticoid receptor | Glucocorticoid receptor | P04150 | Y |
| Glutamate carboxypeptidase | Cytosolic non-specific dipeptidase | Q96KP4 | Y |
| Glycoprotein VI | Platelet glycoprotein VI | Q9HCN6 | Y |
| Glypican 2 | Glypican-2 | Q8N158 | Y |
| Glypican 3 | Glypican-3 | P51654 | Y |
| GNS | N-acetylglucosamine-6-sulfatase | P15586 |  |
| gp130, soluble | Interleukin-6 receptor subunit beta | P40189 | Y |
| GPIIbIIIa | Integrin alpha-Iib and Integrin beta-3 | P08514,P05106 |  |
| Granulysin | Granulysin | P22749 |  |
| Granzyme A | Granzyme A | P12544 | Y |
| Granzyme B | Granzyme B | P10144 | Y |
| Granzyme H | Granzyme H | P20718 | Y |
| Group IB phospholipase A2 | Phospholipase A2 | P04054 | Y |
| Group IIA phospholipase A2 | Phospholipase A2, membrane associated | P14555 | Y |
| Group IIE phospholipase A2 | Group IIE secretory phospholipase A2 | Q9NZK7 | Y |
| Group V phospholipase A2 | Calcium-dependent phospholipase A2 | P39877 | Y |
| Group X phospholipase A2 | Group 10 secretory phospholipase A2 | O15496 | Y |
| Growth hormone receptor | Growth hormone receptor | P10912 | Y |
| Gro-α | Growth-regulated alpha protein | P09341 | Y |
| Gro-β | Macrophage inflammatory protein 2-alpha | P19875 |  |
| Gro-γ | Macrophage inflammatory protein 2-beta | P19876 | Y |
| GSK-3 α | Glycogen synthase kinase-3 alpha | P49840 | Y |
| GSK-3 β | Glycogen synthase kinase-3 beta | P49841 | Y |
| HAI-1 | Kunitz-type protease inhibitor 1 | O43278 | Y |
| HAI-2 | Kunitz-type protease inhibitor 2 | O43291 |  |
| HAPLN1 | Hyaluronan and proteoglycan link protein 1 | P10915 | Y |
| Haptoglobin, Mixed Type | Haptoglobin | P00738 | Y |
| Hat1 | Histone acetyltransferase type B catalytic subunit | O14929 | Y |
| HB-EGF | Heparin-binding EGF-like growth factor | Q99075 | Y |
| HCC-1 | C-C motif chemokine 14 | Q16627 | Y |
| HCC-4 | Small-inducible cytokine A16 | O15467 | Y |
| HCK | Tyrosine-protein kinase HCK | P08631 | Y |
| HDAC8 | Histone deacetylase 8 | Q9BY41 | Y |
| HE4 | WAP four-disulfide core domain protein 2 | Q14508 | Y |
| Heme oxygenase 2 | Heme oxygenase 2 | P30519 | Y |
| Hemopexin | Hemopexin | P02790 | Y |
| Heparin cofactor II | Heparin cofactor 2 | P05546 | Y |
| Hepcidin-25 | Hepcidin | P81172 | Y |
| HGF | Hepatocyte growth factor | P14210 | Y |
| HGF activator | Hepatocyte growth factor activator | Q04756 |  |
| HGF R | Hepatocyte growth factor receptor | P08581 | Y |
| HINT1 | Histidine triad nucleotide-binding protein 1 | P49773 |  |
| HIPK3 | Homeodomain-interacting protein kinase 3 | Q9H422 | Y |
| Histone H1.2 | Histone H1.2 | P16403 | Y |
| Histone H2A.z | Histone H2A.Z | P0C0S5 |  |
| HIV-2 Rev | Protein Rev (Human Immunodeficiency Virus) | P18093 | Y |
| HMG-1 | High mobility group protein B1 | P09429 | Y |
| HMTase G9a | Histone-lysine N-methyltransferase, H3 lysine-9 specific 3 | Q96KQ7 | Y |
| HPV E7 Type 16 | Protein E7 (Human Papillomavirus Type 16) | P03129 | Y |
| HPV E7 Type 18 | Protein E7 (Human Papillomavirus Type 18) | P06788 | Y |
| HSP 40 | DnaJ homolog subfamily B member 1 | P25685 |  |
| HSP 60 | 60 kDa heat shock protein, mitochondrial | P10809 | Y |
| HSP 70 | Heat shock 70 kDa protein 1 | P08107 |  |
| HSP 90α | Heat shock protein HSP 90-alpha | P07900 | Y |
| HSP 90β | Heat shock protein HSP 90-beta | P08238 | Y |
| HTRA2 | Serine protease HTRA2, mitochondrial | O43464 | Y |
| iC3b | Complement C3b, incactivated | P01024 | Y |
| ICOS | Inducible T-cell co-stimulator | Q9Y6W8 | Y |
| IDUA | Alpha-L-iduronidase | P35475 | Y |
| Iduronate 2-sulfatase | Iduronate 2-sulfatase | P22304 | Y |
| IFN-αA | Interferon alpha-2 | P01563 | Y |
| IFN-γ | Interferon gamma | P01579 | Y |
| IFN-γ R1 | Interferon-gamma receptor alpha chain | P15260 |  |
| IFN-λ 1 | Interleukin-29 | Q8IU54 | Y |
| IFN-λ 2 | Interleukin-28A | Q8IZJ0 | Y |
| IgE | Immunoglobulin E |  | Y |
| IGFBP-1 | Insulin-like growth factor-binding protein 1 | P08833 | Y |
| IGFBP-2 | Insulin-like growth factor-binding protein 2 | P18065 | Y |
| IGFBP-3 | Insulin-like growth factor-binding protein 3 | P17936 | Y |
| IGFBP-4 | Insulin-like growth factor-binding protein 4 | P22692 | Y |
| IGFBP-5 | Insulin-like growth factor-binding protein 5 | P24593 | Y |
| IGFBP-6 | Insulin-like growth factor-binding protein 6 | P24592 | Y |
| IGFBP-7 | Insulin-like growth factor-binding protein 7 | Q16270 | Y |
| IGF-I | Insulin-like growth factor IA and IB | P01343, P05019 | Y |
| IGF-II receptor | Cation-independent mannose-6-phosphate receptor | P11717 | Y |
| IgG | Immunoglobulin G |  |  |
| IgM | Immunoglobulin M |  | Y |
| IL-1β | Interleukin-1 beta | P01584 | Y |
| IL-1 F7 | Interleukin-1 family member 7 | Q9NZH6 | Y |
| IL-1 R AcP | Interleukin-1 receptor accessory protein | Q9NPH3 | Y |
| IL-1 R4 | Interleukin-1 receptor-like 1 | Q01638 |  |
| IL-1 Rrp2 | Interleukin-1 receptor-like 2 | Q9HB29 | Y |
| IL-1 sRI | Interleukin-1 receptor type I | P14778 | Y |
| IL-2 | Interleukin-2 | P60568 | Y |
| IL-2 sRα | Interleukin-2 receptor alpha chain | P01589 |  |
| IL-2 sRγ | Cytokine receptor common gamma chain | P31785 | Y |
| IL-4 | Interleukin-4 | P05112 | Y |
| IL-4 sR | Interleukin-4 receptor alpha chain | P24394 | Y |
| IL-5 | Interleukin-5 | P05113 |  |
| IL-6 | Interleukin-6 | P05231 | Y |
| IL-6 sRα | Interleukin-6 receptor alpha chain | P08887 | Y |
| IL-7 | Interleukin-7 | P13232 | Y |
| IL-7 Rα | Interleukin-7 receptor alpha chain | P16871 | Y |
| IL-8 | Interleukin-8 | P10145 | Y |
| IL-9 | Interleukin-9 | P15248 | Y |
| IL-10 | Interleukin-10 | P22301 | Y |
| IL-10 Rβ | Interleukin-10 receptor beta chain | Q08334 | Y |
| IL-11 | Interleukin-11 | P20809 | Y |
| IL-11 Rα | Interleukin-11 receptor alpha chain | Q14626 |  |
| IL-12 | Interleukin-12 subunits alpha and beta | P29459, P29460 |  |
| IL-12 Rβ1 | Interleukin-12 receptor beta-1 chain | P42701 | Y |
| IL-12 Rβ2 | Interleukin-12 receptor beta-2 chain | Q99665 | Y |
| IL-13 | Interleukin-13 | P35225 | Y |
| IL-13 Rα1 | Interleukin-13 receptor alpha-1 chain | P78552 | Y |
| IL-15 Rα | Interleukin-15 receptor alpha chain | Q13261 | Y |
| IL-16 | Interleukin-16 | Q14005 | Y |
| IL-17 | Interleukin-17A | Q16552 | Y |
| IL-17B | Interleukin-17B | Q9UHF5 | Y |
| IL-17D | Interleukin-17D | Q8TAD2 | Y |
| IL-17E | Interleukin-25 | Q9H293 | Y |
| IL-17F | Interleukin-17F | Q96PD4 | Y |
| IL-17 RC | Interleukin-17 receptor C | Q8NAC3 |  |
| IL-17 RD | Interleukin-17 receptor D | Q8NFM7 | Y |
| IL-17 sR | Interleukin-17 receptor A | Q96F46 | Y |
| IL-18 BPa | Interleukin-18-binding protein | O95998 | Y |
| IL-18 Rα | Interleukin-18 receptor 1 | Q13478 | Y |
| IL-18 Rβ | Interleukin-18 receptor accessory protein | O95256 | Y |
| IL-19 | Interleukin-19 | Q9UHD0 | Y |
| IL-20 | Interleukin-20 | Q9NYY1 | Y |
| IL-22 | Interleukin-22 | Q9GZX6 | Y |
| IL-22 Rα1 | Interleukin-22 receptor subunit alpha-1 | Q8N6P7 |  |
| IL-24 | Interleukin-24 | Q13007 | Y |
| IL-27 | Interleukin 27 | Q8NEV9 | Y |
| Importin β1 | Importin subunit beta-1 | Q14974 |  |
| ING1 | Inhibitor of growth protein 1 | Q9UK53 |  |
| Insulysin | Insulin-degrading enzyme | P14735 | Y |
| Integrin α1β1 | Integrin alpha-1 and Integrin beta-1 | P56199, P05556 | Y |
| IP-10 | Small-inducible cytokine B10 | P02778 | Y |
| IR | Insulin receptor | P06213 | Y |
| I-TAC | Small-inducible cytokine B11 | O14625 | Y |
| ICAM-1, soluble | Intercellular adhesion molecule 1 | P05362 |  |
| ICAM-2, soluble | Intercellular adhesion molecule 2 | P13598 | Y |
| ICAM-3, soluble | Intercellular adhesion molecule 3 | P32942 | Y |
| JAM-B | Junctional adhesion molecule B | P57087 | Y |
| JAM-C | Junctional adhesion molecule C | Q9BX67 | Y |
| Kallikrein 4 | Kallikrein-4 | Q9Y5K2 | Y |
| Kallikrein 5 | Kallikrein-5 | Q9Y337 | Y |
| Kallikrein 6 | Kallikrein-6 | Q92876 | Y |
| Kallikrein 7 | Kallikrein-7 | P49862 | Y |
| Kallikrein 8 | Neuropsin | O60259 | Y |
| Kallikrein 11 | Kallikrein-11 | Q9UBX7 |  |
| Kallikrein 12 | Kallikrein-12 | Q9UKR0 | Y |
| Kallikrein 13 | Kallikrein-13 | Q9UKR3 | Y |
| Kallikrein 14 | Kallikrein-14 | Q9P0G3 |  |
| Kallistatin | Kallistatin | P29622 | Y |
| Karyopherin-α2 | Importin subunit alpha-2 | P52292 | Y |
| Kininogen, HMW, Single Chain | Kininogen-1 (single chain form) | P01042 | Y |
| Kininogen, HMW, Two Chain | Kininogen-1 (two-chain form) | P01042 | Y |
| Kremen2 | Kremen protein 2 | Q8NCW0 | Y |
| Ku70 | ATP-dependent DNA helicase 2 subunit 1 | P12956 | Y |
| Lactoferrin | Lactotransferrin | P02788 | Y |
| LAG-1 | Macrophage inflammatory protein-1b2 | Q8NHW4 | Y |
| Lamin-B1 | Lamin-B1 | P20700 |  |
| Laminin | Laminin subunits alpha-1, beta-1, and gamma-1 | P25391, P07942, P11047 | Y |
| Langerin | C-type lectin domain family 4 member K | Q9UJ71 | Y |
| Layilin | Layilin | Q6UX15 | Y |
| LBP | Lipopolysaccharide-binding protein | P18428 | Y |
| LCK | Proto-oncogene tyrosine-protein kinase LCK | P06239 | Y |
| LD78-β | Small-inducible cytokine A3-like 1 | P16619 | Y |
| LDH-H 1 | L-lactate dehydrogenase B chain | P07195 |  |
| Legumain | Legumain | Q99538 |  |
| Leptin | Leptin | P41159 | Y |
| Lipocalin 2 | Neutrophil gelatinase-associated lipocalin | P80188 | Y |
| LOX-1 | Oxidized low-density lipoprotein receptor 1 | P78380 | Y |
| LRIG3 | Leucine-rich repeats and immunoglobulin-like domains protein 3 | Q6UXM1 | Y |
| LRPAP | Alpha-2-macroglobulin receptor-associated protein | P30533 | Y |
| LSAMP | Limbic system-associated membrane protein | Q13449 | Y |
| LTA-4 hydrolase | Leukotriene A-4 hydrolase | P09960 |  |
| Luteinizing hormone | Glycoprotein hormones alpha chain and Lutropin subunit beta | P01215, P01229 | Y |
| LY9 | T-lymphocyte surface antigen Ly-9 | Q9HBG7 | Y |
| Lymphotactin | Lymphotactin | P47992 | Y |
| Lymphotoxin α1/β2 | Lymphotoxin-alpha (1) and Lymphotoxin-beta (2) | P01374, Q06643 | Y |
| Lymphotoxin α2/β1 | Lymphotoxin-alpha (2) and Lymphotoxin-beta (1) | P01374, Q06643 | Y |
| Lymphotoxin β R | Tumor necrosis factor receptor superfamily member 3 | P36941 | Y |
| LYN A | Tyrosine-protein kinase Lyn | P07948 | Y |
| LYN B | Tyrosine-protein kinase Lyn, isoform B | P07948-2 | Y |
| Lysozyme | Lysozyme C | P61626 | Y |
| LYVE-1 | Lymphatic vessel endothelial hyaluronic acid receptor 1 | Q9Y5Y7 | Y |
| Macrophage mannose receptor | Macrophage mannose receptor 1 | P22897 | Y |
| Macrophage scavenger receptor | Macrophage scavenger receptor types I and II | P21757 |  |
| MAP2K2 | Dual specificity mitogen-activated protein kinase kinase 2 | P36507 |  |
| MAPK1 | Mitogen-activated protein kinase 1 | P28482 | Y |
| MAPK12 | Mitogen-activated protein kinase 12 | P53778 |  |
| MAPK13 | Mitogen-activated protein kinase 13 | O15264 | Y |
| MAPK14 | Mitogen-activated protein kinase 14 | Q16539 | Y |
| MAPK3 | Mitogen-activated protein kinase 3 | P27361 | Y |
| MAPK8 | Mitogen-activated protein kinase 8 | P45983 |  |
| MAPKAPK2 | MAP kinase-activated protein kinase 2 | P49137 |  |
| MAPKAPK3 | MAP kinase-activated protein kinase 3 | Q16644 |  |
| MAPKAPK5 | MAP kinase-activated protein kinase 5 | Q8IW41 |  |
| Marapsin | Serine protease 27 | Q9BQR3 |  |
| MASP3 | Complement-activating component of Ra-reactive factor splice variant MASP3 | Q96RS4 |  |
| MATK | Megakaryocyte-associated tyrosine-protein kinase | P42679 | Y |
| Matrilin-2 | Matrilin-2 | O00339 | Y |
| Matrilin-3 | Matrilin-3 | O15232 | Y |
| MBD4 | Methyl-CpG-binding domain protein 4 | O95243 |  |
| MBL | Mannose-binding protein C | P11226 | Y |
| MCM2 | DNA replication licensing factor MCM2 | P49736 |  |
| MCP-1 | Small-inducible cytokine A2 | P13500 | Y |
| MCP-2 | Small-inducible cytokine A8 | P80075 | Y |
| MCP-3 | Small-inducible cytokine A7 | P80098 | Y |
| MCP-4 | Small-inducible cytokine A13 | Q99616 | Y |
| M-CSF | Macrophage colony-stimulating factor 1 | P09603 |  |
| M-CSF R | Macrophage colony-stimulating factor 1 receptor | P07333 | Y |
| MD-1 | Lymphocyte antigen 86 | O95711 | Y |
| MDC | Small-inducible cytokine A22 | O00626 | Y |
| MDHC | Malate dehydrogenase, cytoplasmic | P40925 |  |
| Mediator complex subunit 1 | Mediator of RNA polymerase II transcription subunit 1 | Q15648 |  |
| MEK1 | Dual specificity mitogen-activated protein kinase kinase 1 | Q02750 | Y |
| MEPE | Matrix extracellular phosphoglycoprotein | Q9NQ76 | Y |
| Mesothelin | Mesothelin | Q13421 |  |
| MetAP 1 | Methionine aminopeptidase 1 | P53582 | Y |
| MetAP2 | Methionine aminopeptidase 2 | P50579 |  |
| MFRP | Membrane frizzled-related protein | Q9BY79 |  |
| MIA | Melanoma-derived growth regulatory protein | Q16674 | Y |
| MICA | MHC class I chain-related protein A | Q29983 | Y |
| Midkine | Midkine | P21741 | Y |
| MIG | Small-inducible cytokine B9 | Q07325 | Y |
| MIP-1α | C-C motif chemokine 3 | P10147 | Y |
| MIP-1β | Small-inducible cytokine A4 | P13236 | Y |
| MIP-3α | Small-inducible cytokine A20 | P78556 | Y |
| MIP-3β | Small-inducible cytokine A19 | Q99731 | Y |
| MIP-4 | C-C motif chemokine 18 | P55774 | Y |
| MIP-5 | C-C motif chemokine 15 | Q16663 | Y |
| MMP-2 | 72 kDa type IV collagenase | P08253 |  |
| MMP-3 | Stromelysin-1 | P08254 |  |
| MMP-7 | Matrilysin | P09237 | Y |
| MMP-8 | Neutrophil collagenase | P22894 | Y |
| MMP-9 | Matrix metalloproteinase-9 | P14780 | Y |
| MMP-10 | Stromelysin-2 | P09238 | Y |
| MMP-17 | Matrix metalloproteinase-17 | Q9ULZ9 |  |
| MOZ | Histone acetyltransferase MYST3 | Q92794 | Y |
| MPIF-1 | C-C motif chemokine 23 | P55773 | Y |
| MRC2 | Macrophage mannose receptor 2 | Q9UBG0 | Y |
| MRCKβ | Serine/threonine-protein kinase MRCK beta | Q9Y5S2 |  |
| MSP R | Macrophage-stimulating protein receptor | Q04912 | Y |
| Myeloperoxidase | Myeloperoxidase | P05164 | Y |
| Myoglobin | Myoglobin | P02144 | Y |
| Myosin regulatory light chain 2 | Myosin regulatory light chain 2, ventricular/cardiac muscle isoform | P10916 | Y |
| NACα | Nascent polypeptide-associated complex subunit alpha | Q13765 |  |
| NADPH-P450 Oxidoreductase | NADPH--cytochrome P450 reductase | P16435 | Y |
| NAGK | N-acetyl-D-glucosamine kinase | Q9UJ70 |  |
| NANOG | Homeobox protein NANOG | Q9H9S0 | Y |
| NAP-2 | Neutrophil-activating peptide 2 | P02775 | Y |
| Nectin-like protein 1 | Cell adhesion molecule 3 | Q8N126 |  |
| Nectin-like protein 2 | Cell adhesion molecule 1 | Q9BY67 | Y |
| Neprilysin-2 | Membrane metallo-endopeptidase-like 1 | Q495T6 |  |
| Netrin-4 | Netrin-4 | Q9HB63 | Y |
| NEUREGULIN-1 | Neuregulin-1 | Q02297 | Y |
| Neurotrophin-3 | Neurotrophin-3 | P20783 | Y |
| Neurotrophin-5 | Neurotrophin-5 | P34130 | Y |
| Nidogen | Nidogen-1 | P14543 | Y |
| Nidogen-2 | Nidogen-2 | Q14112 |  |
| NKG2D | NKG2-D type II integral membrane protein | P26718 | Y |
| NKp30 | Natural cytotoxicity triggering receptor 3 | O14931 | Y |
| NKp44 | Natural cytotoxicity triggering receptor 2 | O95944 | Y |
| Noggin | Noggin | Q13253 | Y |
| Nogo Receptor | Reticulon-4 receptor | Q9BZR6 | Y |
| NovH | Protein NOV homolog | P48745 | Y |
| NRP1 | Neuropilin-1 | O14786 | Y |
| OBCAM | Opioid-binding protein/cell adhesion molecule | Q14982 |  |
| OCIAD1 | OCIA domain-containing protein 1 | Q9NX40 |  |
| Oncostatin M | Oncostatin-M | P13725 | Y |
| RUNX-2 | Runt-related transcription factor 2 | Q13950 | Y |
| Osteonectin | SPARC | P09486 | Y |
| Osteoprotegerin | Tumor necrosis factor receptor superfamily member 11B | O00300 | Y |
| Otubain-1 | Ubiquitin thioesterase OTUB1 | Q96FW1 |  |
| OX40 Ligand | Tumor necrosis factor ligand superfamily member 4 | P23510 | Y |
| p27Kip1 | Cyclin-dependent kinase inhibitor 1B | P46527 |  |
| PAFAH β subunit | Platelet-activating factor acetylhydrolase IB subunit beta | P68402 | Y |
| PAI-1 | Plasminogen activator inhibitor 1 | P05121 | Y |
| PAK3 | Serine/threonine-protein kinase PAK 3 | O75914 | Y |
| PAK6 | Serine/threonine-protein kinase PAK 6 | Q9NQU5 |  |
| PAK7 | Serine/threonine-protein kinase PAK 7 | Q9P286 | Y |
| PAPP-A | Pappalysin-1 | Q13219 | Y |
| P-Cadherin | Cadherin-3 | P22223 | Y |
| PCNA | Proliferating cell nuclear antigen | P12004 | Y |
| PDGF Rβ | Beta-type platelet-derived growth factor receptor | P09619 | Y |
| PDGF-AA | Platelet-derived growth factor A chain | P04085 | Y |
| PDGF-BB | Platelet-derived growth factor B chain | P01127 | Y |
| PDGF-CC | Platelet-derived growth factor C chain | Q9NRA1 |  |
| PD-L2 | Programmed cell death 1 ligand 2 | Q9BQ51 | Y |
| PDPK1 | 3-phosphoinositide-dependent protein kinase 1 | O15530 | Y |
| PECAM-1 | Platelet endothelial cell adhesion molecule | P16284 | Y |
| Peptide YY | Peptide YY | P10082 |  |
| Peroxiredoxin-1 | Peroxiredoxin-1 | Q06830 |  |
| Persephin | Persephin | O60542 | Y |
| PF-4 | Platelet factor 4 | P02776 | Y |
| PGRP-S | Peptidoglycan recognition protein | O75594 | Y |
| Phosphoglycerate mutase 1 | Phosphoglycerate mutase 1 | P18669 |  |
| pIgR | Polymeric immunoglobulin receptor | P01833 | Y |
| PIK3Cα/PIK3R1 | Phosphatidylinositol-4,5-bisphosphate 3-kinase catalytic subunit alpha isoform, Phosphatidylinositol 3-kinase regulatory subunit alpha Complex | P42336, P27986 | Y |
| PK3CG | Phosphatidylinositol-4,5-bisphosphate 3-kinase catalytic subunit gamma isoform | P48736 | Y |
| PKB | RAC-alpha serine/threonine-protein kinase | P31749 | Y |
| PKB γ | RAC-gamma serine/threonine-protein kinase | Q9Y243 | Y |
| PKC-α | Protein kinase C alpha type | P17252 | Y |
| PKC-β-II | Protein kinase C beta type (splice variant Beta-II) | P05771 | Y |
| PKC-γ | Protein kinase C gamma type | P05129 |  |
| PKC-δ | Protein kinase C delta type | Q05655 | Y |
| PKC-ζ | Protein kinase C zeta type | Q05513 | Y |
| Plasmin | Plasmin heavy chain A and light chain B | P00747 | Y |
| Plasminogen | Plasminogen | P00747 | Y |
| Pleiotrophin | Pleiotrophin | P21246 | Y |
| PLGF | Placenta growth factor | P49763 | Y |
| PLK-1 | Serine/threonine-protein kinase PLK1 | P53350 | Y |
| PLPP | Pyridoxal phosphate phosphatase | Q96GD0 |  |
| PPAC | Low molecular weight phosphotyrosine protein phosphatase | P24666 |  |
| Prekallikrein | Plasma kallikrein (precursor) | P03952 | Y |
| PRKA C-α | cAMP-dependent protein kinase catalytic subunit alpha | P17612 | Y |
| PRKCI | Protein kinase C iota type | P41743 | Y |
| PRKCQ | Protein kinase C theta type | Q04759 |  |
| PRL | Prolactin | P01236 | Y |
| Properdin | Properdin | P27918 | Y |
| Protease nexin I | Glia-derived nexin | P07093 | Y |
| Proteasome subunit p40 | 26S proteasome non-ATPase regulatory subunit 7 | P51665 |  |
| Proteasome subunit α1 | Proteasome subunit alpha type-1 | P25786 |  |
| Proteasome subunit α6 | Proteasome subunit alpha type-6 | P60900 |  |
| Protein C | Vitamin K-dependent protein C | P04070 | Y |
| Protein C Inhibitor | Plasma serine protease inhibitor | P05154 | Y |
| Protein S | Vitamin K-dependent protein S | P07225 | Y |
| Proteinase-3 | Myeloblastin | P24158 | Y |
| Prothrombin | Prothrombin | P00734 | Y |
| PSA | Prostate-specific antigen | P07288 | Y |
| PSA-ACT | Prostate-specific antigen and Alpha-1-antichymotrypsin | P07288, P01011 | Y |
| P-Selectin | P-selectin | P16109 | Y |
| PSMA | Glutamate carboxypeptidase 2 | Q04609 |  |
| pTEN | Phosphatidylinositol-3,4,5-trisphosphate 3-phosphatase and dual-specificity protein phosphatase PTEN | P60484 | Y |
| PTH | Parathyroid hormone | P01270 |  |
| PTHrP | Parathyroid hormone-related protein | P12272 | Y |
| PTK6 | Tyrosine-protein kinase 6 | Q13882 |  |
| PTP-1B | Tyrosine-protein phosphatase non-receptor type 1 | P18031 | Y |
| Rab GDP dissociation inhibitor β | Rab GDP dissociation inhibitor beta | P50395 | Y |
| RAC1 | Ras-related C3 botulinum toxin substrate 1 | P63000 | Y |
| RACK1 | Guanine nucleotide-binding protein subunit beta-2-like 1 | P63244 |  |
| RAD51 | DNA repair protein RAD51 homolog 1 | Q06609 | Y |
| RANTES | Small-inducible cytokine A5 | P13501 | Y |
| RBP | Retinol-binding protein 4 | P02753 |  |
| RELT | Tumor necrosis factor receptor superfamily member 19L | Q969Z4 | Y |
| Renin | Renin | P00797 | Y |
| resistin | Resistin | Q9HD89 | Y |
| RET | Proto-oncogene tyrosine-protein kinase receptor ret | P07949 | Y |
| RGM-A | Repulsive guidance molecule A | Q96B86 |  |
| RGM-B | RGM domain family member B | Q6NW40 |  |
| RGM-C | Hemojuvelin | Q6ZVN8 | Y |
| RNAbp 39 | RNA-binding protein 39 | Q14498 |  |
| ROR1 | Tyrosine-protein kinase transmembrane receptor ROR1 | Q01973 | Y |
| RPS6Kα3 | Ribosomal protein S6 kinase alpha-3 | P51812 | Y |
| RS3A | 40S ribosomal protein S3a | P61247 |  |
| RS7 | 40S ribosomal protein S7 | P62081 |  |
| RSK-like protein kinase | Ribosomal protein S6 kinase alpha-5 | O75582 |  |
| S100A12 | Protein S100-A12 | P80511 |  |
| SAA | Serum amyloid A protein | P02735 |  |
| SAP | Serum amyloid P-component | P02743 | Y |
| SBDS | Ribosome maturation protein SBDS | Q9Y3A5 |  |
| SCF sR | Mast/stem cell growth factor receptor | P10721 | Y |
| SCGF-α | C-type lectin domain family 11 member A (alpha form) | Q9Y240 | Y |
| SCGF-β | C-type lectin domain family 11 member A (beta form) | Q9Y240 | Y |
| SDF-1α | SDF-1-alpha | P48061 | Y |
| SDF-1β | SDF-1-beta | P48061 | Y |
| Secretin | Secretin | P09683 |  |
| Semaphorin 3A | Semaphorin-3A | Q14563 |  |
| sE-Selectin | E-selectin | P16581 | Y |
| SET9 | Histone-lysine N-methyltransferase, H3 lysine-4 specific SET7 | Q8WTS6 | Y |
| SEZ6L2 | Seizure 6-like protein 2 | Q6UXD5 |  |
| SGTα | Small glutamine-rich tetratricopeptide repeat-containing protein alpha | O43765 |  |
| SHP-2 | Tyrosine-protein phosphatase non-receptor type 11 | Q06124 | Y |
| Siglec-3 | Myeloid cell surface antigen CD33 | P20138 |  |
| Siglec-6 | Sialic acid-binding Ig-like lectin 6 | O43699 | Y |
| Siglec-7 | Sialic acid-binding Ig-like lectin 7 | Q9Y286 | Y |
| Siglec-9 | Sialic acid-binding Ig-like lectin 9 | Q9Y336 | Y |
| SKP1 | S-phase kinase-associated protein 1 | P63208 |  |
| SLAMF5 | SLAM family member 5 | Q9UIB8 |  |
| SLITRK1 | SLIT and NTRK-like protein 1 | Q96PX8 |  |
| SLPI | Antileukoproteinase | P03973 | Y |
| sL-Selectin | L-selectin | P14151 | Y |
| SMAC/Diablo | Diablo homolog, mitochondrial | Q9NR28 | Y |
| SOD1 | Superoxide dismutase [Cu-Zn] | P00441 | Y |
| Soggy-1 | Dickkopf-like protein 1 | Q9UK85 | Y |
| Somatostatin-28 | Somatostatin-28 | P61278 |  |
| Sonic Hedgehog | Sonic hedgehog protein | Q15465 | Y |
| Sorting nexin 4 | Sorting nexin-4 | O95219 |  |
| sRAGE | Advanced glycosylation end product-specific receptor | Q15109 |  |
| sRANKL | Tumor necrosis factor ligand superfamily member 11 | O14788 | Y |
| SRCN1 | V-src sarcoma (Schmidt-Ruppin A-2) viral oncogene homolog (Avian) | Q76P87 |  |
| Stabilin-2 | Stabilin-2 | Q8WWQ8 | Y |
| sTie-1 | Tyrosine-protein kinase receptor Tie-1 | P35590 | Y |
| sTie-2 | Angiopoietin-1 receptor | Q02763 | Y |
| STK16 | Serine/threonine-protein kinase 16 | O75716 | Y |
| Stress-induced-phosphoprotein 1 | Stress-induced-phosphoprotein 1 | P31948 |  |
| suPAR | Urokinase plasminogen activator surface receptor | Q03405 | Y |
| Survivin | Baculoviral IAP repeat-containing protein 5 | O15392 | Y |
| Syntaxin 1A | Syntaxin-1A | Q16623 | Y |
| TACI | Tumor necrosis factor receptor superfamily member 13B | O14836 | Y |
| TAFI | Carboxypeptidase B2 | Q96IY4 |  |
| TARC | Small-inducible cytokine A17 | Q92583 |  |
| tau | Microtubule-associated protein tau | P10636 | Y |
| TBK1 | Serine/threonine-protein kinase TBK1 | Q9UHD2 | Y |
| TBP | TATA-box-binding protein | P20226 | Y |
| TCPTP | Tyrosine-protein phosphatase non-receptor type 2 | P17706 | Y |
| TEC | Tyrosine-protein kinase Tec | P42680 |  |
| TECK | Small-inducible cytokine A25 | O15444 | Y |
| Tenascin | Tenascin | P24821 | Y |
| Testican-1 | Testican-1 | Q08629 |  |
| Testican-2 | Testican-2 | Q92563 | Y |
| TFPI | Tissue factor pathway inhibitor | P10646 | Y |
| TGF-β1 | Transforming growth factor beta-1 | P01137 | Y |
| TGF-β2 | Transforming growth factor beta-2 | P61812 | Y |
| TGF-β3 | Transforming growth factor beta-3 | P10600 | Y |
| TGF-β R III | TGF-beta receptor type III | Q03167 | Y |
| Thrombin | Thrombin heavy and light chains | P00734 | Y |
| Thrombopoietin | Thrombopoietin | P40225 | Y |
| Thrombopoietin Receptor | Thrombopoietin receptor | P40238 |  |
| Thrombospondin-1 | Thrombospondin-1 | P07996 | Y |
| Thrombospondin-2 | Thrombospondin-2 | P35442 |  |
| Thrombospondin-4 | Thrombospondin-4 | P35443 | Y |
| Thyroglobulin | Thyroglobulin | P01266 |  |
| Thyroid peroxidase | Thyroid peroxidase | P07202 |  |
| Thyroxine-Binding Globulin | Thyroxine-binding globulin | P05543 | Y |
| TIMP-1 | Metalloproteinase inhibitor 1 | P01033 | Y |
| TIMP-2 | Metalloproteinase inhibitor 2 | P16035 | Y |
| TIMP-3 | Metalloproteinase inhibitor 3 | P35625 | Y |
| TLR2 | Toll-like receptor 2 | O60603 |  |
| TLR4 | Toll-like receptor 4 | O00206 |  |
| TNF sR-I | Tumor necrosis factor receptor superfamily member 1A | P19438 | Y |
| TNF sR-II | Tumor necrosis factor receptor superfamily member 1B | P20333 | Y |
| TNFSF15 | Tumor necrosis factor ligand superfamily member 15 | O95150 | Y |
| TNFSF18 | Tumor necrosis factor ligand superfamily member 18 | Q9UNG2 | Y |
| TNR4 | Tumor necrosis factor receptor superfamily member 4 | P43489 |  |
| Topoisomerase I | DNA topoisomerase 1 | P11387 | Y |
| tPA | Tissue-type plasminogen activator | P00750 | Y |
| TRAIL R4 | Tumor necrosis factor receptor superfamily member 10D | Q9UBN6 | Y |
| Transferrin | Serotransferrin | P02787 |  |
| TrATPase | Tartrate-resistant acid phosphatase type 5 | P13686 | Y |
| TrkA | High affinity nerve growth factor receptor | P04629 | Y |
| TrkC | NT-3 growth factor receptor | Q16288 | Y |
| Troponin I | Troponin I, cardiac muscle | P19429 | Y |
| Troponin T | Troponin T, cardiac muscle | P45379 | Y |
| Trypsin | Trypsin-1 | P07477 | Y |
| Trypsin 3 | Trypsin-3 | P35030 | Y |
| Tryptase β-2 | Tryptase beta-2 | P20231 | Y |
| Tryptase γ | Tryptase gamma | Q9NRR2 | Y |
| TSH | Glycoprotein hormones alpha and Thyrotropin subunit beta chains | P01215, P01222 | Y |
| TSLP | Thymic stromal lymphopoietin | Q969D9 | Y |
| TSLP R | Thymic stromal lymphopoietin protein receptor | Q9HC73 | Y |
| TWEAK | Tumor necrosis factor ligand superfamily member 12 | O43508 | Y |
| UB2L3 | Ubiquitin-conjugating enzyme E2 L3 | P68036 |  |
| UBC9 | SUMO-conjugating enzyme UBC9 | P63279 | Y |
| UBE2N | Ubiquitin-conjugating enzyme E2 N | P61088 |  |
| Ubiquitin+1 | Ubiquitin | P62988 | Y |
| UFC1 | Ufm1-conjugating enzyme 1 | Q9Y3C8 | Y |
| UFM1 | Ubiquitin-fold modifier 1 | P61960 |  |
| ULBP-1 | NKG2D ligand 1 | Q9BZM6 | Y |
| ULBP-2 | NKG2D ligand 2 | Q9BZM5 | Y |
| ULBP-3 | NKG2D ligand 3 | Q9BZM4 | Y |
| uPA | Urokinase-type plasminogen activator | P00749 | Y |
| URB | Coiled-coil domain-containing protein 80 | Q76M96 | Y |
| Vasoactive Intestinal Peptide | Vasoactive intestinal peptide | P01282 |  |
| VCAM-1 | Vascular cell adhesion protein 1 | P19320 | Y |
| VEGF | Vascular endothelial growth factor A | P15692 | Y |
| VEGF-C | Vascular endothelial growth factor C | P49767 | Y |
| VEGF-D | Vascular endothelial growth factor D | O43915 | Y |
| VEGF sR2 | Vascular endothelial growth factor receptor 2 | P35968 | Y |
| VEGF sR3 | Vascular endothelial growth factor receptor 3 | P35916 | Y |
| VHR | Dual specificity protein phosphatase 3 | P51452 | Y |
| vWF | von Willebrand factor | P04275 | Y |
| WIF-1 | Wnt inhibitory factor 1 | Q9Y5W5 | Y |
| WISP-1 | WNT1-inducible-signaling pathway protein 1 | O95388 | Y |
| WISP-3 | WNT1-inducible-signaling pathway protein 3 | O95389 | Y |
| WNK3 | Serine/threonine-protein kinase WNK3 | Q9BYP7 | Y |
| XEDAR | Tumor necrosis factor receptor superfamily member 27 | Q9HAV5 | Y |
| X-Pro aminopeptidase 1 | Xaa-Pro aminopeptidase 1 | Q9NQW7 | Y |
| YES | Proto-oncogene tyrosine-protein kinase Yes | P07947 | Y |
| YKL-40 | Chitinase-3-like protein 1 | P36222 |  |
| ZAP70 | Tyrosine-protein kinase ZAP-70 | P43403 | Y |

^§^Indicates proteins measured in the chronic kidney disease study.
