# Supplementary material for: Aptamer-Based Multiplexed Proteomic Technology for Biomarker Discovery
Source: PLoS One. 2010 Dec 7;5(12):e15004. doi: 10.1371/journal.pone.0015004 (PMC3000457; doi:10.1371/journal.pone.0015004)
Supplement: Table S2 — List of limits of quantification for 356 representative proteins measured in buffer. (DOC) [file pone.0015004.s006.doc]

Table S2. Limits of quantification.

| Target protein |  | Concentration (M) (σ_logRFU_ Fit) | | |
| --- | --- | --- | --- | --- |
|  |  | LLOQ | ULOQ | log Range |
| α1-Antichymotrypsin |  | 2.4E-13 | 9.1E-10 | 3.6 |
| α1-Antitrypsin |  | 1.6E-11 | 7.6E-08 | 3.7 |
| α2-Antiplasmin |  | 6.0E-13 | 5.0E-08 | 4.9 |
| α2-HS-Glycoprotein |  | 4.1E-13 | 1.8E-08 | 4.6 |
| α2-Macroglobulin |  | 3.5E-12 | 3.9E-08 | 4.0 |
| Activated Protein C |  | 7.3E-13 | 2.6E-09 | 3.6 |
| Activin A |  | 3.5E-13 | 4.2E-09 | 4.1 |
| ADAMTS-4 |  | 4.8E-13 | 2.7E-10 | 2.8 |
| Aggrecan |  | 1.2E-12 | 2.0E-09 | 3.2 |
| AIF-1 |  | 2.5E-12 | 8.1E-10 | 2.5 |
| Albumin |  | 4.9E-11 | 2.0E-08 | 2.6 |
| Alkaline phosphatase, bone |  | 8.0E-12 | 1.2E-08 | 3.2 |
| ALT |  | 4.3E-12 | 6.9E-09 | 3.2 |
| amyloid precursor protein |  | 2.6E-13 | 7.6E-10 | 3.5 |
| Angiogenin |  | 6.6E-13 | 1.1E-09 | 3.2 |
| Angiopoietin-1 |  | 5.2E-11 | 6.9E-09 | 2.1 |
| Angiopoietin-2 |  | 1.8E-13 | 1.2E-09 | 3.8 |
| Angiopoietin-4 |  | 8.5E-13 | 2.9E-09 | 3.5 |
| Angiostatin |  | 1.5E-13 | 2.4E-09 | 4.2 |
| Angiotensinogen |  | 4.7E-12 | 5.5E-08 | 4.1 |
| Apo A-I |  | 2.0E-11 | 2.5E-08 | 3.1 |
| Apo B |  | 1.1E-12 | 4.5E-09 | 3.6 |
| Apo E |  | 3.5E-11 | 7.8E-09 | 2.4 |
| Apo E2 |  | 1.8E-10 | 6.8E-09 | 1.6 |
| Apo E3 |  | 1.9E-12 | 6.5E-10 | 2.5 |
| Apo E4 |  | 9.1E-13 | 1.3E-09 | 3.2 |
| APRIL |  | 1.3E-10 | 5.1E-09 | 1.6 |
| β2-Microglobulin |  | 5.9E-13 | 1.8E-09 | 3.5 |
| B7 |  | 1.9E-11 | 3.4E-08 | 3.3 |
| BARK1 |  | 1.3E-13 | 5.5E-10 | 3.6 |
| BCA-1 |  | 2.0E-13 | 2.7E-10 | 3.1 |
| Bcl-2 |  | 1.0E-12 | 2.4E-09 | 3.4 |
| BCL2A1 |  | 2.4E-13 | 7.8E-10 | 3.5 |
| BDNF |  | 1.4E-11 | 1.1E-08 | 2.9 |
| βIGH3 |  | 1.5E-12 | 1.3E-09 | 2.9 |
| BMP-1 |  | 9.1E-13 | 1.1E-09 | 3.1 |
| BMP10 |  | 1.3E-10 | 3.4E-08 | 2.4 |
| BMP-14 |  | 1.3E-12 | 2.4E-09 | 3.3 |
| BMP-6 |  | 2.2E-12 | 7.5E-10 | 2.5 |
| BMP-7 |  | 1.5E-12 | 1.3E-09 | 3.0 |
| BMPR1A |  | 1.6E-11 | 2.4E-08 | 3.2 |
| Bone proteoglycan II |  | 7.3E-12 | 1.6E-10 | 1.3 |
| BPI |  | 2.6E-13 | 4.6E-10 | 3.3 |
| BTK |  | 1.6E-13 | 2.0E-09 | 4.1 |
| C1q |  | 8.2E-13 | 2.9E-10 | 2.6 |
| C1r |  | 1.3E-13 | 1.6E-09 | 4.1 |
| C1s |  | 6.7E-12 | 1.6E-08 | 3.4 |
| C2 |  | 5.4E-14 | 6.0E-10 | 4.0 |
| C3 |  | 6.5E-13 | 8.4E-10 | 3.1 |
| C3a |  | 7.0E-11 | 2.4E-08 | 2.5 |
| C3adesArg |  | 4.2E-14 | 4.4E-10 | 4.0 |
| C3b |  | 6.5E-11 | 4.9E-09 | 1.9 |
| C4 |  | 6.0E-14 | 4.6E-09 | 4.9 |
| C4b |  | 7.6E-12 | 7.8E-09 | 3.0 |
| C5 |  | 4.3E-12 | 2.7E-09 | 2.8 |
| C5a |  | 3.4E-13 | 1.3E-09 | 3.6 |
| C5b,6 Complex |  | 2.4E-12 | 9.0E-10 | 2.6 |
| C6 |  | 1.4E-12 | 4.6E-10 | 2.5 |
| C7 |  | 5.6E-12 | 7.9E-09 | 3.2 |
| C8 |  | 2.0E-11 | 4.2E-09 | 2.3 |
| C9 |  | 2.6E-14 | 3.6E-10 | 4.1 |
| Cadherin-1 |  | 1.8E-12 | 7.2E-09 | 3.6 |
| Cadherin-12 |  | 4.3E-10 | 1.0E-06 | 3.4 |
| Cadherin-2 |  | 4.0E-11 | 3.4E-08 | 2.9 |
| Cadherin-5 |  | 3.9E-12 | 7.0E-09 | 3.3 |
| Cadherin-6 |  | 2.3E-12 | 6.8E-09 | 3.5 |
| Calpain I |  | 7.0E-14 | 3.3E-10 | 3.7 |
| CAMK1D |  | 7.9E-11 | 6.1E-10 | 0.9 |
| Carbonic anhydrase VI |  | 5.6E-11 | 1.4E-08 | 2.4 |
| Carbonic anhydrase IX |  | 1.0E-06 | 1.0E-06 | 0.0 |
| Cardiotrophin-1 |  | 1.4E-12 | 1.1E-09 | 2.9 |
| Caspase-3 |  | 4.2E-13 | 3.2E-09 | 3.9 |
| Catalase |  | 7.8E-14 | 8.2E-10 | 4.0 |
| Cathepsin A |  | 5.4E-14 | 1.2E-09 | 4.3 |
| Cathepsin B |  | 5.8E-13 | 5.4E-10 | 3.0 |
| Cathepsin G |  | 5.9E-12 | 1.8E-09 | 2.5 |
| Cathepsin H |  | 8.0E-13 | 2.1E-09 | 3.4 |
| Cathepsin S |  | 2.5E-12 | 1.5E-09 | 2.8 |
| Cathepsin V |  | 8.7E-13 | 6.5E-10 | 2.9 |
| CCL28 |  | 8.8E-13 | 3.6E-10 | 2.6 |
| CD109 |  | 2.1E-14 | 8.2E-10 | 4.6 |
| CD23 |  | 2.0E-13 | 2.4E-10 | 3.1 |
| CD30 |  | 6.9E-13 | 1.7E-09 | 3.4 |
| CD30 Ligand |  | 6.7E-13 | 1.2E-09 | 3.2 |
| CD48 |  | 3.4E-13 | 8.0E-10 | 3.4 |
| CD5L |  | 2.1E-14 | 2.1E-10 | 4.0 |
| CD70 |  | 1.1E-10 | 1.3E-08 | 2.1 |
| CDK1/cyclin B |  | 1.7E-12 | 3.0E-09 | 3.3 |
| Chemerin |  | 2.2E-13 | 4.4E-10 | 3.3 |
| CHL1 |  | 1.9E-13 | 3.4E-08 | 5.3 |
| Chordin-Like 1 |  | 2.3E-12 | 6.5E-10 | 2.5 |
| CNTF |  | 1.1E-13 | 5.7E-10 | 3.7 |
| Coagulation Factor IX |  | 2.4E-13 | 2.8E-09 | 4.1 |
| Coagulation Factor IXab |  | 1.4E-13 | 1.0E-09 | 3.9 |
| Coagulation Factor V |  | 9.0E-13 | 4.8E-10 | 2.7 |
| Coagulation Factor VII |  | 2.1E-12 | 2.1E-09 | 3.0 |
| Coagulation Factor X |  | 2.4E-11 | 1.4E-09 | 1.8 |
| Coagulation Factor Xa |  | 4.3E-13 | 2.0E-10 | 2.7 |
| Coagulation Factor XI |  | 3.8E-13 | 1.9E-09 | 3.7 |
| complement factor H-related 5 |  | 5.2E-13 | 2.4E-10 | 2.7 |
| contactin-1 |  | 7.5E-14 | 5.3E-10 | 3.9 |
| Contactin-4 |  | 8.3E-13 | 1.2E-09 | 3.2 |
| Cryptic |  | 8.8E-13 | 1.4E-09 | 3.2 |
| CSK |  | 5.3E-13 | 7.7E-10 | 3.2 |
| CTACK |  | 2.4E-12 | 1.2E-08 | 3.7 |
| CTLA-4 |  | 6.9E-13 | 1.1E-09 | 3.2 |
| CXCL16, soluble |  | 1.1E-13 | 4.5E-10 | 3.6 |
| Cyclophilin A |  | 1.8E-13 | 9.7E-10 | 3.7 |
| Cystatin C |  | 5.4E-13 | 1.5E-08 | 4.4 |
| Cystatin M |  | 4.8E-13 | 7.7E-10 | 3.2 |
| Cystatin SN |  | 1.1E-12 | 6.4E-09 | 3.8 |
| Cytochrome c |  | 2.2E-12 | 3.1E-09 | 3.2 |
| DAN |  | 2.1E-12 | 4.8E-10 | 2.4 |
| DPP2 |  | 3.6E-12 | 3.1E-09 | 2.9 |
| ECM1 |  | 1.6E-13 | 2.0E-10 | 3.1 |
| EG-VEGF |  | 1.5E-12 | 8.8E-09 | 3.8 |
| eIF-4H |  | 8.6E-11 | 1.5E-08 | 2.2 |
| Endostatin |  | 1.3E-13 | 9.9E-10 | 3.9 |
| Eotaxin |  | 6.2E-11 | 2.5E-09 | 1.6 |
| Eotaxin-2 |  | 1.0E-12 | 1.0E-09 | 3.0 |
| EphA3 |  | 1.5E-12 | 1.1E-09 | 2.9 |
| Ephrin-A5 |  | 4.6E-13 | 2.0E-09 | 3.6 |
| Epo-R |  | 8.2E-11 | 5.5E-09 | 1.8 |
| ERBB1 |  | 3.8E-14 | 3.3E-10 | 3.9 |
| ERBB2 |  | 1.7E-11 | 1.6E-08 | 3.0 |
| ERBB3 |  | 2.9E-13 | 5.2E-10 | 3.2 |
| ERBB4 |  | 1.0E-11 | 2.7E-09 | 2.4 |
| ESAM |  | 2.9E-14 | 3.4E-10 | 4.1 |
| ETHE1 |  | 7.5E-13 | 1.8E-09 | 3.4 |
| Factor B |  | 9.4E-13 | 2.7E-08 | 4.5 |
| Factor D |  | 1.6E-12 | 9.5E-10 | 2.8 |
| Factor H |  | 6.8E-13 | 8.3E-09 | 4.1 |
| Factor I |  | 2.3E-14 | 4.5E-10 | 4.3 |
| Fas ligand, soluble |  | 6.8E-12 | 7.1E-09 | 3.0 |
| FCγ2A |  | 4.9E-13 | 1.1E-09 | 3.4 |
| Ferritin |  | 3.5E-11 | 1.0E-08 | 2.5 |
| Fetuin B |  | 6.9E-13 | 7.1E-11 | 2.0 |
| FGF-16 |  | 1.6E-10 | 4.6E-08 | 2.5 |
| FGF-18 |  | 1.0E-12 | 7.2E-10 | 2.8 |
| FGF-19 |  | 5.7E-13 | 7.9E-10 | 3.1 |
| FGF-20 |  | 7.9E-13 | 4.6E-10 | 2.8 |
| FGF-4 |  | 4.4E-13 | 1.5E-09 | 3.5 |
| FGF-6 |  | 6.1E-13 | 1.8E-09 | 3.5 |
| FGF-7 |  | 2.6E-13 | 9.8E-10 | 3.6 |
| FGF-9 |  | 2.0E-12 | 3.7E-09 | 3.3 |
| Fibrinogen |  | 4.2E-14 | 1.0E-09 | 4.4 |
| Fibronectin |  | 1.7E-12 | 2.4E-09 | 3.2 |
| Ficolin-2 |  | 9.6E-12 | 1.6E-08 | 3.2 |
| Fibronectin FN1.3 |  | 3.6E-14 | 1.3E-09 | 4.6 |
| Fibronectin FN1.4 |  | 1.0E-12 | 4.2E-09 | 3.6 |
| Fractalkine/CX3CL-1 |  | 2.9E-12 | 5.6E-10 | 2.3 |
| FSH |  | 3.6E-12 | 2.6E-09 | 2.9 |
| FSTL3 |  | 7.3E-14 | 3.5E-10 | 3.7 |
| GAPDH |  | 3.7E-13 | 1.1E-09 | 3.5 |
| GCP-2 |  | 8.7E-13 | 7.5E-10 | 2.9 |
| GDF-11 |  | 1.2E-12 | 2.1E-10 | 2.2 |
| gp130, soluble |  | 9.4E-13 | 1.4E-09 | 3.2 |
| Granulysin |  | 2.1E-13 | 7.0E-10 | 3.5 |
| Granzyme A |  | 4.3E-14 | 3.9E-10 | 4.0 |
| Group IIA phospholipase A2 |  | 6.5E-13 | 4.6E-10 | 2.9 |
| Growth hormone receptor |  | 4.8E-12 | 7.4E-09 | 3.2 |
| Gro-α |  | 6.5E-13 | 1.4E-09 | 3.3 |
| Gro-γ |  | 9.5E-13 | 1.1E-09 | 3.1 |
| GSK-3 α |  | 1.0E-06 | 1.0E-06 | 0.0 |
| GSK-3 β |  | 2.2E-12 | 1.3E-08 | 3.8 |
| HAI-2 |  | 4.0E-13 | 6.3E-10 | 3.2 |
| Haptoglobin, Mixed Type |  | 1.9E-11 | 8.7E-08 | 3.7 |
| HCC-1 |  | 9.9E-13 | 4.3E-09 | 3.6 |
| HCC-4 |  | 1.1E-12 | 1.2E-09 | 3.0 |
| Hemopexin |  | 1.5E-12 | 1.0E-06 | 5.8 |
| Heparin cofactor II |  | 4.6E-13 | 3.0E-08 | 4.8 |
| Hepcidin-25 |  | 1.3E-12 | 1.5E-09 | 3.1 |
| HGF activator |  | 5.9E-14 | 7.5E-11 | 3.1 |
| HMG-1 |  | 1.4E-12 | 2.3E-09 | 3.2 |
| HSP 60 |  | 4.8E-13 | 5.9E-10 | 3.1 |
| HSP 70 |  | 9.6E-12 | 1.8E-10 | 1.3 |
| HSP 90α |  | 1.4E-12 | 1.2E-09 | 2.9 |
| HSP 90β |  | 3.5E-12 | 2.1E-09 | 2.8 |
| iC3b |  | 1.9E-13 | 9.0E-10 | 3.7 |
| ICOS |  | 1.1E-11 | 8.0E-09 | 2.9 |
| IFN-γ |  | 6.6E-13 | 4.2E-10 | 2.8 |
| IgE |  | 3.2E-13 | 2.3E-09 | 3.9 |
| IGFBP-1 |  | 2.1E-14 | 7.2E-10 | 4.5 |
| IGFBP-2 |  | 9.4E-13 | 2.4E-09 | 3.4 |
| IGFBP-3 |  | 6.6E-12 | 1.9E-09 | 2.5 |
| IGFBP-4 |  | 2.5E-13 | 2.0E-10 | 2.9 |
| IGFBP-5 |  | 3.1E-13 | 3.6E-10 | 3.1 |
| IGFBP-6 |  | 1.5E-13 | 1.1E-09 | 3.8 |
| IGFBP-7 |  | 7.4E-14 | 3.1E-10 | 3.6 |
| IGF-I |  | 3.8E-13 | 7.4E-10 | 3.3 |
| IGF-II receptor |  | 4.6E-12 | 2.0E-09 | 2.6 |
| IgM |  | 8.2E-12 | 4.0E-09 | 2.7 |
| IL-1 R4 |  | 4.1E-12 | 5.6E-10 | 2.1 |
| IL-10 |  | 2.5E-12 | 1.5E-09 | 2.8 |
| IL-11 |  | 6.3E-12 | 2.3E-08 | 3.6 |
| IL-12 |  | 1.4E-10 | 6.7E-09 | 1.7 |
| IL-13 |  | 9.9E-13 | 7.9E-10 | 2.9 |
| IL-15 Rα |  | 1.9E-13 | 2.4E-10 | 3.1 |
| IL-16 |  | 9.4E-14 | 7.7E-11 | 2.9 |
| IL-17 |  | 8.5E-13 | 3.3E-09 | 3.6 |
| IL-17B |  | 4.0E-13 | 3.3E-10 | 2.9 |
| IL-18 BPa |  | 1.4E-11 | 1.5E-09 | 2.0 |
| IL-2 |  | 1.7E-13 | 1.3E-09 | 3.9 |
| IL-2 sRγ |  | 5.1E-12 | 5.4E-09 | 3.0 |
| IL-4 |  | 2.7E-13 | 8.4E-10 | 3.5 |
| IL-4 sR |  | 2.4E-12 | 4.7E-09 | 3.3 |
| IL-6 |  | 1.3E-12 | 1.0E-09 | 2.9 |
| IL-6 sRα |  | 3.0E-13 | 8.0E-10 | 3.4 |
| IL-8 |  | 7.3E-14 | 4.0E-10 | 3.7 |
| ING1 |  | 1.8E-12 | 3.9E-09 | 3.3 |
| Integrin α1β1 |  | 1.3E-11 | 7.9E-10 | 1.8 |
| I-TAC |  | 7.6E-13 | 5.8E-10 | 2.9 |
| Kallikrein 4 |  | 1.6E-11 | 8.3E-09 | 2.7 |
| Kallikrein 7 |  | 1.7E-12 | 5.6E-10 | 2.5 |
| Kallikrein 8 |  | 8.2E-12 | 6.7E-09 | 2.9 |
| Kallikrein 12 |  | 1.3E-12 | 6.1E-10 | 2.7 |
| Kallistatin |  | 7.4E-14 | 9.7E-10 | 4.1 |
| Kininogen, HMW, Single Chain |  | 8.4E-13 | 2.3E-10 | 2.4 |
| Lactoferrin |  | 1.4E-13 | 1.4E-09 | 4.0 |
| LAG-1 |  | 1.6E-13 | 5.5E-10 | 3.6 |
| Lamin-B1 |  | 7.8E-14 | 8.5E-10 | 4.0 |
| LBP |  | 2.6E-13 | 2.3E-10 | 2.9 |
| LD78-β |  | 7.2E-13 | 2.5E-09 | 3.5 |
| LDH-H 1 |  | 8.4E-13 | 3.9E-09 | 3.7 |
| Leptin |  | 2.4E-12 | 8.6E-09 | 3.6 |
| Leptin |  | 8.3E-14 | 1.1E-09 | 4.1 |
| Lipocalin 2 |  | 2.9E-13 | 1.6E-09 | 3.7 |
| LRIG3 |  | 1.2E-13 | 5.2E-10 | 3.6 |
| Luteinizing hormone |  | 3.7E-13 | 4.5E-09 | 4.1 |
| Lymphotactin |  | 2.7E-13 | 2.7E-10 | 3.0 |
| Lymphotoxin α1/β2 |  | 4.8E-13 | 1.7E-09 | 3.6 |
| Lymphotoxin α2/β1 |  | 4.3E-12 | 1.1E-08 | 3.4 |
| LYN A |  | 6.7E-13 | 3.2E-09 | 3.7 |
| LYVE-1 |  | 8.8E-13 | 7.0E-10 | 2.9 |
| Macrophage mannose receptor |  | 1.0E-11 | 6.6E-10 | 1.8 |
| MAPK14 |  | 2.6E-12 | 4.4E-09 | 3.2 |
| MAPK3 |  | 8.5E-13 | 3.4E-09 | 3.6 |
| MAPKAPK2 |  | 1.9E-11 | 8.4E-09 | 2.6 |
| MAPKAPK5 |  | 8.5E-11 | 8.4E-10 | 1.0 |
| MCP-1 |  | 5.4E-13 | 5.2E-10 | 3.0 |
| MCP-2 |  | 1.5E-12 | 9.8E-09 | 3.8 |
| MCP-3 |  | 2.1E-12 | 1.0E-08 | 3.7 |
| MCP-4 |  | 5.1E-12 | 6.1E-10 | 2.1 |
| M-CSF R |  | 5.1E-13 | 9.0E-10 | 3.2 |
| MD-1 |  | 1.6E-12 | 3.6E-08 | 4.3 |
| MDHC |  | 2.9E-13 | 2.0E-09 | 3.8 |
| Mesothelin |  | 3.0E-12 | 1.3E-09 | 2.6 |
| Mesothelin |  | 9.8E-12 | 9.8E-09 | 3.0 |
| MetAP 1 |  | 9.3E-13 | 2.1E-09 | 3.4 |
| MIA |  | 2.2E-13 | 1.7E-09 | 3.9 |
| Midkine |  | 4.0E-11 | 7.2E-09 | 2.3 |
| MIP-1α |  | 3.4E-12 | 5.8E-10 | 2.2 |
| MIP-1β |  | 6.8E-10 | 1.0E-06 | 3.2 |
| MIP-4 |  | 1.3E-13 | 3.6E-10 | 3.4 |
| MIP-5 |  | 2.4E-13 | 9.3E-10 | 3.6 |
| MMP-2 |  | 4.6E-12 | 1.2E-09 | 2.4 |
| MMP-3 |  | 3.1E-10 | 1.9E-08 | 1.8 |
| MMP-7 |  | 2.5E-13 | 1.0E-09 | 3.6 |
| MMP-8 |  | 1.7E-12 | 2.8E-09 | 3.2 |
| MMP-9 |  | 5.8E-13 | 3.8E-09 | 3.8 |
| MMP-10 |  | 1.6E-12 | 1.8E-09 | 3.1 |
| MPIF-1 |  | 1.2E-13 | 5.2E-10 | 3.7 |
| MRC2 |  | 1.3E-13 | 3.3E-10 | 3.4 |
| Myeloperoxidase |  | 2.0E-13 | 1.3E-09 | 3.8 |
| Myoglobin |  | 2.4E-14 | 6.9E-10 | 4.5 |
| Myosin regulatory light chain 2 |  | 1.2E-11 | 2.9E-09 | 2.4 |
| NADPH-P450 Oxidoreductase |  | 8.0E-13 | 9.7E-10 | 3.1 |
| NAP-2 |  | 2.5E-14 | 2.3E-10 | 4.0 |
| Netrin-4 |  | 3.1E-13 | 5.6E-10 | 3.3 |
| Neurotrophin-3 |  | 1.9E-13 | 7.5E-10 | 3.6 |
| Neurotrophin-5 |  | 6.7E-13 | 6.9E-10 | 3.0 |
| Nidogen-2 |  | 1.4E-12 | 1.6E-08 | 4.1 |
| Noggin |  | 1.2E-11 | 4.4E-09 | 2.6 |
| NRP1 |  | 1.0E-14 | 2.2E-10 | 4.3 |
| OBCAM |  | 4.8E-11 | 1.0E-06 | 4.3 |
| RUNX-2 |  | 2.4E-13 | 7.2E-09 | 4.5 |
| Osteonectin |  | 1.0E-06 | 1.0E-06 | 0.0 |
| Otubain-1 |  | 5.4E-12 | 7.1E-08 | 4.1 |
| OX40 Ligand |  | 4.0E-12 | 7.6E-10 | 2.3 |
| PAI-1 |  | 2.8E-12 | 1.0E-06 | 5.6 |
| PAPP-A |  | 4.5E-13 | 6.1E-10 | 3.1 |
| P-Cadherin |  | 4.5E-12 | 2.6E-09 | 2.8 |
| Protein C Inhibitor |  | 1.1E-12 | 1.8E-09 | 3.2 |
| PCNA |  | 2.1E-12 | 1.8E-09 | 2.9 |
| PDGF-BB |  | 2.2E-13 | 6.5E-10 | 3.5 |
| PF-4 |  | 6.2E-14 | 8.8E-10 | 4.2 |
| PGRP-S |  | 4.4E-14 | 8.9E-10 | 4.3 |
| PKC-ζ |  | 2.7E-11 | 2.8E-08 | 3.0 |
| Plasminogen |  | 1.3E-12 | 6.3E-09 | 3.7 |
| PLPP |  | 3.0E-12 | 5.8E-09 | 3.3 |
| Prekallikrein |  | 3.5E-13 | 1.2E-09 | 3.5 |
| Properdin |  | 1.5E-12 | 2.2E-08 | 4.2 |
| Protease nexin I |  | 1.1E-12 | 3.7E-09 | 3.5 |
| Proteasome subunit p40 |  | 5.3E-12 | 1.3E-08 | 3.4 |
| Protein C |  | 8.2E-13 | 2.0E-08 | 4.4 |
| Protein S |  | 1.4E-12 | 5.9E-10 | 2.6 |
| Proteinase-3 |  | 3.6E-12 | 1.3E-09 | 2.6 |
| Prothrombin |  | 1.4E-12 | 1.0E-08 | 3.9 |
| PSA |  | 1.3E-12 | 1.6E-09 | 3.1 |
| PSA-ACT |  | 6.6E-14 | 5.4E-10 | 3.9 |
| P-Selectin |  | 1.0E-14 | 4.0E-10 | 4.6 |
| pTEN |  | 1.7E-12 | 1.6E-09 | 3.0 |
| RANTES |  | 7.0E-12 | 3.1E-09 | 2.7 |
| RBP |  | 9.1E-11 | 7.7E-08 | 2.9 |
| Renin |  | 2.6E-13 | 4.8E-10 | 3.3 |
| resistin |  | 1.4E-12 | 7.6E-10 | 2.7 |
| RGM-B |  | 1.9E-13 | 3.5E-10 | 3.3 |
| ROR1 |  | 3.7E-13 | 2.4E-09 | 3.8 |
| RPS6Kα3 |  | 4.1E-13 | 5.0E-10 | 3.1 |
| S100A12 |  | 1.0E-06 | 1.0E-06 | 0.0 |
| SAP |  | 3.2E-13 | 1.8E-09 | 3.8 |
| SCF sR |  | 3.1E-12 | 5.1E-10 | 2.2 |
| sE-Selectin |  | 1.1E-13 | 1.4E-10 | 3.1 |
| ICAM-2, soluble |  | 8.9E-11 | 8.9E-09 | 2.0 |
| ICAM-3, soluble |  | 2.9E-12 | 9.5E-09 | 3.5 |
| Siglec-9 |  | 6.0E-14 | 1.8E-10 | 3.5 |
| SLPI |  | 9.3E-13 | 4.8E-09 | 3.7 |
| sL-Selectin |  | 2.0E-13 | 1.4E-09 | 3.8 |
| Sonic Hedgehog |  | 1.1E-13 | 2.3E-09 | 4.3 |
| Sorting nexin 4 |  | 7.4E-13 | 2.5E-09 | 3.5 |
| sTie-1 |  | 1.4E-12 | 4.2E-10 | 2.5 |
| STK16 |  | 3.4E-12 | 7.5E-10 | 2.3 |
| tau |  | 1.2E-10 | 3.2E-08 | 2.4 |
| Tenascin |  | 6.7E-12 | 1.2E-09 | 2.3 |
| TFPI |  | 4.2E-14 | 6.4E-10 | 4.2 |
| TGF-β1 |  | 2.4E-12 | 5.7E-09 | 3.4 |
| TGF-β2 |  | 1.0E-12 | 3.4E-09 | 3.5 |
| TGF-β3 |  | 9.6E-12 | 6.9E-09 | 2.9 |
| Thrombin |  | 6.5E-13 | 1.1E-09 | 3.2 |
| Thrombospondin-4 |  | 1.6E-13 | 1.9E-09 | 4.1 |
| Thyroid peroxidase |  | 1.8E-13 | 1.2E-09 | 3.8 |
| Thyroxine-Binding Globulin |  | 1.4E-13 | 4.6E-10 | 3.5 |
| TIMP-1 |  | 1.6E-13 | 2.0E-09 | 4.1 |
| TIMP-2 |  | 6.4E-11 | 6.8E-08 | 3.0 |
| TIMP-3 |  | 2.3E-13 | 1.6E-09 | 3.8 |
| TNF sR-I |  | 5.6E-13 | 1.2E-09 | 3.3 |
| TNF sR-II |  | 2.8E-12 | 2.1E-09 | 2.9 |
| TNFSF18 |  | 8.7E-13 | 8.0E-10 | 3.0 |
| tPA |  | 7.4E-12 | 7.0E-10 | 2.0 |
| TRAIL R4 |  | 4.7E-12 | 3.3E-10 | 1.8 |
| Transferrin |  | 1.9E-12 | 4.7E-09 | 3.4 |
| TrATPase |  | 1.4E-13 | 1.3E-09 | 4.0 |
| Troponin I |  | 1.7E-11 | 4.2E-09 | 2.4 |
| Troponin T |  | 5.0E-11 | 3.4E-09 | 1.8 |
| TSLP |  | 1.0E-12 | 1.4E-09 | 3.1 |
| UBC9 |  | 2.6E-14 | 3.8E-10 | 4.2 |
| Ubiquitin+1 |  | 1.2E-12 | 1.7E-09 | 3.2 |
| URB |  | 3.8E-13 | 3.7E-10 | 3.0 |
| VCAM-1 |  | 4.9E-12 | 1.1E-08 | 3.4 |
| VEGF |  | 9.4E-14 | 3.9E-09 | 4.6 |
| VEGF sR2 |  | 3.8E-13 | 1.1E-09 | 3.5 |
| VEGF sR3 |  | 8.3E-13 | 2.1E-09 | 3.4 |
| vWF |  | 1.1E-11 | 8.0E-10 | 1.9 |
| WIF-1 |  | 1.2E-12 | 1.4E-09 | 3.1 |
| WISP-1 |  | 2.9E-12 | 5.7E-10 | 2.3 |
| X-Pro aminopeptidase 1 |  | 2.4E-12 | 3.7E-09 | 3.2 |
