# Supplementary material for: Aptamer-Based Multiplexed Proteomic Technology for Biomarker Discovery
Source: PLoS One. 2010 Dec 7;5(12):e15004. doi: 10.1371/journal.pone.0015004 (PMC3000457; doi:10.1371/journal.pone.0015004)
Supplement: Table S3 — List of measured limits of quantification for proteins spiked into buffer and plasma. (DOC) [file pone.0015004.s007.doc]

Table S3. Limits of quantification for buffer and plasma spiked proteins.

|  |  | Concentration (M) (σ_logRFU_ Fit) | | | | | | |
| --- | --- | --- | --- | --- | --- | --- | --- | --- |
|  |  | Plasma | | |  | Buffer | | |
| Target |  | LLOQ | ULOQ | log Range |  | LLOQ | ULOQ | log Range |
| Activin A |  | 3.6E-12 | 1.1E-09 | 2.5 |  | 6.5E-13 | 7.5E-10 | 3.1 |
| ADAMTS-4 |  | 1.0E-12 | 5.1E-10 | 2.7 |  | 1.1E-13 | 2.8E-10 | 3.4 |
| CNTF |  | 3.6E-13 | 7.1E-10 | 3.3 |  | 9.4E-14 | 2.2E-10 | 3.4 |
| CTLA-4 |  | 3.1E-12 | 6.4E-09 | 3.3 |  | 1.9E-12 | 3.8E-09 | 3.3 |
| EG-VEGF |  | 1.8E-12 | 2.8E-09 | 3.2 |  | 2.4E-12 | 3.0E-09 | 3.1 |
| Ephrin-A5 |  | 3.0E-12 | 1.6E-09 | 2.7 |  | 1.0E-12 | 5.2E-10 | 2.7 |
| FGF-4 |  | 1.5E-11 | 1.7E-08 | 3.1 |  | 7.7E-12 | 1.1E-08 | 3.1 |
| FGF-6 |  | 3.8E-12 | 2.9E-09 | 2.9 |  | 4.8E-12 | 9.9E-10 | 2.3 |
| FGF-9 |  | 1.1E-10 | 9.0E-09 | 1.9 |  | 3.7E-11 | 6.3E-09 | 2.2 |
| FGF-20 |  | 1.7E-12 | 6.5E-10 | 2.6 |  | 1.1E-12 | 2.7E-10 | 2.4 |
| Granzyme A |  | 5.2E-13 | 6.5E-10 | 3.1 |  | 1.6E-13 | 4.3E-10 | 3.4 |
| HAI-2 |  | 5.5E-13 | 8.7E-10 | 3.2 |  | 3.3E-13 | 2.7E-10 | 2.9 |
| IL-2 |  | 9.2E-13 | 1.4E-09 | 3.2 |  | 3.8E-13 | 6.2E-10 | 3.2 |
| IL-4 |  | 6.3E-13 | 8.4E-10 | 3.1 |  | 1.1E-12 | 2.0E-10 | 2.2 |
| IL-11 |  | 5.1E-12 | 1.0E-06 | 5.3 |  | 7.6E-12 | 5.1E-08 | 3.8 |
| IL-17B |  | 1.3E-12 | 1.8E-09 | 3.2 |  | 7.0E-12 | 5.5E-10 | 1.9 |
| IL-4 sR |  | 1.6E-11 | 5.9E-09 | 2.6 |  | 3.4E-12 | 6.5E-09 | 3.3 |
| I-TAC |  | 4.1E-12 | 1.4E-09 | 2.5 |  | 2.2E-12 | 8.8E-10 | 2.6 |
| Lymphotoxin α1/β2 |  | 6.4E-12 | 2.8E-08 | 3.6 |  | 1.7E-12 | 7.5E-09 | 3.6 |
| MCP-2 |  | 9.3E-12 | 5.5E-09 | 2.8 |  | 3.0E-12 | 2.2E-09 | 2.9 |
| MCP-3 |  | 1.7E-11 | 7.7E-09 | 2.7 |  | 1.5E-11 | 6.6E-09 | 2.7 |
| Neurotrophin-3 |  | 6.5E-13 | 8.4E-10 | 3.1 |  | 1.4E-12 | 4.9E-10 | 2.5 |
| PSA |  | 3.3E-11 | 1.0E-08 | 2.5 |  | 2.4E-12 | 1.7E-09 | 2.8 |
| Sonic Hedgehog |  | 1.5E-12 | 1.3E-09 | 2.9 |  | 1.4E-12 | 5.4E-10 | 2.6 |
| TGF-β2 |  | 1.6E-12 | 1.0E-09 | 2.8 |  | 1.8E-12 | 7.4E-10 | 2.6 |
| TNF sR-I |  | 5.2E-12 | 1.0E-09 | 2.3 |  | 1.6E-12 | 7.3E-10 | 2.7 |
| TNF sR-II |  | 2.9E-12 | 6.5E-09 | 3.4 |  | 3.5E-12 | 1.3E-08 | 3.6 |
| TNFSF18 |  | 2.3E-12 | 8.3E-10 | 2.6 |  | 9.1E-13 | 4.3E-10 | 2.7 |
