# Supplementary material for: Aptamer-Based Multiplexed Proteomic Technology for Biomarker Discovery
Source: PLoS One. 2010 Dec 7;5(12):e15004. doi: 10.1371/journal.pone.0015004 (PMC3000457; doi:10.1371/journal.pone.0015004)
Supplement: Table S4 — List of 60 proteins identified that varied between early and late stage CKD with a q‐value of 4.2×10^−4^. (DOC) [file pone.0015004.s008.doc]

**Table S4. Potential CKD Biomarkers**

| Target | p-value | q-value | Mol. Mass (kDa) |
| --- | --- | --- | --- |
| β2-Microglobulin | 1.19E-09 | 7.98E-08 | 11.7 |
| FSTL3 | 1.19E-09 | 7.98E-08 | 25.0 |
| Pleiotrophin | 1.19E-09 | 7.98E-08 | 15.3 |
| TNF sR-I | 1.19E-09 | 7.98E-08 | 48.3 |
| Factor D | 4.77E-09 | 2.13E-07 | 24.4 |
| IL-15 Rα | 4.77E-09 | 2.13E-07 | 25.0 |
| MMP-7 | 8.35E-09 | 3.19E-07 | 19.1 |
| Angiopoietin-2 | 1.43E-08 | 3.48E-07 | 55.1 |
| Cystatin C | 1.43E-08 | 3.48E-07 | 13.3 |
| HCC-1 | 1.43E-08 | 3.48E-07 | 8.7 |
| URB | 1.43E-08 | 3.48E-07 | 105.7 |
| Lysozyme | 3.58E-08 | 7.36E-07 | 14.7 |
| ROR1 | 3.58E-08 | 7.36E-07 | 101.2 |
| Chordin-Like 1 | 5.37E-08 | 1.03E-06 | 48.8 |
| Endostatin | 7.99E-08 | 1.43E-06 | 20.1 |
| Ephrin-A5 | 1.66E-07 | 2.61E-06 | 23.9 |
| Matrilin-2 | 1.66E-07 | 2.61E-06 | 104.4 |
| IGFBP-6 | 4.42E-07 | 6.56E-06 | 22.6 |
| Granzyme B | 5.98E-07 | 8.41E-06 | 25.5 |
| DAN | 1.06E-06 | 1.42E-05 | 17.7 |
| β-NGF | 1.39E-06 | 1.69E-05 | 13.5 |
| Nectin-like protein 2 | 1.39E-06 | 1.69E-05 | 48.6 |
| CXCL16, soluble | 1.82E-06 | 2.11E-05 | 24.2 |
| IGFBP-2 | 2.35E-06 | 2.51E-05 | 31.3 |
| SLPI | 2.35E-06 | 2.51E-05 | 11.7 |
| TGF-β R III | 3.84E-06 | 3.95E-05 | 91.3 |
| CNTFR α | 4.87E-06 | 4.66E-05 | 35.8 |
| Lymphotoxin α1/β2 | 4.87E-06 | 4.66E-05 | 69.4 |
| CD48 | 7.51E-06 | 6.64E-05 | 22.3 |
| Lymphotoxin β R | 7.69E-06 | 6.64E-05 | 43.7 |
| Troponin I | 7.69E-06 | 6.64E-05 | 23.9 |
| ESAM | 1.19E-05 | 9.63E-05 | 38.1 |
| NovH | 1.19E-05 | 9.63E-05 | 35.7 |
| HCC-4 | 1.47E-05 | 1.15E-04 | 11.2 |
| CD30 Ligand | 1.80E-05 | 1.30E-04 | 26.0 |
| MIA | 1.80E-05 | 1.30E-04 | 12.1 |
| BCMA | 2.20E-05 | 1.44E-04 | 20.1 |
| Insulysin | 2.20E-05 | 1.44E-04 | 117.9 |
| Thrombospondin-1 | 2.20E-05 | 1.44E-04 | 128.0 |
| Trypsin | 2.20E-05 | 1.44E-04 | 24.1 |
| Cystatin M | 2.69E-05 | 1.71E-04 | 13.6 |
| Bcl-2 | 3.26E-05 | 1.89E-04 | 26.3 |
| Kallikrein 6 | 3.26E-05 | 1.89E-04 | 24.5 |
| LSAMP | 3.26E-05 | 1.89E-04 | 31.8 |
| NKG2D | 3.26E-05 | 1.89E-04 | 25.3 |
| Desmoglein-1 | 3.94E-05 | 2.07E-04 | 107.7 |
| Dtk | 3.94E-05 | 2.07E-04 | 92.8 |
| EphA1 | 3.94E-05 | 2.07E-04 | 106.0 |
| MIP-3β | 3.94E-05 | 2.07E-04 | 8.8 |
| Myoglobin | 3.94E-05 | 2.07E-04 | 17.0 |
| Biglycan | 4.74E-05 | 2.31E-04 | 37.2 |
| CD30 | 4.74E-05 | 2.31E-04 | 61.9 |
| IL-17D | 4.74E-05 | 2.31E-04 | 20.3 |
| MIP-5 | 4.74E-05 | 2.31E-04 | 10.2 |
| Fractalkine/CX3CL-1 | 5.69E-05 | 2.72E-04 | 39.6 |
| IL-18 BPa | 6.81E-05 | 3.19E-04 | 17.6 |
| GA733-1 protein | 8.11E-05 | 3.68E-04 | 33.1 |
| Galectin-3 | 8.11E-05 | 3.68E-04 | 26.1 |
| bFGF-R | 9.64E-05 | 4.23E-04 | 89.4 |
| PECAM-1 | 9.64E-05 | 4.23E-04 | 79.6 |
